# Downregulation of SPINK5 promotes laryngeal cancer cell malignancy through activation of KLK6-dependent glycolysis

**DOI:** 10.1186/s12885-026-15958-8

**Published:** 2026-04-30

**Authors:** Jianing Sun, Wudan He, Shanshan Sun, Dijiang Ma, Lili Fang

**Affiliations:** Yangming Hospital Affiliated to Ningbo University (Yuyao People’s Hospital), Zhejiang, 315400 China

**Keywords:** Laryngeal cancer, SPINK5, Glycolysis, KLK6, Tumor progression

## Abstract

**Background:**

Laryngeal cancer remains a significant health challenge with limited therapeutic options. Serine protease inhibitor Kazal type 5 (SPINK5) is implicated as a tumor suppressor in head and neck cancers, yet its role in laryngeal cancer progression and metabolic reprogramming remains unclear. This study aimed to define SPINK5’s function and molecular mechanism in laryngeal cancer progression.

**Methods:**

Analysis of The Cancer Genome Atlas (TCGA) data and immunohistochemistry was conducted to investigate the expression level of SPINK family members in laryngeal cancer tissues compared to adjacent non-tumorous tissues. Besides, western blotting, CCK-8, colony formation, and migration assay were used to investigate the expression of SPINK5, Glucose Transporter 1 (GLUT1), and Hexokinase II (HK-II), and growth and migration capacities of laryngeal cancer cells. While glucose uptake and lactate secretion detection were conducted to measure the glycolysis level of laryngeal cancer cells. And differences among groups were analyzed using Student’s t-test or one-way analysis of variance.

**Results:**

The downregulation of SPINK5 was observed in laryngeal cancer tissues compared to adjacent non-tumorous tissues. SPINK5 acted as a tumor suppressor, inhibiting laryngeal cancer cell growth and migration. Mechanistically, SPINK5 knockdown promoted glycolysis through GLUT1 and HK-II upregulation. Notably, SPINK5 could interact with kallikrein-related peptidase 6 (KLK6). SPINK5 knockdown enhanced the activity of KLK6 and the activation of the PI3K/AKT/mTOR signaling pathway in laryngeal cancer cells. Critically, silencing KLK6 reversed the oncogenic effects induced by SPINK5 knockdown, including PI3K/AKT/mTOR pathway activation, enhanced glycolysis, and accelerated cell growth and migration.

**Conclusions:**

These findings identify a novel SPINK5-KLK6-glycolysis regulatory axis driving laryngeal cancer progression. Our results highlight SPINK5 as a tumor suppressor gene for laryngeal cancer progression, providing new insights into its molecular pathogenesis.

**Supplementary Information:**

The online version contains supplementary material available at 10.1186/s12885-026-15958-8.

## Introduction

Laryngeal cancer is one of the most common malignancies within head and neck squamous cell carcinomas and represents the second most common respiratory tract cancer after lung cancer [1]. Despite advances in treatment modalities, including surgical resection, radiotherapy, chemotherapy, and immunotherapy, therapeutic outcomes remain limited due to tumor recurrence, metastasis, and resistance to radio-chemotherapy, contributing to a persistently low five-year survival rate below 40% [2, 3]. Consequently, exploring novel pathogenic mechanisms and developing innovative therapeutic strategies for laryngeal cancer are of significant clinical importance.

The Serine peptidase inhibitor Kazal type 5 (SPINK5) gene encodes the lymphoepithelial Kazal-type-related protease inhibitor (LEKTI), deficiency of which is closely associated with Netherton syndrome [4], a severe autosomal recessive skin disorder. Recent studies indicate that SPINK5 is abnormally expressed in head and neck cancers and serves as an independent prognostic predictor for patients with these malignancies [5]. Although bioinformatic analyses suggest that SPINK5 may function as a biomarker linked to laryngeal squamous cell carcinoma (LSCC, the histologic subtype in greater than 90% of laryngeal cancers) progression and prognosis [6], its precise biological roles and molecular regulatory mechanisms in laryngeal cancer cell malignancy remain elusive.

Notably, cancer cells exhibit a metabolic shift characterized by avid glucose uptake and lactate secretion even under aerobic conditions, a phenomenon termed aerobic glycolysis or the Warburg effect. Beyond energy production, glycolytic metabolites contribute to macromolecular biosynthesis and acidify the tumor microenvironment, conferring selective advantages under nutrient stress [7–9]. Studies confirm that glycolysis is hyperactivated in LSCC, and glycolysis-related gene expression signatures can predict the prognosis of LSCC patients [10–12]. Our prior work further demonstrated that inhibiting glycolysis can enhance the radiosensitivity in laryngeal cancer, underscoring the pivotal role of this metabolic pathway in laryngeal cancer progression [13, 14]. Although a direct link between SPINK5 and glycolysis has not been established, SPINK5 is a known regulator of the kallikrein-related peptidase family (KLKs) activity [15]. Several KLKs have been implicated in glycolytic regulation across cancers [16, 17]. For instance, KLK6 enhances glycolytic metabolism in gastric cancer cells [16]. KLK10 increases glycolytic capacity in colorectal cancer cells [17]. These evidences suggest that SPINK5 may modulate glycolysis in laryngeal cancer cells via KLK-dependent mechanisms, a hypothesis requiring experimental validation.

In this study, we analyzed the expression of SPINK family members in laryngeal cancer tissues through The Cancer Genome Atlas (TCGA) datasets and systematically evaluated SPINK5 expression level in laryngeal cancer tissues, assessed the functional impact of SPINK5 on the growth and migration capacities of laryngeal cancer cells, and elucidated its potential molecular mechanisms. We demonstrate that downregulation of SPINK5 promotes malignant behaviors of laryngeal cancer cells by activating KLK6-dependent glycolysis. These findings provide a novel theoretical foundation for developing new therapeutic strategies for laryngeal cancer targeting the SPINK5/KLK6/glycolysis axis.

## Materials and methods

### Transcriptome data acquisition and analysis

Transcriptome profiling data (STAR-counts) and clinical information for Head and Neck Squamous Cell Carcinoma (HNSCC) from The Cancer Genome Atlas (TCGA) database (https://portal.gdc.cancer.gov). After initial processing, we specifically extracted 91 laryngeal cancer samples and 11 adjacent normal tissue samples from the dataset containing 566 HNSCC samples for downstream analysis. Gene expression values were converted to Transcripts Per Million (TPM) and normalized using the log₂ (TPM + 1) method. We analyzed the expression profiles of SPINK family genes (SPINK1 to SPINK14) in laryngeal cancer tissues versus normal controls.

### Patients and samples

Paraffin-embedded tissue sections from tumor and adjacent nontumor tissues were collected from 8 laryngeal cancer patients treated at Yuyao People’s Hospital. The tissue sections were stored at room temperature. Written informed consent was obtained from all patients, and the study was approved by the Ethics Committee of Yuyao People’s Hospital (ethical approval number: 2024-03-007).

### Immunohistochemistry (IHC)

Sections underwent antigen retrieval using a repair solution and were blocked with 3% hydrogen peroxide (Aladdin, H112515, Shanghai, CN) to inhibit endogenous peroxidase activity. Then sections were incubated overnight with primary antibodies against SPINK5 (Servicebio, GB111906-100, 1:500, Wuhan, CN). After washing with PBS, the secondary antibody (Goat Anti-Rabbit IgG (H + L) HRP, Affinity, S0001, 1: 200, Wuhan, CN) was applied for 30 min. DAB color development solution (Biosharp, BL732A, Hefei, CN) was added drop by drop, followed by counterstaining with Harris hematoxylin (Biosharp, BL702B, Hefei, CN). After the application of neutral adhesive, the samples were imaged using a microscope (Olympus, BX53, Tokyo, Japan).

### Cell culture

Immortalized Human Laryngeal Epithelial Cells (HuLa-TVC, Abm, T0850, USA) were cultured in PriGrow X Series Medium (Abm, TM0850, USA) supplemented with 1% penicillin-streptomycin (Procell, PB180120, Wuhan, CN). The human cancer cell lines, including laryngeal cancer cell lines​ TU686 (MZ-2155, Ningbo, CN), M2e (Shanghai Jining Industrial Co., Ltd., JN-2244, Shanghai, CN), and M4e (Shanghai Jining Industrial Co., Ltd., JN-2245, Shanghai, CN), pharyngeal carcinoma cell line FaDu (ATCC, HTB-43, USA), and oral squamous carcinoma cell lines CAL27 (ATCC, CRL-2095, USA) and SCC25 (ATCC, CRL-1628, USA), were cultured in DMEM (Procell, PM150210, Wuhan, CN) supplemented with 10% fetal bovine serum (FBS, Procell, 164210-500, Wuhan, CN) and 1% penicillin-streptomycin. The human laryngeal cancer cell line AMC-HN8 (NCACC, TCHu262, Shanghai, CN) was cultured in RPMI 1640 medium (GIBCO, 11875093, Shanghai, CN) with 10% FBS and 1% penicillin-streptomycin. Both cell lines were maintained in a humidified incubator at 37 °C with 5% CO₂. 

### Cell transfection

SiRNA specifically targeting SPINK5 (SPINK5 siRNA-1, siG000011005A-1-5; SPINK5 siRNA-2, siG000011005B-1-5; SPINK5 siRNA-3, siG000011005C-1-5), SPINK8 (SPINK8 siRNA-1, siG000646424A-1-5; SPINK8 siRNA-2, siG000646424B-1-5; SPINK8 siRNA-3, siG000646424C-1-5), SPINK14 (SPINK14 siRNA-1, siG000408187A-1-5; SPINK14 siRNA-2, siG000408187B-1-5; SPINK14 siRNA-3, siG000408187C-1-5), KLK6 (KLK6 siRNA-1, siG000005653A-1-5; KLK6 siRNA-2, siG000005653B-1-5; KLK6 siRNA-3, siG000005653C-1-5), and KLK10 (KLK10 siRNA-1, siG000005655A-1-5; KLK10 siRNA-2, siG000005655B-1-5; KLK10 siRNA-3, siG000005655C-1-5) were obtained from RIBOBIO (Guangzhou, CN). Synthetic DNA fragments of SPINK5 (Vectorbuilder, VB900010-6986pqj, Guangzhou, CN) were inserted into pRP[Exp]-CMV> EGFP/Neo vector. The siRNAs (50 nM) or plasmids were transfected into TU686 and AMC-HN-8 cells using Lipofectamine 3000 (Invitrogen, L3000150, Carlsbad, USA) according to the manufacturer’s protocol. After transfection for approximately 72 h, the cells were harvested for Western blot analysis of protein expression.

### Western blotting

Proteins were extracted using RIPA buffer with a protease inhibitor cocktail (Beyotime, P0013B, Shanghai, CN), quantified via a BCA protein detection kit (Beyotime, P0009), separated by 10% SDS-PAGE, and transferred onto PVDF membranes (Millipore, IPVH00010, MA, USA). The membrane was blocked with 5% non-fat milk in TBST for 1 h at room temperature and incubated overnight at 4 °C with the appropriate primary antibodies against SPINK5 (Abcam, ab138511, 1:1000, Hangzhou, CN), SPINK8 (Abgent, ap12598b, 1:1000, San Diego, USA), SPINK14 (Abnova, H00408187-K, 1:1000, Taipei, CN), GAPDH (Affinity, AF7021, 1:1000, Wuhan, CN), HK-Ⅱ (Affinity, DF6176, 1:1000, Wuhan, CN), GLUT1 (Affinity, Af6731, 1:1000, Wuhan, CN), KLK6 (Affinity, DF2704, 1:1000, Wuhan, CN), KLK10 (Abcam, ab273878, 1:1000, Hangzhou, CN), p-Akt (Affinity, AF0016, 1:1000, Wuhan, CN), Akt (Affinity, AF6261, 1:1000, Wuhan, CN), p-mTOR (Affinity, AF3308, 1:1000, Wuhan, CN), or mTOR (Affinity, AF6308, 1:1000, Wuhan, CN). After washing with TBST for three times, the membrane was incubated with HRP-labeled secondary antibodies (Affinity, S0001, 1:5000, Wuhan, CN), ) for 2 h at room temperature. The proteins were then visualized using ECL reagent (BIO-RAD, 170–5061, Hercules, USA).

### Cell viability assay

TU686 and AMC-HN8 cells were seeded into 96-well plates (2000 cells/well) and transfected with the SPINK5 plasmid or SPINK5 siRNA, or KLK6 siRNA, or KLK10 siRNA, or treated with 2-DG (5mM, MCE, HY-13966, Shanghai, CN) alone or in combination for 72 h. Then, 10 µl Cell Counting Kit − 8 (CCK-8) reagent (Beyotime, C0037, Shanghai, CN) was added to each well. After incubation in a humidified atmosphere at 37 °C with 5% CO₂ for 2 h, the absorbance at 490 nm was measured using a microplate reader (Thermo Fisher Scientific, Varioskan ALF, USA). Cell viability was normalized to the control group (100%).

### Colony formation assay

Cells were plated in 6-well plates (1 × 10^3^ cells/well), then transfected or treated as above for 72 h. After 14 days, cells were washed twice with cold PBS (Solarbio, P1020, Beijing, CN), fixed with methanol (Sigma, 322415, Shanghai, CN), and stained with 0.5% crystal violet (Sigma, C0775, Shanghai, CN). The number of colonies was counted under a microscope (OLYMPUS, CX21FS1, Tokyo, Japan).

### Migration assays

TU686 and AMC-HN8 cells were seeded in transwell inserts (Corning Costar, 3422, USA). Then, they were either transfected with the SPINK5 plasmid, SPINK5 siRNA, or KLK6 siRNA, or treated with 2-DG alone or in combination. After 72 h, the cells that had passed through the insert membrane were fixed with 4% paraformaldehyde for 15 min and then stained with 1% crystal violet for 20 min. Subsequently, the insert was removed, and the cells were stained and photographed under a microscope.

### Glucose uptake assays

The glucose uptake ability of TU686 and AMC-HN8 cells was evaluated by using the fluorescent glucose 2-NBDG (Thermo Fisher Scientific, N13195, Shanghai, CN). TU686 and AMC-HN8 cells seeded in 96-well plates were cultured in DMEM medium without glucose or carbon sources. After being transfected with the SPINK5 plasmid, or SPINK5 siRNA, or KLK6 siRNA, or KLK10 siRNA, or treated with 2-DG individually, or in combination, for 72 h, the cells were gently rinsed with HBSS and incubated with 100 µM 2-NBDG at 37 °C for 30 min. Consequently, the cells were rewashed with HBSS. Fluorescent intensity of the cells was detected on a microplate reader (Ex (λ) 465 nm; Em (λ) 540 nm). The glucose uptake ability was normalized to the control group (100%).

### Detection for lactate

For lactate content quantification, TU686 and AMC-HN8 cells were seeded into 6-well plates at a density of 3 × 10⁵ cells/well and cultured for 24 h. After 24 h, the cells were either transfected with the SPINK5 plasmid, SPINK5 siRNA, KLK6 siRNA, or KLK10 siRNA or treated with 2-DG individually or in combination for 72 h. The supernatant was then analyzed using the Lactate Assay Kit (BC2235, Solarbio, Beijing, CN) according to the manufacturer’s instructions.

### KLK6 assays

To detect KLK6 activity in cell lysates, first centrifuge the lysate at 12,000 × g for 10 min at 4 °C to pellet debris. Add 50 µL of the clarified lysate to each well of a 96-well plate, along with 50 µL of reaction buffer (50 mM Tris (pH 7.5), 150 mM NaCl, 1 mM EDTA, and 0.05% Tween-20). Add 100 µL of the fluorescent substrate N-Boc-Phe-Ser-Arg-7-amino-4-methylcoumarin (FSR-AMC, BACHEM, CH) to each well to achieve a final concentration of 20 µM. Seal the plate, incubate at 37 °C for 30 min, and detect the fluorescent intensity on a microplate reader (Ex (λ) 350 nm; Em (λ) 440 nm). The activity of KLK6​ was normalized to the control group (100%).

### Co-immunoprecipitation (Co-IP) assay

Cell lysates from 5 × 10^6^ cells were prepared using the IP buffer (Beyotime, P0013, Shanghai, CN). The lysates were washed with 100 µL of protein A/G agarose beads (Thermo Scientific Pierce Co-IP kit, 88804 IL, USA) in 1 mL lysis buffer. The lysates were then incubated with anti-SPINK5 antibody (Santa Cruz, sc-32330, CA, USA), or anti-IgG (CST, 5415, Wuhan, CN) overnight with the protein A/G agarose beads. The complexes were washed three times with lysis buffer and resuspended in 2× SDS loading buffer. The immunoprecipitated proteins were eluted from the beads by incubation at 95 °C for 5 min. The eluted proteins were analyzed by Western blotting.

### Animals and treatment

The five-week-old male nude BALB/C mice utilized in this study were obtained from Sibeifu (Beijing) Biotechnology Co., Ltd. All experiments were conducted according to the Animal Research: Reporting of In Vivo Experiments (ARRIVE) guidelines and were approved by the Laboratory Animal Welfare & Ethics Committee of the Bestcell Model Biological Center (ethical approval number: 2025-11-28B). The mice were housed in a room with a 12-hour light/dark cycle and a temperature maintained at 24 ± 0.5 ℃. Twelve mice were randomly allocated into 2 groups, consisting of 6 mice in each group. A total of 2 × 10^6^ TU686 or SPINK5-stably transfected TU686​ cells in 100 µL PBS were injected into the right axilla of the mice. The nude mice were consistently fed, and their body weight, tumor length, and width were measured twice a week. Tumor volume was calculated using the formula: tumor volume = 1/2 × length × width^2^. On day 21 post-inoculation, the mice were anesthetized with pentobarbital sodium (Sigma, 57-33-0, 50 mg/kg) before being euthanized via cervical dislocation. The tumors that developed subcutaneously in mice were excised, rinsed with PBS, photographed, and their sizes and weights were measured.

### Statistical analysis

All data were shown as mean ± SD, and all experiments were performed at least three times independently. Differences among groups were analyzed using Student’s t-test or one-way analysis of variance (ANOVA) with SPSS (version 25.0, Chicago, USA). *P* < 0.05 indicates a significant difference among groups.

## Results

### SPINK5 expression is significantly decreased in laryngeal cancer

**Fig. 1:**
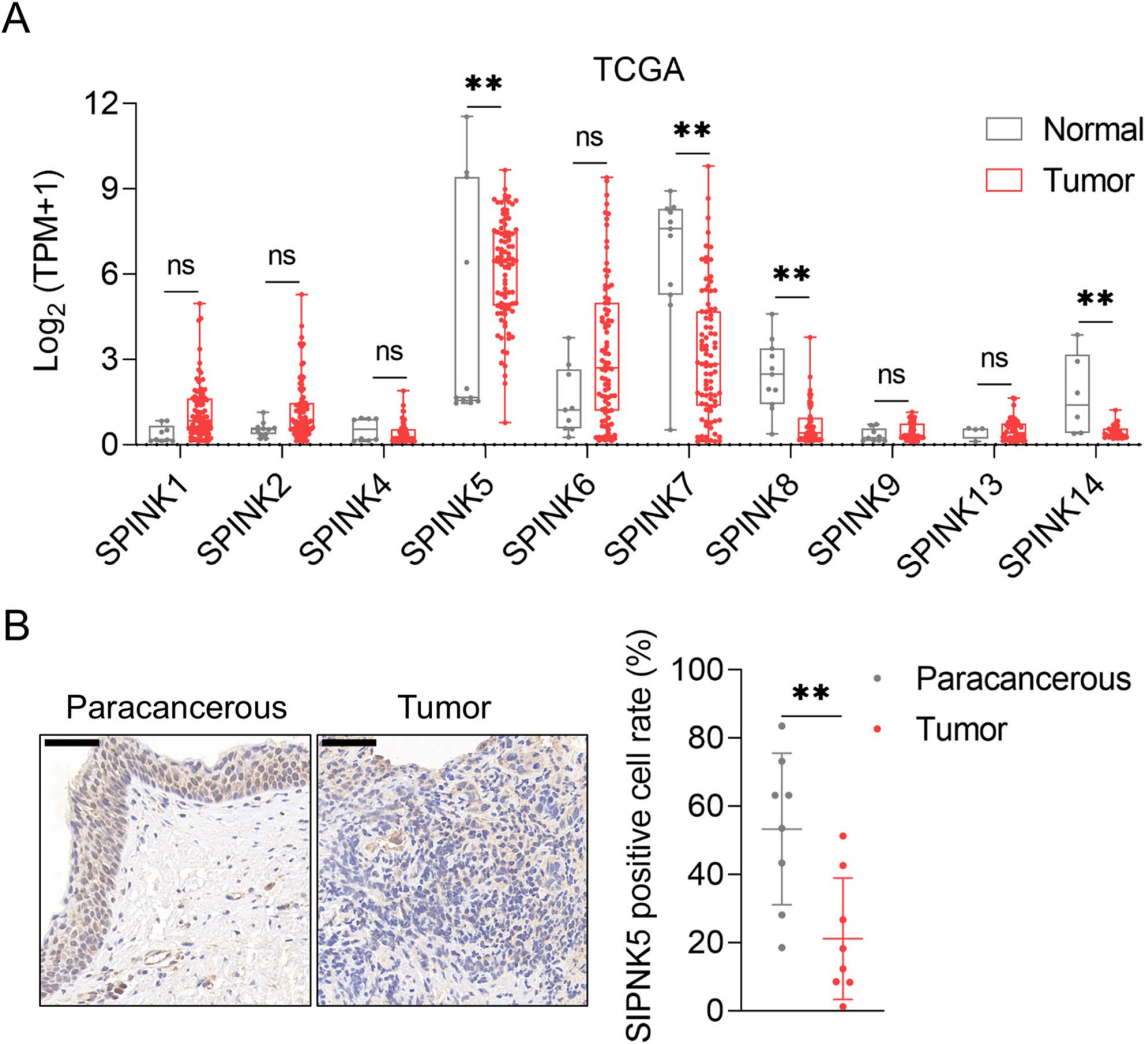
Downregulated SPINK5 is observed in laryngeal cancer tissues. (**A**) Expression of SPINK family members in 91 laryngeal cancer tissues and 11 adjacent normal tissues from the TCGA database. (**B**) Immunohistochemical staining of SPINK5 protein in 8 pairs of laryngeal cancer tissues and adjacent non-tumorous tissues. Quantitative data are presented as mean ± SD. Group differences were assessed by Student’s t-test, with ** indicating *P* < 0.01 and ns indicating *P* > 0.05. Scale bar: 50 μm (B)

To characterize the expression pattern of SPINK family members in laryngeal cancer, we analyzed transcriptomic data from 91 laryngeal cancer samples and 11 adjacent normal tissue samples from the TCGA database. It revealed that multiple SPINK family members (excluding SPINK3, SPINK10, SPINK11, and SPINK12) were detectable in these tissues (Fig. 1A). Notably, SPINK5 (fold change = 0.097), SPINK7 (fold change = 0.179), SPINK8 (fold change = 0.084), and SPINK14 (fold change = 0.051) were significantly downregulated in laryngeal cancer tissues compared to adjacent non-tumorous tissues (Fig. 1A). Among SPINK5, SPINK8, and SPINK14, exhibiting the most pronounced downregulation, only SPINK5 was previously implicated as a tumor suppressor gene [17]. Then, IHC analysis of 8 paired clinical specimens further confirmed that SPINK5 protein expression was markedly reduced in laryngeal cancer tissues relative to adjacent non-tumorous tissues (Fig. 1B). These findings collectively indicate that SPINK5 is downregulated in laryngeal cancer tissues.

### SPINK5 suppresses the proliferation and migration of laryngeal cancer cells

**Fig. 2:**
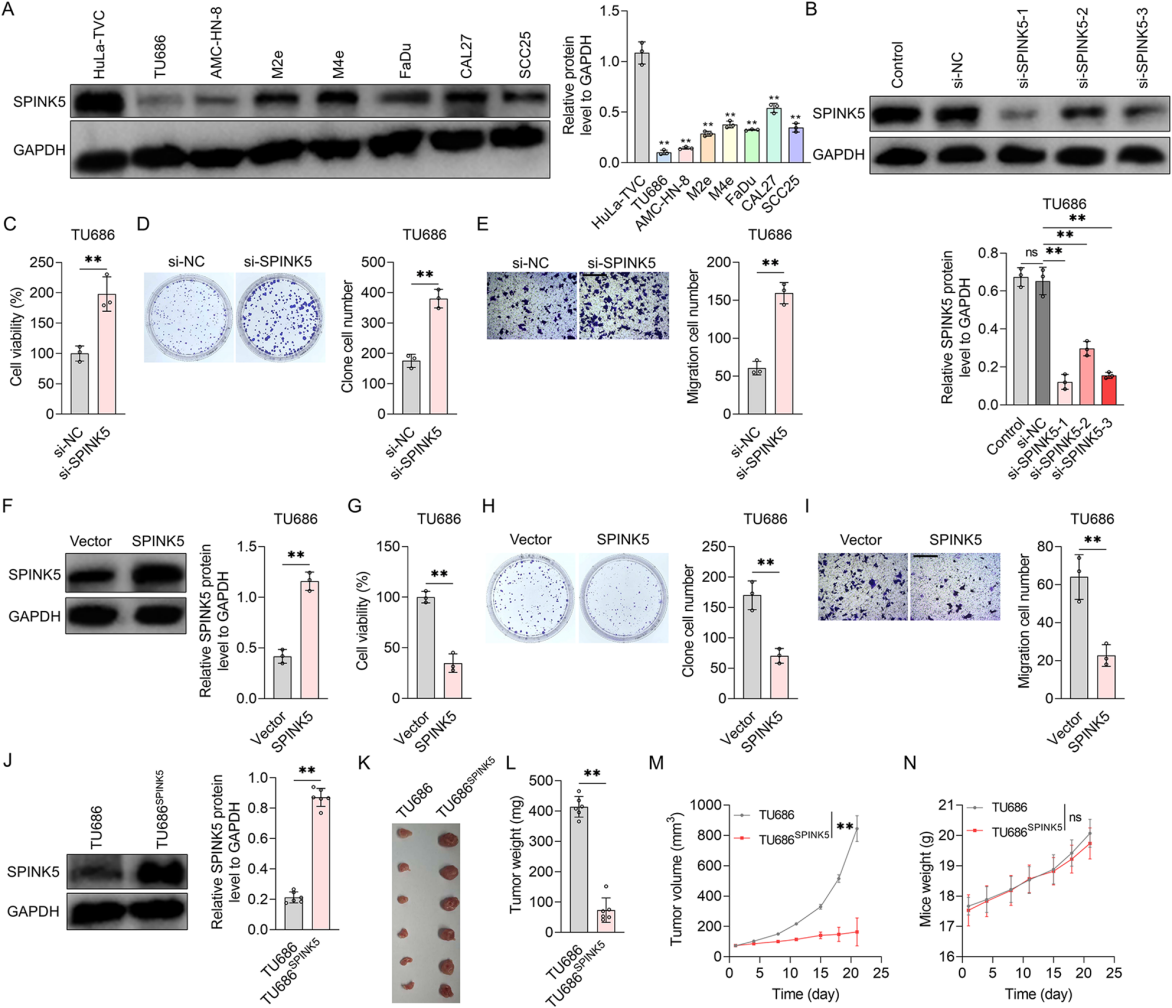
Effects of SPINK5 on the growth and migration of TU686 cells. (**A**) The protein level of SPINK5 was detected by Western blotting in HuLa-TVC and a panel of HNSCC cell lines (TU686, AMC-HN-8, M2e, M4e, FaDu, CAL27, and SCC25), with GAPDH serving as the loading control. (**B**-**E**) TU686 cells were transfected with three different SPINK5 siRNAs for 72 h. Then, (**B**) the expression of SPINK5 was detected by Western blot analysis using GAPDH as the loading control, (**C**) cell viability was measured by CCK-8 assay, (**D**) colony formation ability was assessed by plate colony formation assay, and (**E**) cell migration was evaluated by transwell assay. (F-I) TU686 cells were transfected with SPINK5 plasmid for 72 h. Then, (**F**) SPINK5 protein expression was detected by Western blot analysis using GAPDH as the loading control, (**G**) cell viability was measured by CCK-8 assay, (**H**) colony formation ability was assessed by plate colony formation assay, and (**I**) cell migration was evaluated by transwell assay. (**J**) The protein level of SPINK5 was detected by Western blotting in TU686 and SPINK5-stably transfected TU686 cells, with GAPDH serving as the loading control. (**K**-**N**) To establish a xenograft model, TU686 cells and SPINK5-stably transfected TU686 cells were subcutaneously injected into nude mice, the mice were sacrificed 21 days post-inoculation, (**K**) the resulting tumors were harvested and photographed, (**L**) tumor weight was measured at the end of the experiment, (**M**) tumor volume and (**N**) mouse body weight were measured twice a week. Quantitative data are presented as mean ± SD (*n* = 3). Student’s t-test was used for two-group comparisons, and one-way ANOVA was used for multi-group comparisons, with ​**​ indicating *P* < 0.01 and ns indicating *P* > 0.05. Scale bar: 100 μm (**E** and **I**)

Western blotting assay was conducted to measure the protein level of SPINK5 in HuLa-TVC and a panel of HNSCC cell lines. It was found that compared with HuLa-TVC, SPINK5 was downregulated in laryngeal cancer cell lines (TU686, AMC-HN-8, M2e and M4e), pharyngeal carcinoma cell (FaDu), and oral squamous carcinoma cells (CAL27 and SCC25), with TU686 and AMC-HN-8 cells exhibiting the lowest level of SPINK5 (Fig. 2A). To elucidate the functional role of SPINK5 in laryngeal cancer cell malignant behavior, we established SPINK5-silenced models in the TU686 laryngeal cancer cell line using RNA interference technology. Western blot confirmed that three distinct SPINK5 siRNAs effectively reduced SPINK5 protein expression in TU686 cells, among which si-SPINK5-1 had the strongest effect and was used in the subsequent experiments (Fig. 2B). CCK-8 proliferation assay demonstrated that SPINK5 silencing significantly enhanced the viability of TU686 cells (Fig. 2C). Plate colony formation assay further confirmed that SPINK5 siRNA treatment markedly increased the long-term proliferative capacity of TU686 cells (Fig. 2D). Additionally, the transwell migration assay revealed that SPINK5 silencing significantly promoted the migratory capacity of TU686 cells (Fig. 2E). To validate these observations, we further constructed a SPINK5 overexpression plasmid, and western blot results confirmed the substantial upregulation of SPINK5 protein expression after transfection (Fig. 2F). In contrast to SPINK5 silencing, SPINK5 overexpression significantly reduced TU686 cell viability (Fig. 2G), colony formation ability (Fig. 2H), and migration capacity (Fig. 2I). Moreover, SPINK5 overexpression inhibited the cell viability, and migration ability of AMC-HN-8 cells (Figure S2A-S2B). Furthermore, compared with control TU686 cells, TU686 cells overexpressing SPINK5 exhibited significantly enhanced tumor growth in vivo, as evidenced by increased tumor volume and weight in tumor-bearing mice (Fig. 2J and M). In contrast, there was no significant difference in body weight between mice bearing control TU686 cells and those bearing SPINK5-overexpressing cells (Fig. 2N). Notably, siRNA-mediated knockdown of SPINK8 or SPINK14 (Figure S1A-S1B) had no significant effects on the viability of TU686 cells (Figure S1C). Collectively, these results demonstrate that SPINK5, rather than SPINK8 or SPINK14, functions as a tumor suppressor that inhibits proliferation and migration in laryngeal cancer cells.

### SPINK5 attenuates glycolysis in laryngeal cancer cells by regulating key glycolytic enzymes

**Fig. 3:**
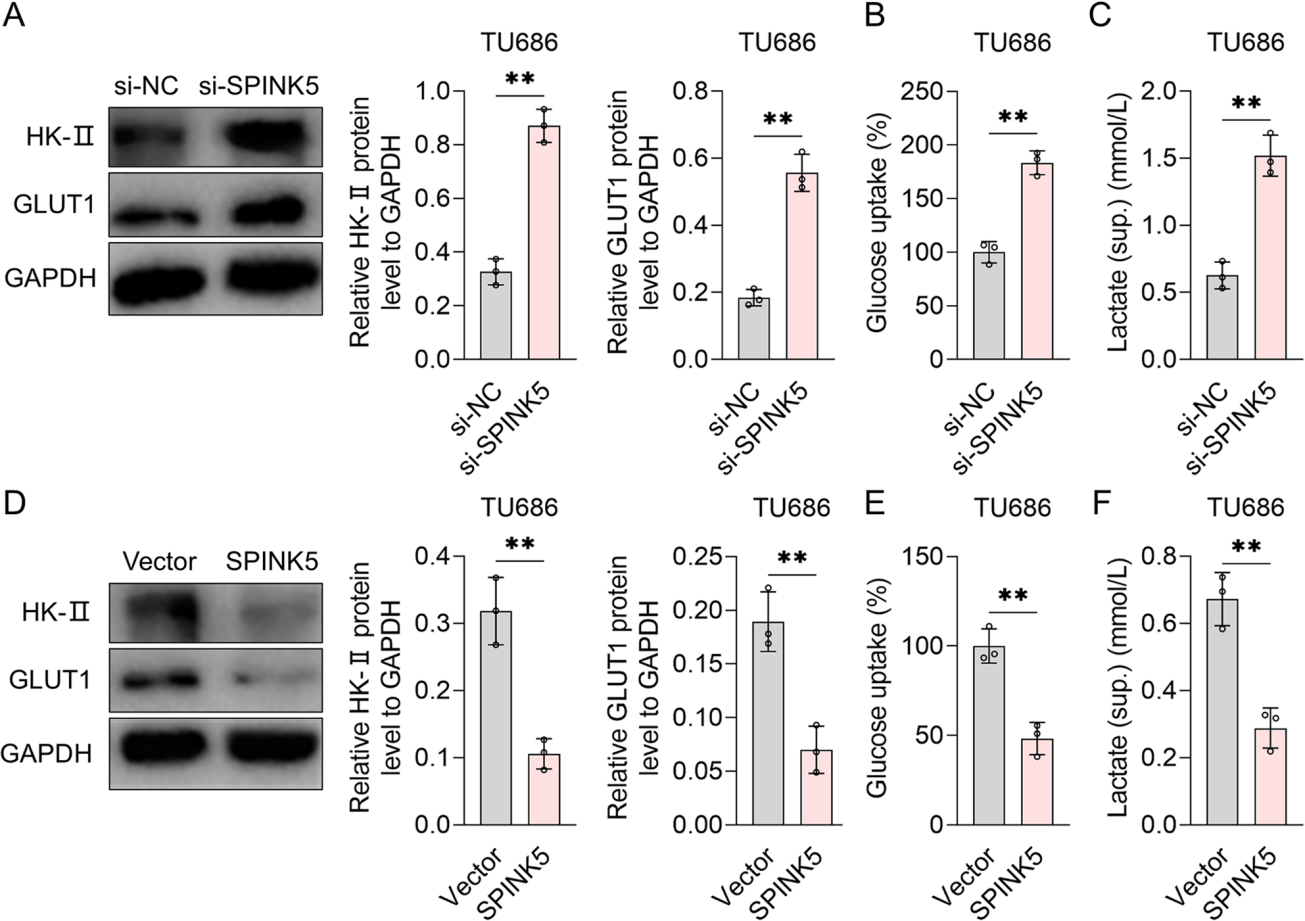
Effects of SPINK5 on glycolysis in TU686 cells. (**A**-**C**) TU686 cells were transfected with SPINK5 siRNA for 72 h. Then, (**A**) protein expressions of HK-Ⅱ and GLUT1 were detected by Western blot analysis using GAPDH as the loading control, (**B**) glucose uptake was measured by 2-NBDG assay, and (**C**) lactate levels in cell supernatants were detected using a commercial kit. (**D**-**F**) TU686 cells were transfected with SPINK5 plasmid for 72 h. Then, (**D**) protein expressions of HK-Ⅱ and GLUT1 were detected by Western blot analysis using GAPDH as the loading control, (**E**) glucose uptake was measured by 2-NBDG assay, and (**F**) lactate levels in cell supernatants were detected using a commercial kit. Quantitative data are presented as mean ± SD (*n* = 3). Group differences were analyzed by one-way ANOVA, with ** indicating *P* < 0.01

Given the central roles of Glucose Transporter 1 (GLUT1) and Hexokinase II (HK-II) in tumor glycolysis [18], we investigated the effects of SPINK5 on the expression of these key glycolytic enzymes. Western blot revealed that SPINK5 siRNA transfection significantly upregulated the protein expression levels of GLUT1 and HK-II in TU686 cells (Fig. 3A). Using the fluorescently labeled glucose analog 2-NBDG to assess cellular glucose uptake capacity, we found that SPINK5 silencing significantly enhanced glucose uptake in TU686 cells (Fig. 3B). Concurrently, lactate measurement in culture supernatants revealed that SPINK5 siRNA treatment significantly enhanced lactate secretion compared to the control group (Fig. 3C). Conversely, SPINK5 overexpression reduced the protein expression levels of GLUT1 and HK-II in TU686 cells (Fig. 3D) and significantly inhibited cellular glucose uptake (Fig. 3E) and lactate production (Fig. 3F). Moreover, cellular glucose uptake and lactate production was significantly suppressed in AMC-HN-8 with SPINK5 overexpression (Figure S2C-S2D). These findings demonstrate that SPINK5 negatively regulates glycolysis in laryngeal cancer cells.

### Glycolysis inhibition reverses the promotive effects of SPINK5 silencing on laryngeal cancer cell malignant behavior

**Fig. 4:**
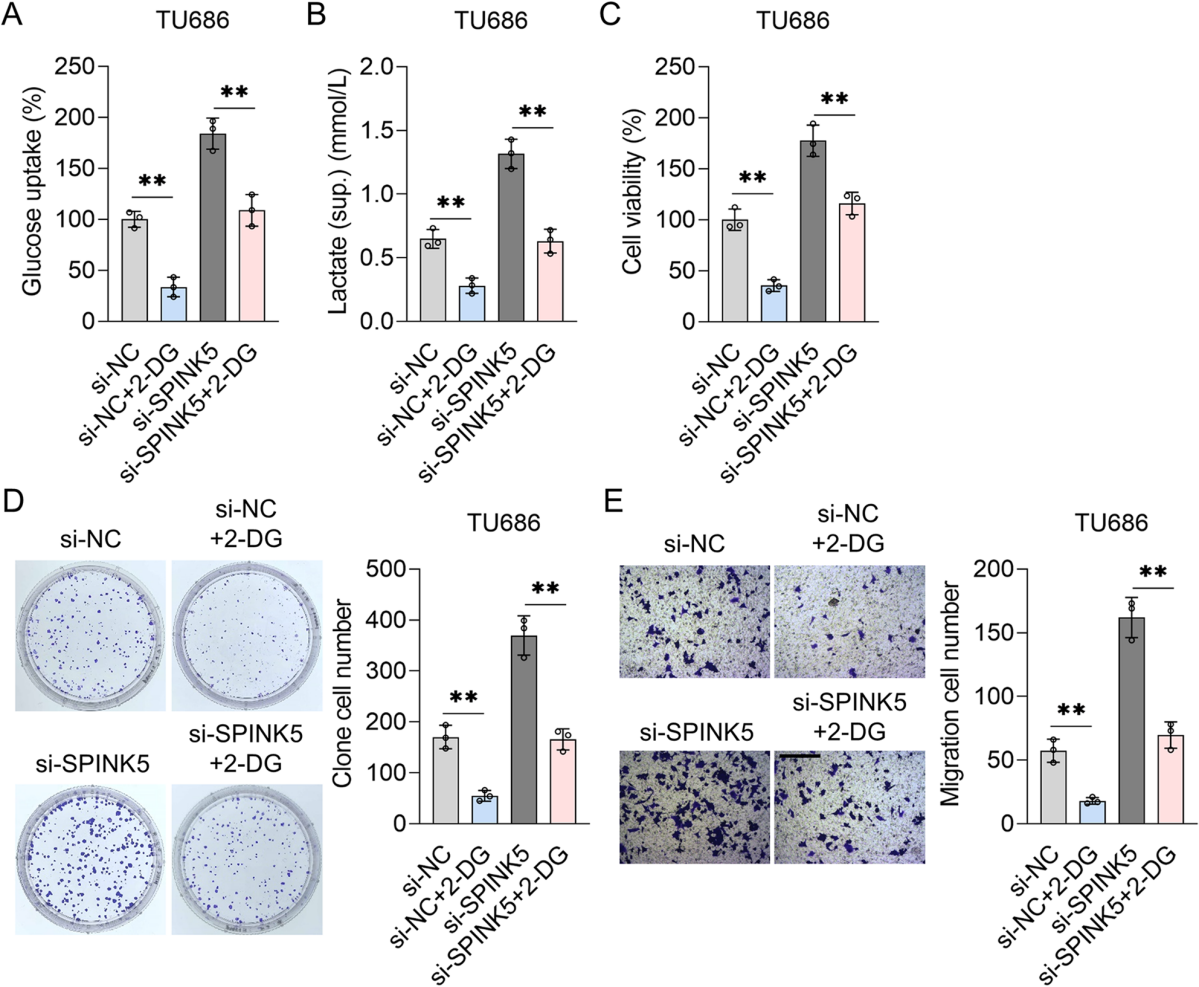
The effects of SPINK5 on TU686 cell growth and migration are dependent on glycolysis.(**A**-**E**) TU686 cells were transfected with SPINK5 siRNA, or treated with 2-DG (5mM), or both, for 72 h. Then, (**A**) glucose uptake was measured by 2-NBDG assay, (**B**) lactate levels in cell supernatants were detected using a commercial kit, (**C**) cell viability was measured by CCK-8 assay, (**D**) colony formation ability was assessed by plate colony formation assay, and (**E**) cell migration was evaluated by transwell assay. Quantitative data are presented as mean ± SD (*n* = 3). Group differences were analyzed by one-way ANOVA, with ** indicating *P* < 0.01. Scale bar: 100 μm (**E**)

To determine whether glycolysis is involved in SPINK5-mediated suppressive effects of laryngeal cancer cell malignant behavior, we treated TU686 cells with the glycolysis inhibitor 2-deoxyglucose (2-DG) alongside SPINK5 siRNA. The glucose uptake assay showed that 2-DG not only significantly inhibited the glucose uptake capacity of TU686 cells, but also reversed the enhanced glucose uptake induced by SPINK5 silencing (Fig. 4A). Lactate secretion measurement results also demonstrated that 2-DG could block the increased lactate production caused by SPINK5 silencing (Fig. 4B). In functional assays, CCK-8 results showed that 2-DG significantly reduced TU686 cell viability and abolished the promoting effect of SPINK5 silencing on cell viability (Fig. 4C). Plate colony formation and transwell migration assays further confirmed that 2-DG not only inhibited the colony formation capacity (Fig. 4D) and migration ability (Fig. 4E) of TU686 cells, but also effectively reversed the enhancement of these malignant phenotypes induced by SPINK5 silencing. These results indicate that SPINK5 constrains the proliferation and migration capacities of laryngeal cancer cells by inhibiting glycolysis.

### SPINK5 silencing activates glycolysis and PI3K/AKT/mTOR signaling pathway in laryngeal cancer cells via a KLK6-dependent manner

**Fig. 5:**
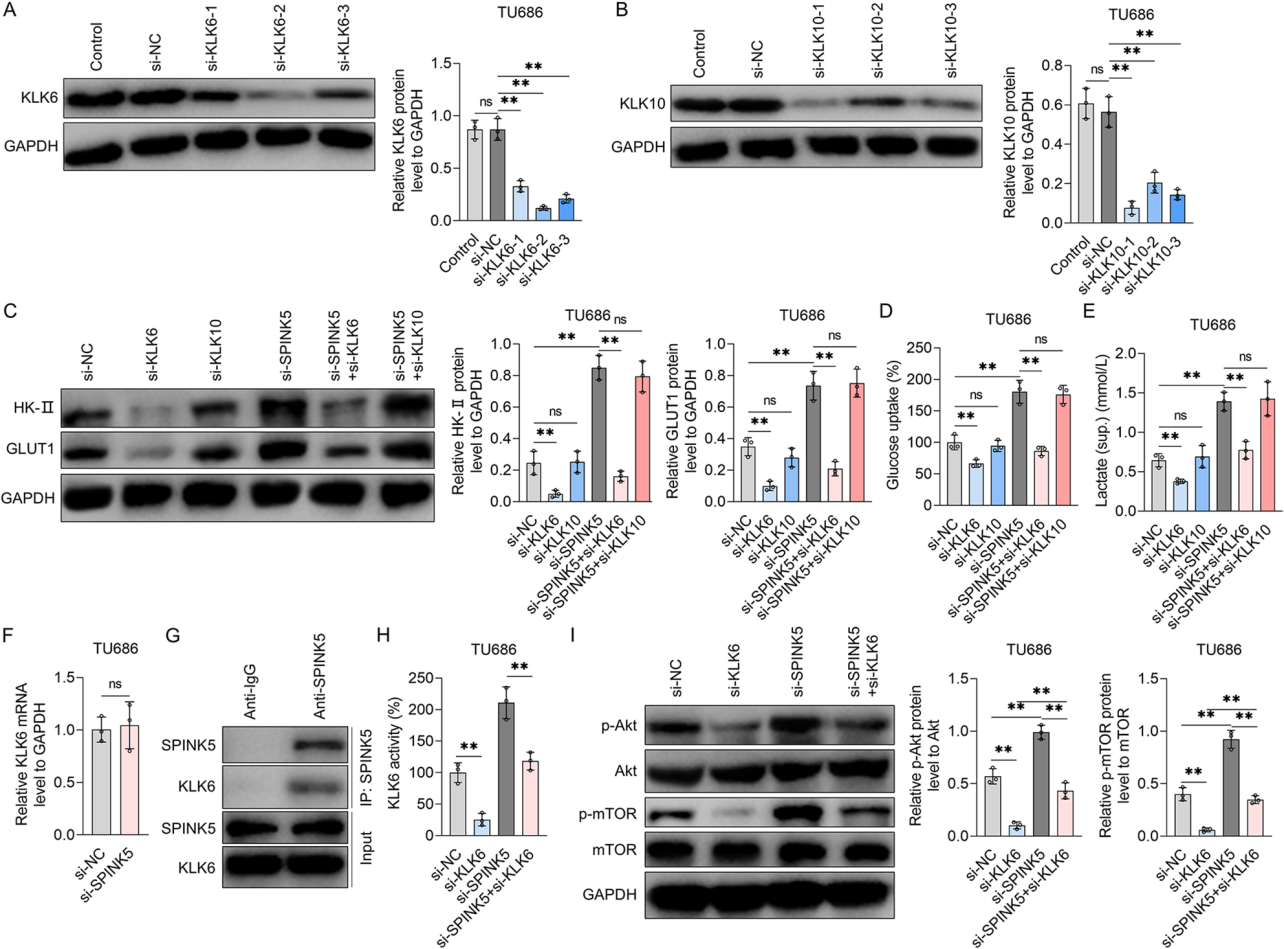
SPINK5 regulates glycolysis and PI3K/AKT pathway in TU686 cells depending on KLK6.(**A**-**B**) TU686 cells were transfected with three different KLK6 siRNAs or KLK10 siRNA, for 72 h, and protein expressions of KLK6 (**A**) and KLK10 (**B**) were detected by Western blot analysis using GAPDH as the loading control. (**C**-**E**) TU686 cells were transfected with KLK6 siRNA, KLK10 siRNA, or SPINK5 siRNA, or in combination, for 72 h. Then, (**C**) protein expressions of HK-Ⅱ and GLUT1 were detected by Western blot analysis using GAPDH as the loading control, (**D**) glucose uptake was measured, and (**E**) lactate levels in cell supernatants were detected. (**F**) TU686 cells were transfected with SPINK5 siRNA, for 72 h, and mRNA expressions of KLK6 were detected by qRT-PCR using GAPDH as the loading control. (**G**) TU686 cells were incubated with IgG antibody or SPINK5 antibody, followed by a Co-IP assay. The co-precipitated proteins SPINK5 and KLK6 were then detected by Western blot analysis. (**H**-**I**) TU686 cells were transfected with KLK6 siRNA, or SPINK5 siRNA, or both, for 72 h. Then, (**H**) KLK6 activity was measured, and (**I**) the phosphorylation levels of AKT and mTOR were detected by western blot using GAPDH as the loading control. Quantitative data are presented as mean ± SD (*n* = 3). Group differences were analyzed by one-way ANOVA, with ** indicating *P* < 0.01 and ns indicating *P* > 0.05

To dissect the mechanism linking SPINK5 to glycolysis, we constructed siRNAs targeting KLK6 and KLK10, and co-transfected them with SPINK5 siRNA into TU686 cells. Western blot analysis confirmed the effectiveness of both KLK6 siRNA and KLK10 siRNA (Fig. 5A and B). Further experiments demonstrated that KLK6 silencing significantly reduced the expression levels of GLUT1 and HK-II in TU686 cells and reversed the upregulation of these glycolytic enzymes induced by SPINK5 silencing, whereas KLK10 silencing had no such effect (Fig. 5C). Functional assays revealed that KLK6 silencing significantly inhibited glucose uptake (Fig. 5D) and lactate secretion (Fig. 5E) in TU686 cells and blocked the enhancement of these glycolytic parameters induced by SPINK5 silencing, while KLK10 silencing showed no significant impact. It was observed in qRT-PCR assay that the mRNA level of KLK6 was not significantly altered in TU686 cells treated with SPINK5 siRNA (Fig. 5F). However, an interaction of SPINK5 and KLK6 was identified in TU686 cells by Co-IP assay (Fig. 5G). Further investigation demonstrated that SPINK5 silencing significantly enhanced KLK6 activity in TU686 cells, while KLK6 siRNA suppressed both basal and SPINK5 silencing-induced KLK6 activity (Fig. 5H), confirming that SPINK5 affects KLK6 activity through direct interaction More importantly, SPINK5 knockdown significantly increased the phosphorylation level of AKT and mTOR, which was reversed by KLK6 silencing (Fig. 5I). These results indicate that SPINK5 suppresses glycolysis and the PI3K/AKT/mTOR signaling pathway in laryngeal cancer cells, dependent on KLK6.

### KLK6 silencing reverses SPINK5 silencing-driven proliferation and migration of laryngeal cancer cells

**Fig. 6:**
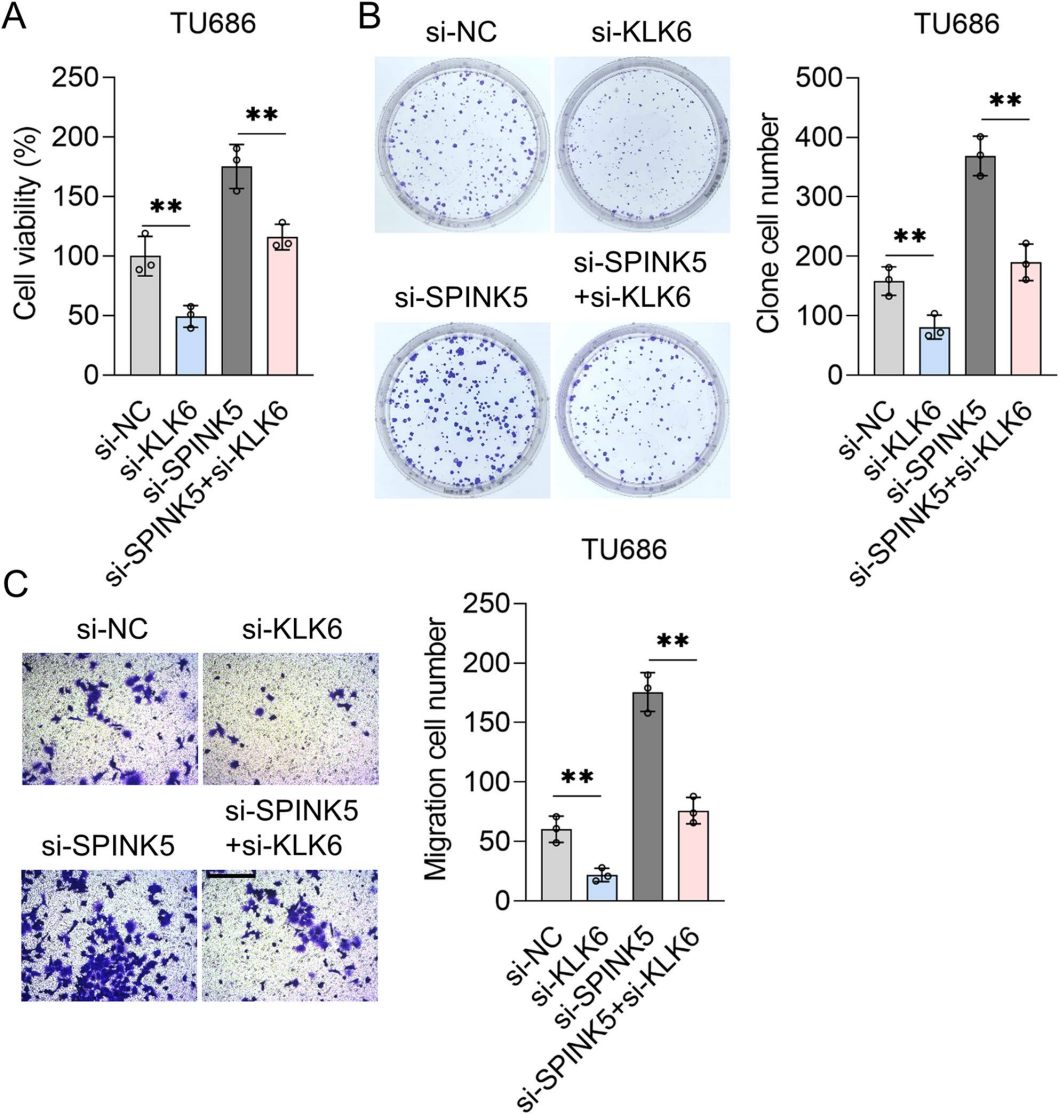
SPINK5 affects TU686 cell growth and migration via KLK6.(**A**–**C**) TU686 cells were transfected with SPINK5 siRNA, KLK6 siRNA, or both, for 72 h. Then, (**A**) cell viability was measured by CCK-8 assay, (**B**) colony formation ability was assessed by plate colony formation assay, and (**C**) cell migration was evaluated by transwell assay. Quantitative data are presented as mean ± SD (*n* = 3). Group differences were analyzed by one-way ANOVA, with ** indicating *P* < 0.01. Scale bar: 100 μm (**C**)

To further confirm the role of KLK6 in SPINK5-mediated regulation of laryngeal cancer cell malignant behavior, we co-transfected KLK6 siRNA and SPINK5 siRNA into TU686 cells. CCK-8 assay results showed that KLK6 silencing not only significantly reduced TU686 cell viability but also reversed the enhanced cell viability induced by SPINK5 silencing (Fig. 6A). Plate colony formation assay results also demonstrated that KLK6 silencing inhibited the colony formation ability of TU686 cells and blocked the upregulation of colony formation capacity caused by SPINK5 silencing (Fig. 6B). Similarly, the transwell migration assay confirmed that KLK6 silencing reversed the promotive effect of SPINK5 silencing on TU686 cell migration (Fig. 6C). These results collectively confirm that KLK6 activation is essential for SPINK5-loss-induced malignancy of laryngeal cancer cells.

### The effects of SPINK5/KLK6 axis on PI3K/AKT/mTOR pathway, glycolysis, and malignant behavior are confirmed in another laryngeal cancer cell line

**Fig. 7:**
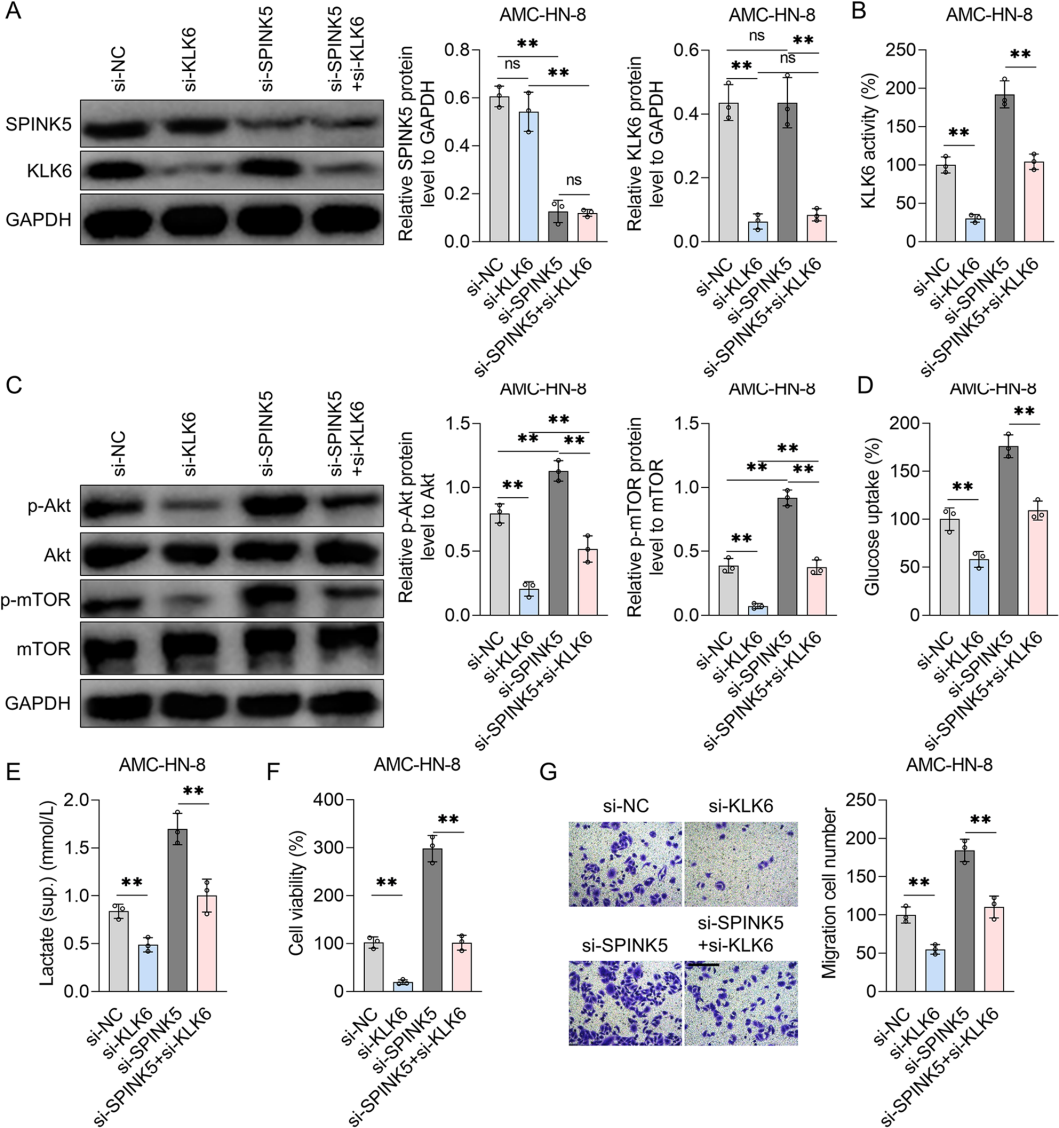
The effects of SPINK5/ KLK6 axis on PI3K/AKT pathway, glycolysis and malignant behavior were confirmed in other AMC-HN-8 cells. (**A**-**G**) AMC-HN-8 cells were transfected with SPINK5 siRNA, KLK6 siRNA, or both, for 72 h. Then, (**A**) the protein expression of SPINK5 and KLK6 was measured by western blot analysis using GAPDH as the loading control, (**B**) KLK6 activity was detected, (**C**) the phosphorylation levels of AKT and mTOR were assessed by western blot analysis using GAPDH as the loading control, (**D**) glucose uptake was evaluated, and (**E**) lactate levels in cell supernatants were detected. Meanwhile, (**F**) cell viability was measured by CCK-8 assay, and (**G**) cell migration was evaluated by transwell assay. Quantitative data are presented as mean ± SD (*n* = 3). Group differences were analyzed by one-way ANOVA, with ** indicating *P* < 0.01. Scale bar: 100 μm (**G**)

To enhance the robustness of the above findings, another laryngeal cancer cell line, AMC-HN-8, was adopted for validation. Western blot assay confirmed that SPINK5 knockdown and KLK6 siRNA treatment significantly inhibited the expression of SPINK5 and KLK6, respectively, while no significant effects of SPINK5 on KLK6 expression were also found in AMC-HN-8 cells (Fig. 7A). Another experiment demonstrated that SPINK5 silencing significantly enhanced KLK6 activity in AMC-HN-8 cells, while KLK6 siRNA suppressed KLK6 activity in AMC-HN-8 cells in the presence of NC siRNA or SPINK5 siRNA (Fig. 7B). Consistent with the results found in TU686 cells, SPINK5 knockdown significantly increased the phosphorylation level of AKT and mTOR, the effects inhibited by KLK6 siRNA treatment (Fig. 7C). Functional assays revealed that KLK6 silencing significantly inhibited glucose uptake and lactate secretion in AMC-HN-8 cells and blocked SPINK5 silencing-mediated up-regulation of glucose uptake and lactate secretion in AMC-HN-8 cells (Fig. 7D and E). Besides, CCK-8 assay results showed that KLK6 silencing not only significantly reduced cell viability, but also reversed the promoting effects of SPINK5 silencing on cell viability in AMC-HN-8 cells (Fig. 7F). More importantly, the transwell migration assay confirmed the reversal of KLK6 silencing on the enhancement of SPINK5 silencing on cell migration ability in AMC-HN-8 cells (Fig. 7G). These results collectively indicate that the SPINK5/KLK6 axis constrains the PI3K/AKT/mTOR signaling pathway, glycolysis, and the proliferation and migration capacities of laryngeal cancer cells.

## Discussion

This study elucidates the expression profile and functional significance of SPINK5 in laryngeal cancer for the first time. Analysis of TCGA data revealed that SPINK5 expression is significantly downregulated in laryngeal cancer tissues compared to adjacent non-tumorous tissues. IHC validation further confirmed that SPINK5 protein levels are markedly reduced in laryngeal cancer tissues relative to paired adjacent tissues. Besides, functional experiments demonstrated that SPINK5 suppresses the proliferation and migration capabilities of laryngeal cancer cells. The significant correlation of SPINK5 mRNA level and the prognostic value of LSCC patients was identified in TCGA cohort [6]. Collectively, SPINK5 downregulation is responsible for the malignant phenotype of laryngeal cancer cells, contributing to poor prognosis of laryngeal cancer. Intriguingly, TCGA data analysis also identified the downregulation of SPINK7, SPINK8, and SPINK14 in laryngeal tissues. However, siRNA-mediated knockdown of SPINK8 or SPINK14 had no significant effects on the growth capacity of laryngeal cancer cells, suggesting the limited functional relevance of SPINK8 and SPINK14 in laryngeal cancer cell malignancy. Moreover, SPINK7 is a recognized tumor suppressor in esophageal cancer [19]. It implies a potential inhibitory role of SPINK7 in laryngeal cancer malignant behavior, warranting further investigation.

Metabolic reprogramming represents a hallmark of cancer, with aberrant glycolytic activation constituting its most characteristic feature. In laryngeal cancer, dysregulated glycolysis is closely associated with HK-II upregulation [20] in primary laryngeal cancer tissues [21]. Our prior research further established that the adaptive survival of laryngeal cancer stem cells under hypoxia and glucose deprivation, as well as enhanced proliferation, migration, and radioresistance of laryngeal cancer cells, are mechanistically linked to GLUT1-mediated glycolytic activation [13, 14, 22, 23]. Notably, targeted inhibition of HK-II reverses glycolysis and potentiates radiotherapy sensitivity of laryngeal cancer cells [24]. The present study not only corroborates the pivotal role of glycolysis in the malignant behavior of laryngeal cancer cells but also reveals for the first time that SPINK5 suppresses the expression of GLUT1 and HK-II in laryngeal cancer cells, thereby attenuating cellular glycolytic activity and consequently impeding tumor growth and migration capacities.

Reports have revealed that KLK6 serves as an oncogene in multiple malignancies, including breast cancer [25], pancreatic cancer [26], and colorectal cancer [27], and its expression correlates with the prognosis of patients with laryngeal squamous cell carcinoma [28]. In oral squamous cell carcinoma, KLK6 expression is significantly elevated in epithelial dysplasia and tumor core regions compared to normal oral epithelium [29]. Herein, we identified KLK6 rather than KLK10 as the critical mediator of SPINK5-regulated glycolysis in laryngeal cancer cells. As a serine protease inhibitor, SPINK5 is known to directly inhibit the activity of multiple KLKs, including KLK5, KLK6, KLK7, and KLK14 [15, 30]. This study identified an interaction between SPINK5 and KLK6 in laryngeal cancer cells. SPINK5 knockdown can enhance KLK6 enzymatic activity in laryngeal cancer cells, while KLK6 silencing can reverse glycolytic activation and pro-tumorigenic phenotypes (proliferation, migration) induced by SPINK5 knockdown. This aligns with reports of KLK6-driven glycolysis in gastric cancer cells [16]. Meanwhile, this study confirmed the promoting role of KLK6 in laryngeal cancer cell growth and migration, the clinical relevance of its expression pattern in laryngeal cancer tissues, and its prognostic significance requires further investigation.

However, the mechanism by which KLK6 affects glycolysis in laryngeal cancer has not been fully elucidated. Prior research indicates that KLK6 can regulate GLUT1 and HK-II expression through the PI3K/Akt/mTOR signaling pathway, thereby activating glycolysis [31], while activation of this pathway is also closely related to laryngeal cancer progression [32, 33]. Moreover, herein, we found that KLK6 knockdown not only suppressed the expression of GLUT1 and HK-II, and restrained the PI3K/AKT/mTOR pathway, but also reversed the promoting effects of SPINK5 silencing on GLUT1 and HK-II expression and the PI3K/AKT/mTOR pathway. Therefore, we speculate that SPINK5 knockdown promotes GLUT1 and HK-II expression in laryngeal cancer cells through the PI3K/Akt/mTOR pathway, depending on KLK6, ultimately activating glycolysis; this conjecture needs to be verified. Additionally, AKT pathway-activated glycolysis in laryngeal squamous cell carcinoma also involves upregulation of other glycolytic enzymes such as PDK1 and LDHA [20], while KLK6 may further enhance glycolytic activity in laryngeal cancer cells by cleaving and activating key glycolytic enzymes such as PFK [16], hypotheses that require further verification in subsequent studies.

Besides, it has been reported that SPINK5 can antagonize the wnt/β-catenin pathway. For instance, in esophageal cancer cells, SPINK5 overexpression inhibits the wnt/β-catenin signaling pathway by reducing GSK3β phosphorylation and promoting β-catenin protein degradation, thereby suppressing tumor proliferation and invasion [34]. Similarly, SPINK5 impedes oral squamous cell carcinoma progression through wnt/β-catenin pathway inhibition [35]. Mechanistically, β-catenin transcriptionally activates c-myc-dependent glycolytic genes, including GLUT1, HK-II, PKM2, and LDHA [36], linking wnt/β-catenin signaling to metabolic reprogramming in cancer. In laryngeal cancer cells, whether the wnt/β-catenin pathway is involved in SPINK5/KLK6 axis-mediated down-regulation of GLUT1 and HK-II expression and inhibition of glycolysis remains to be explored.

Although this study elucidates the critical role of the SPINK5-KLK6-glycolysis axis in driving the malignant behavior of laryngeal cancer cells, several limitations warrant acknowledgment. First, we analyzed the expression of SPINK5 between laryngeal cancer tissues and normal tissues, but we did not investigate the association of SPINK5 protein level with clinical-pathological parameters, such as TNM stages, metastasis status, and overall survival of patients with laryngeal cancer. Although previous studies based on the TCGA database have already reported an association between SPINK5 mRNA expression and prognosis in LSCC, mRNA and protein levels are not fully concordant [6]. Therefore, it remains necessary to investigate the relationship between SPINK5 protein expression and clinical outcomes to establish SPINK5 as a prognostic biomarker for laryngeal cancer. Second, although the function of SPINK5 was confirmed through in vitro assays and in vivo experiments, and identified the role of SPINK5 in suppressing KLK6-dependent PI3K/AKT/mTOR signaling pathway and glycolysis in vitro studies, in vivo evidence of KLK6 activity, PI3K/AKT/mTOR pathway, and glycolytic alterations is lacking. In vivo validation of the mechanism would strengthen the biological relevance of our findings. In the future, clinical cohort analysis will be expanded to correlate SPINK5 protein expression with TNM staging, metastasis status, and survival of patients with laryngeal cancer, and the KLK6/PI3K/AKT/mTOR signaling pathway with glycolysis.

## Conclusion

This study reveals that SPINK5 downregulation in laryngeal cancer drives tumor progression through KLK6-mediated glycolytic reprogramming. Mechanistically, reduced SPINK5 expression enhances KLK6 protease activity, activating the PI3K/AKT/mTOR signaling pathway and glycolysis. These findings not only expand our understanding of the metabolic regulatory network in laryngeal cancer but also suggest that SPINK5 acts as a tumor suppressor gene in laryngeal cancer progression, while the SPINK5-KLK6-glycolysis axis provides new molecular targets for laryngeal cancer.

## Supplementary Information

Supplementary Material 1. Supplementary Figure 1. Effects of SPINK8 and SPINK14 on TU686 cell growth. (A-C) TU686 cells were transfected with SPINK5 siRNA or SPINK8 siRNA or SPINK14 siRNA for 72h. Then, (A) protein expression of SPINK8 and (B) SPINK14 was detected by Western blot analysis using GAPDH as the loading control, and (C) cell viability was measured by CCK-8 assay. Quantitative data are presented as mean ± SD (n = 3). Group differences were analyzed by one-way ANOVA, with **indicating *P* < 0.01 and ns indicating *P* > 0.05.

Supplementary Material 2. Supplementary Figure 2. Effects of SPINK5 on glycolysis, growth and migration of AMC-HN-8 cells. (A-D) AMC-HN-8 cells were transfected with SPINK5 plasmid for 72h. Then, (A) cell viability was measured by CCK-8 assay, (B) cell migration was evaluated by transwell assay, (C) glucose uptake was measured by 2-NBDG assay, and (D) lactate levels in cell supernatants were detected using a commercial kit. Quantitative data are presented as mean ± SD (n = 3). Group differences were assessed by Student’s t-test, with ** indicating *P* < 0.01. Scale bar: 100 μm (B).

Supplementary Material 3. Full-length blots/gels are presented in Supplementary Material Original Western Blot Images.

## Data Availability

The data that support the findings of this study are available from the corresponding author upon reasonable request.

## Abbreviations

SPINK5: Kazal type 5
TCGA: The Cancer Genome Atla
KLK6: Kallikrein-related peptidase 6
LEKTI: Lymphoepithelial Kazal-type-related protease inhibitor
LSCC: Laryngeal squamous cell carcinoma
KLKs: Kallikrein-related peptidase family
HNSCC: Head and Neck Squamous Cell Carcinoma
TPM: Transcripts Per Million
GLUT1: Glucose Transporter 1
HK-II: Hexokinase II
2-DG: 2-deoxyglucose
