# Supplementary figures and images for: Downregulation of SPINK5 promotes laryngeal cancer cell malignancy through activation of KLK6-dependent glycolysis
Source: BMC Cancer. 2026 Apr 30;26:764. doi: 10.1186/s12885-026-15958-8 (PMC13285524; doi:10.1186/s12885-026-15958-8)

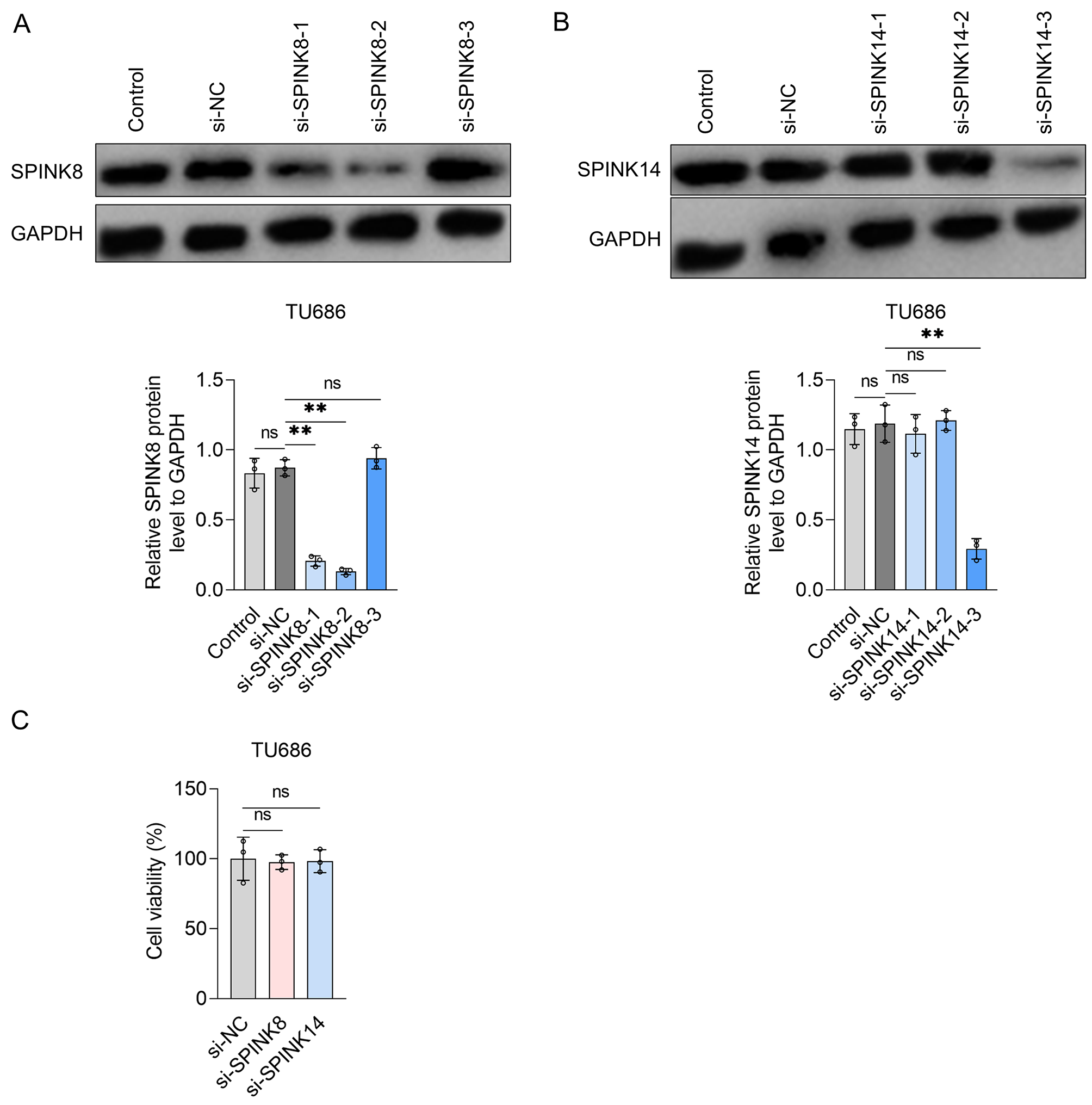

Supplement: Supplementary file 1 — Supplementary Material 1. Supplementary Figure 1. Effects of SPINK8 and SPINK14 on TU686 cell growth. (A-C) TU686 cells were transfected with SPINK5 siRNA or SPINK8 siRNA or SPINK14 siRNA for 72h. Then, (A) protein expression of SPINK8 and (B) SPINK14 was detected by Western blot analysis using GAPDH as the loading control, and (C) cell viability was measured by CCK-8 assay. Quantitative data are presented as mean ± SD (n = 3). Group differences were analyzed by one-way ANOVA, with **indicating P < 0.01 and ns indicating P > 0.05. [file 12885_2026_15958_MOESM1_ESM.tif]

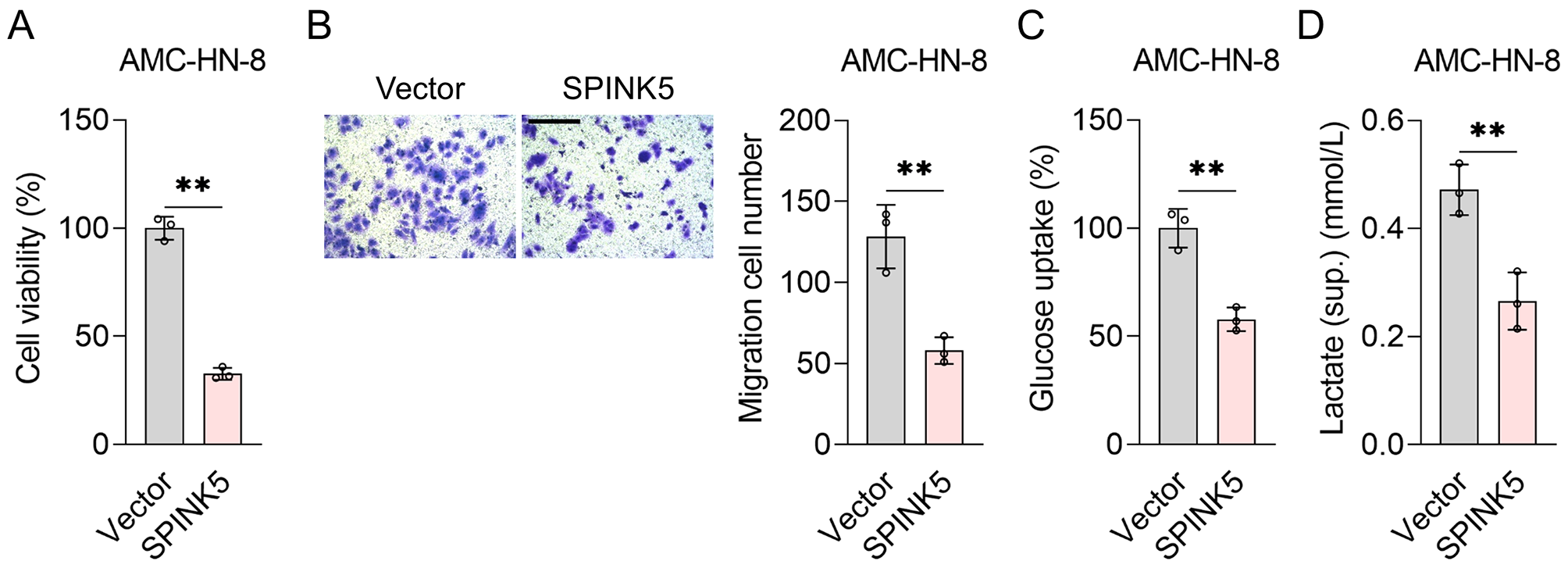

Supplement: Supplementary file 2 — Supplementary Material 2. Supplementary Figure 2. Effects of SPINK5 on glycolysis, growth and migration of AMC-HN-8 cells. (A-D) AMC-HN-8 cells were transfected with SPINK5 plasmid for 72h. Then, (A) cell viability was measured by CCK-8 assay, (B) cell migration was evaluated by transwell assay, (C) glucose uptake was measured by 2-NBDG assay, and (D) lactate levels in cell supernatants were detected using a commercial kit. Quantitative data are presented as mean ± SD (n = 3). Group differences were assessed by Student’s t-test, with ** indicating P < 0.01. Scale bar: 100 μm (B). [file 12885_2026_15958_MOESM2_ESM.tif]

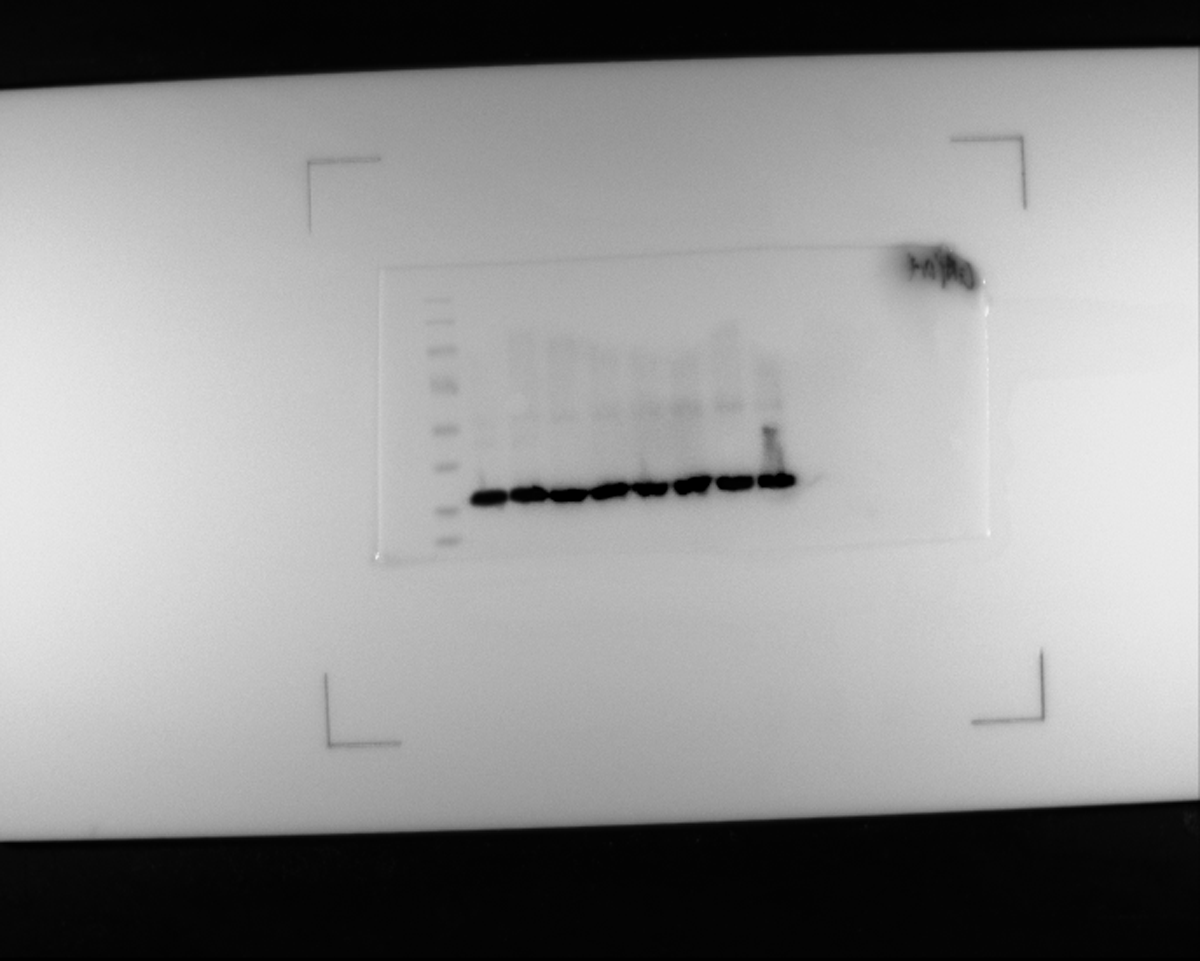

Supplement: Supplementary file 3 — Supplementary Material 3. Full-length blots/gels are presented in Supplementary Material Original Western Blot Images. [file 12885_2026_15958_MOESM3_ESM.zip › Supplementary Material Original Western Blot Images/Fig2 WB/Fig2A.GAPDH.tif]

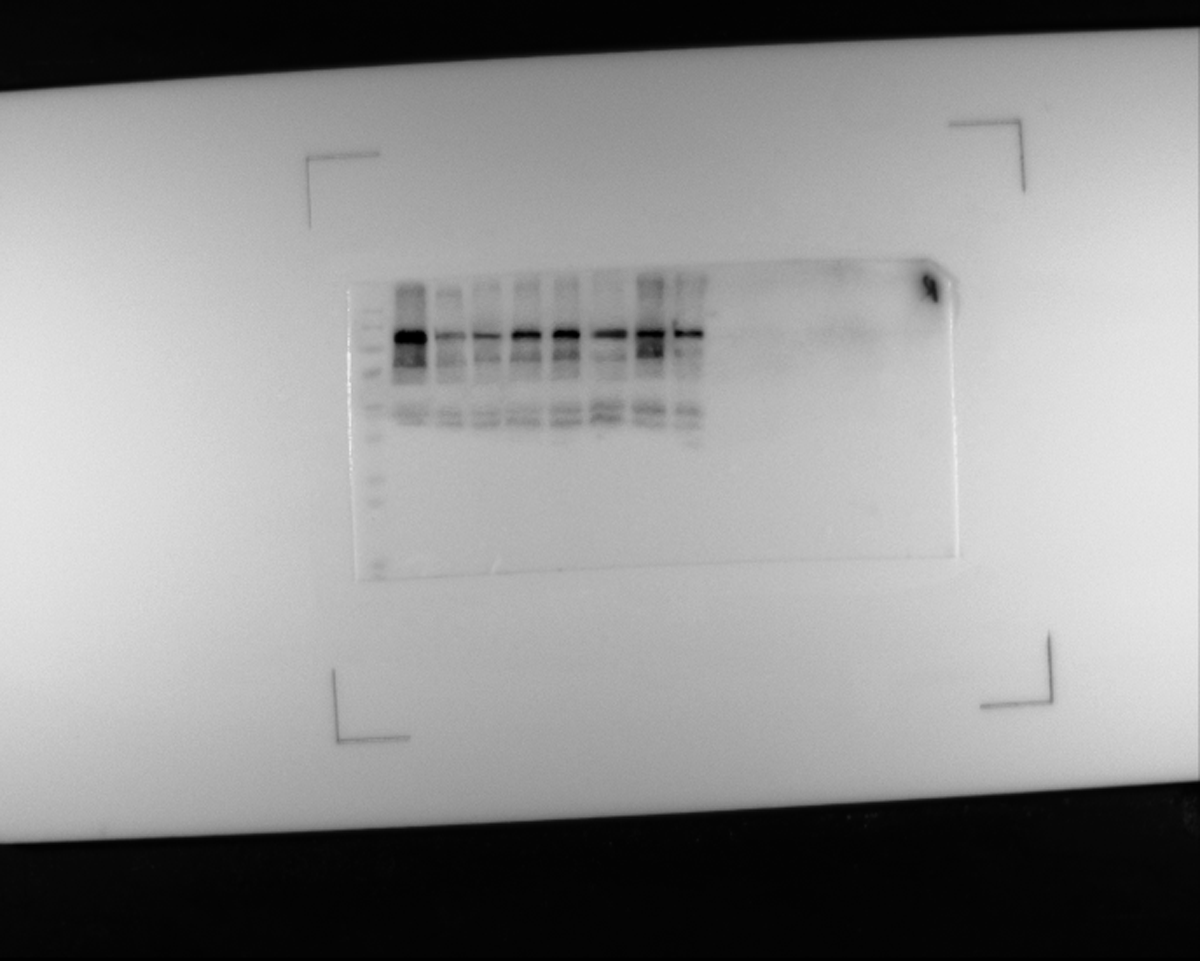

Supplement: Supplementary file 3 — Supplementary Material 3. Full-length blots/gels are presented in Supplementary Material Original Western Blot Images. [file 12885_2026_15958_MOESM3_ESM.zip › Supplementary Material Original Western Blot Images/Fig2 WB/Fig2A.SPINK5.tif]

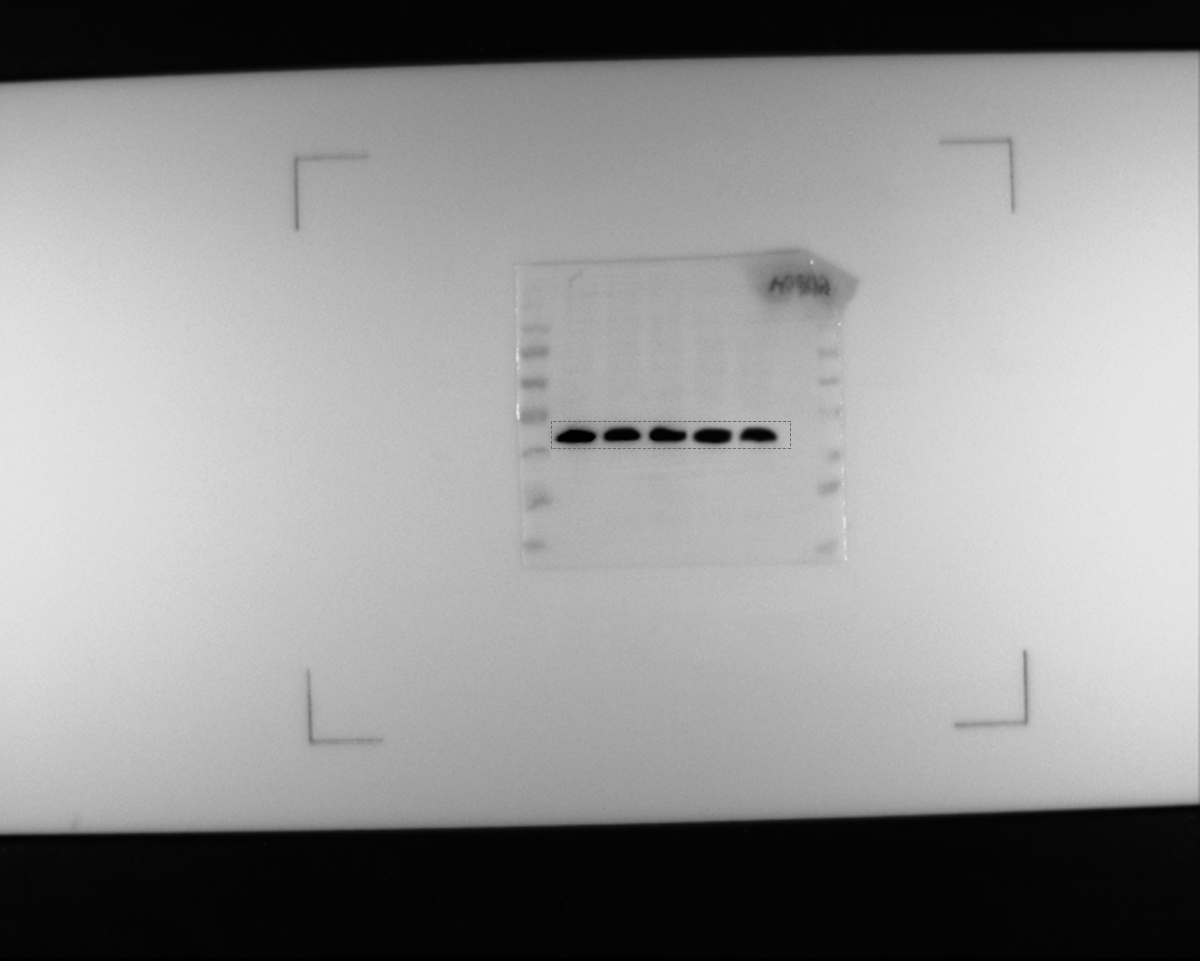

Supplement: Supplementary file 3 — Supplementary Material 3. Full-length blots/gels are presented in Supplementary Material Original Western Blot Images. [file 12885_2026_15958_MOESM3_ESM.zip › Supplementary Material Original Western Blot Images/Fig2 WB/Fig2B.GAPDH.tif]

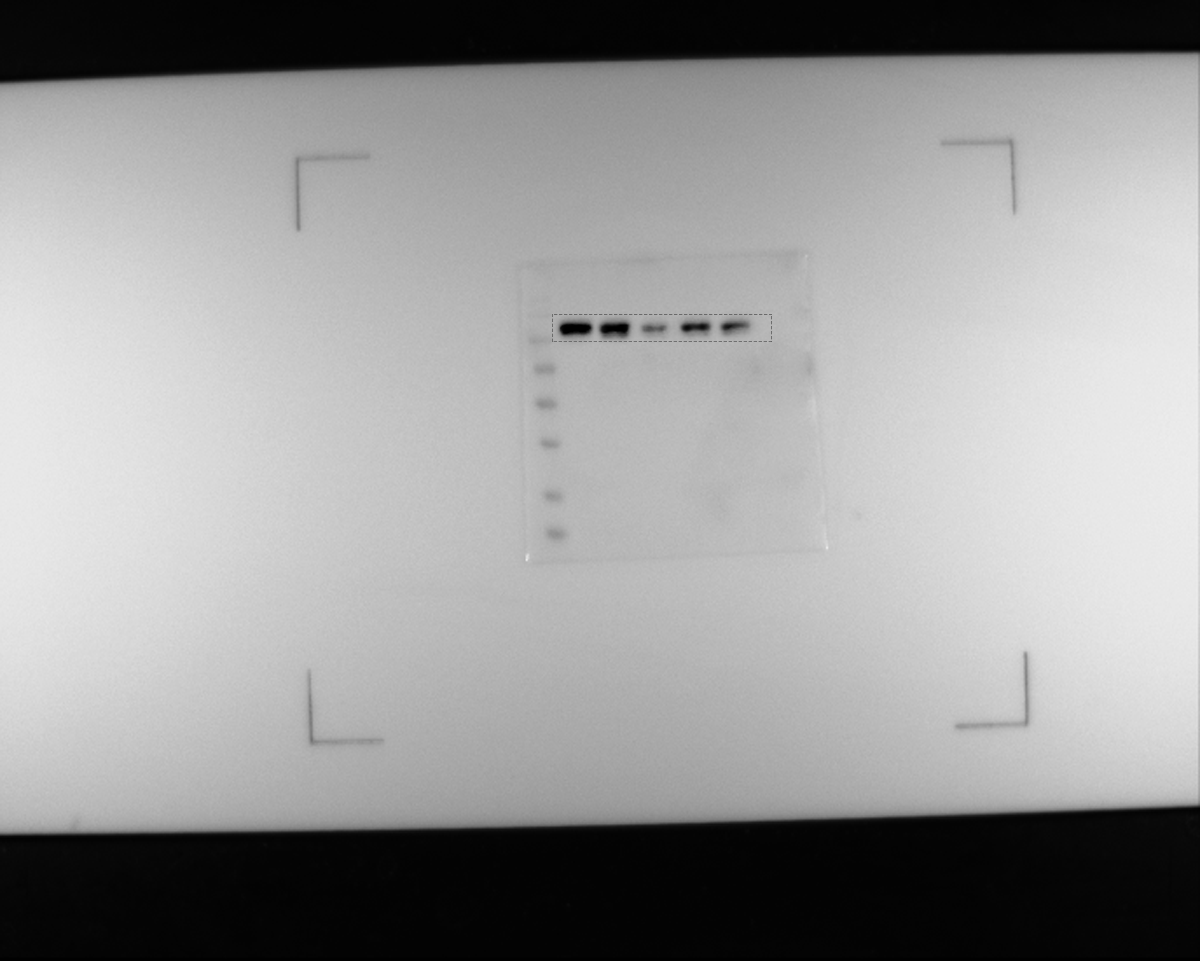

Supplement: Supplementary file 3 — Supplementary Material 3. Full-length blots/gels are presented in Supplementary Material Original Western Blot Images. [file 12885_2026_15958_MOESM3_ESM.zip › Supplementary Material Original Western Blot Images/Fig2 WB/Fig2B.SPINK5.tif]

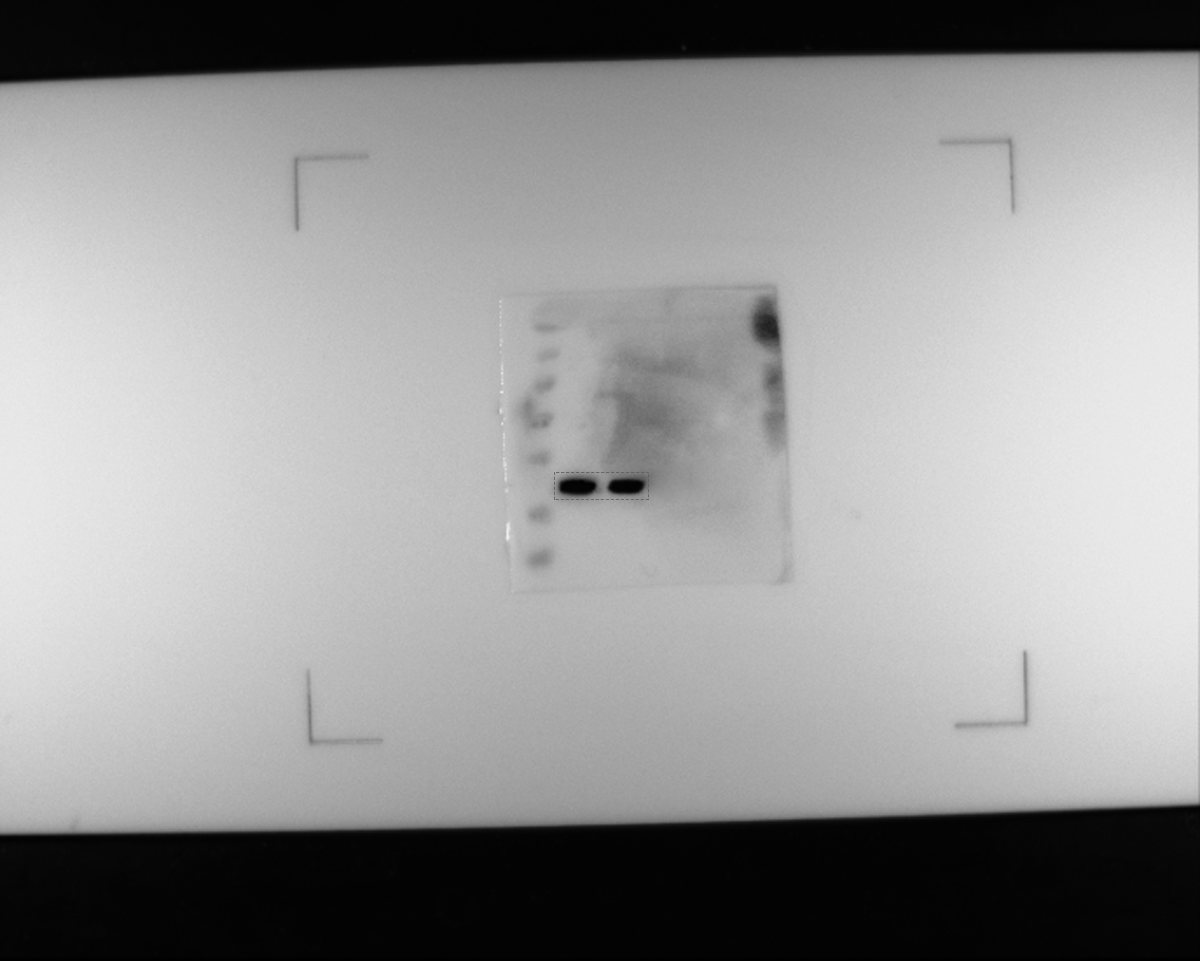

Supplement: Supplementary file 3 — Supplementary Material 3. Full-length blots/gels are presented in Supplementary Material Original Western Blot Images. [file 12885_2026_15958_MOESM3_ESM.zip › Supplementary Material Original Western Blot Images/Fig2 WB/Fig2F.GAPDH.tif]

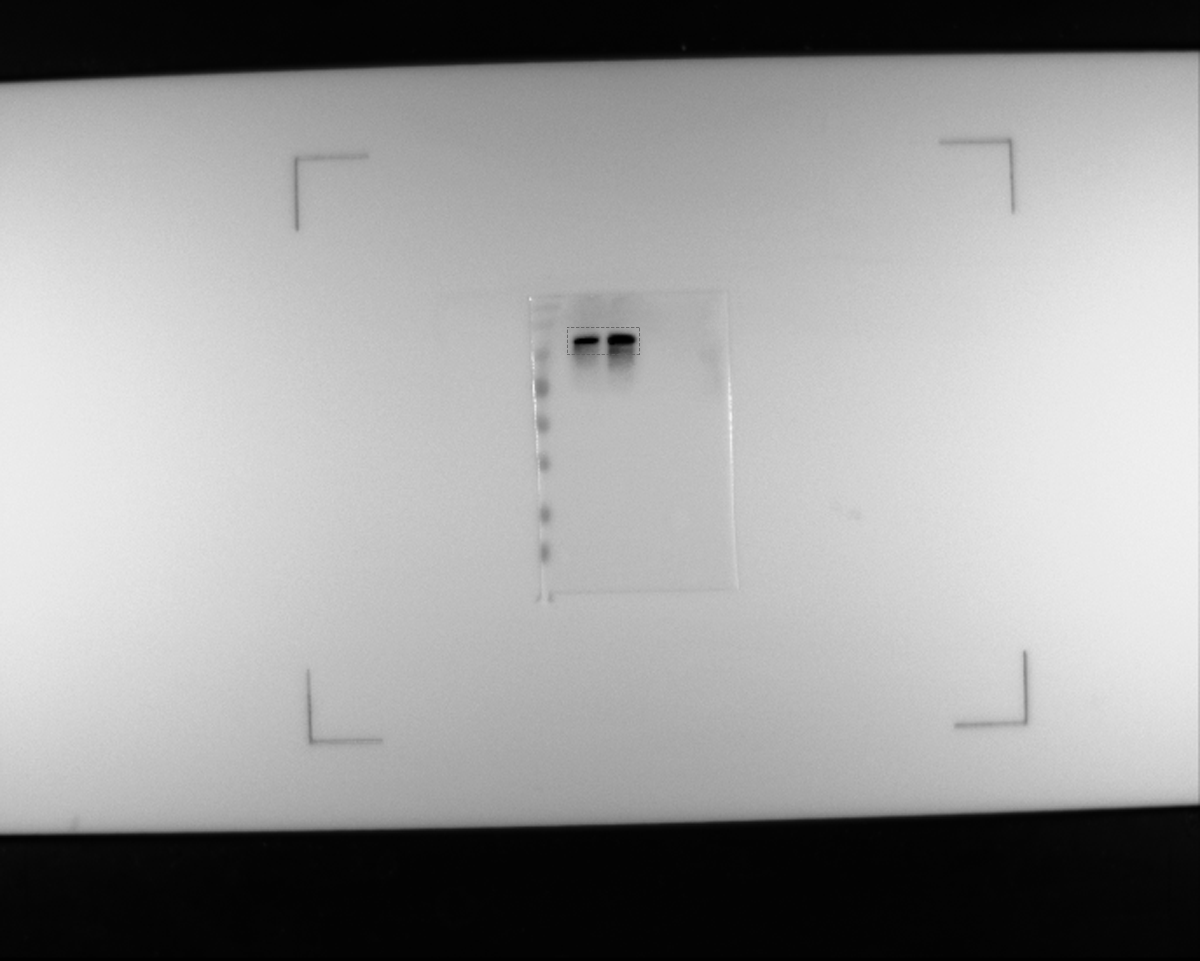

Supplement: Supplementary file 3 — Supplementary Material 3. Full-length blots/gels are presented in Supplementary Material Original Western Blot Images. [file 12885_2026_15958_MOESM3_ESM.zip › Supplementary Material Original Western Blot Images/Fig2 WB/Fig2F.SPINK5.tif]

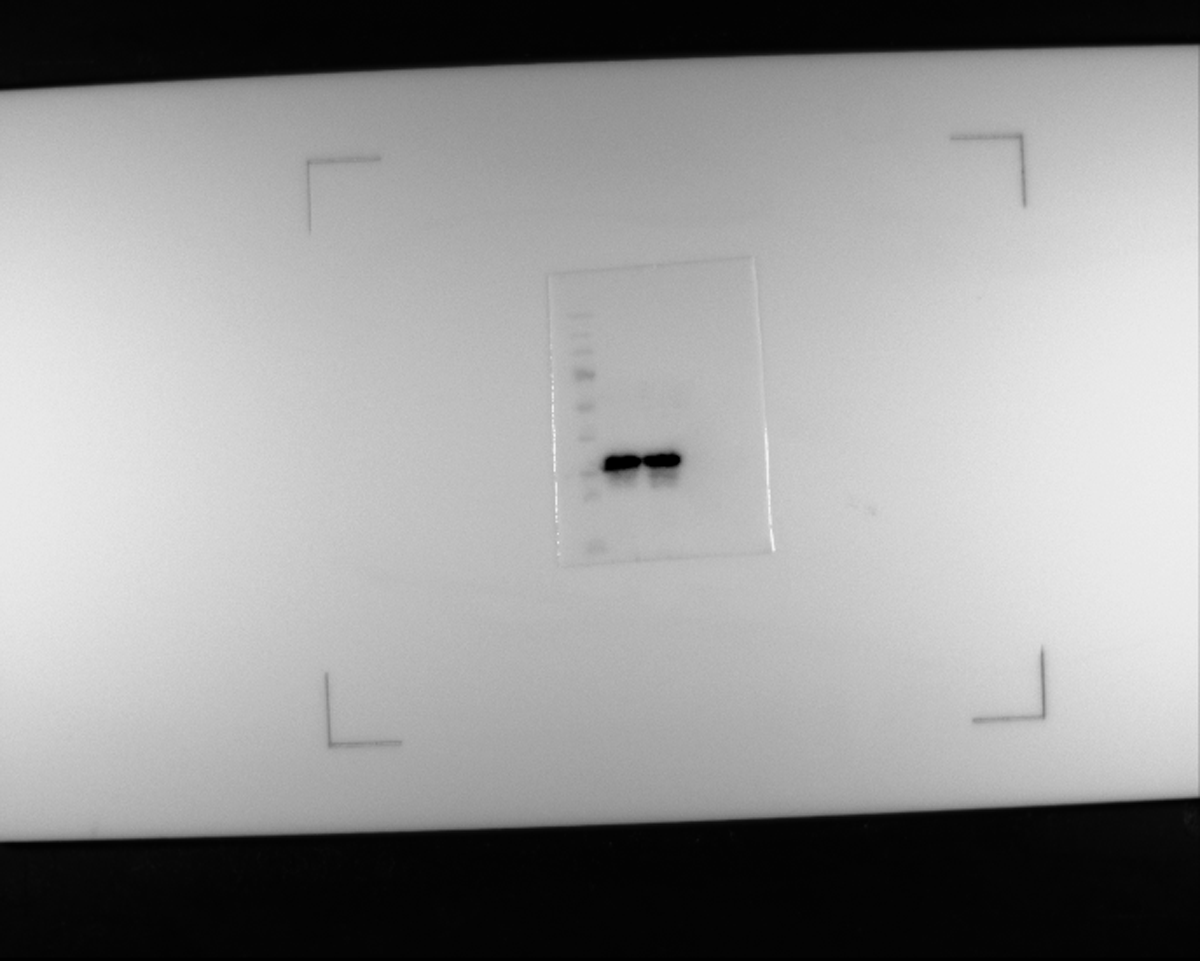

Supplement: Supplementary file 3 — Supplementary Material 3. Full-length blots/gels are presented in Supplementary Material Original Western Blot Images. [file 12885_2026_15958_MOESM3_ESM.zip › Supplementary Material Original Western Blot Images/Fig2 WB/Fig2J.GAPDH.tif]

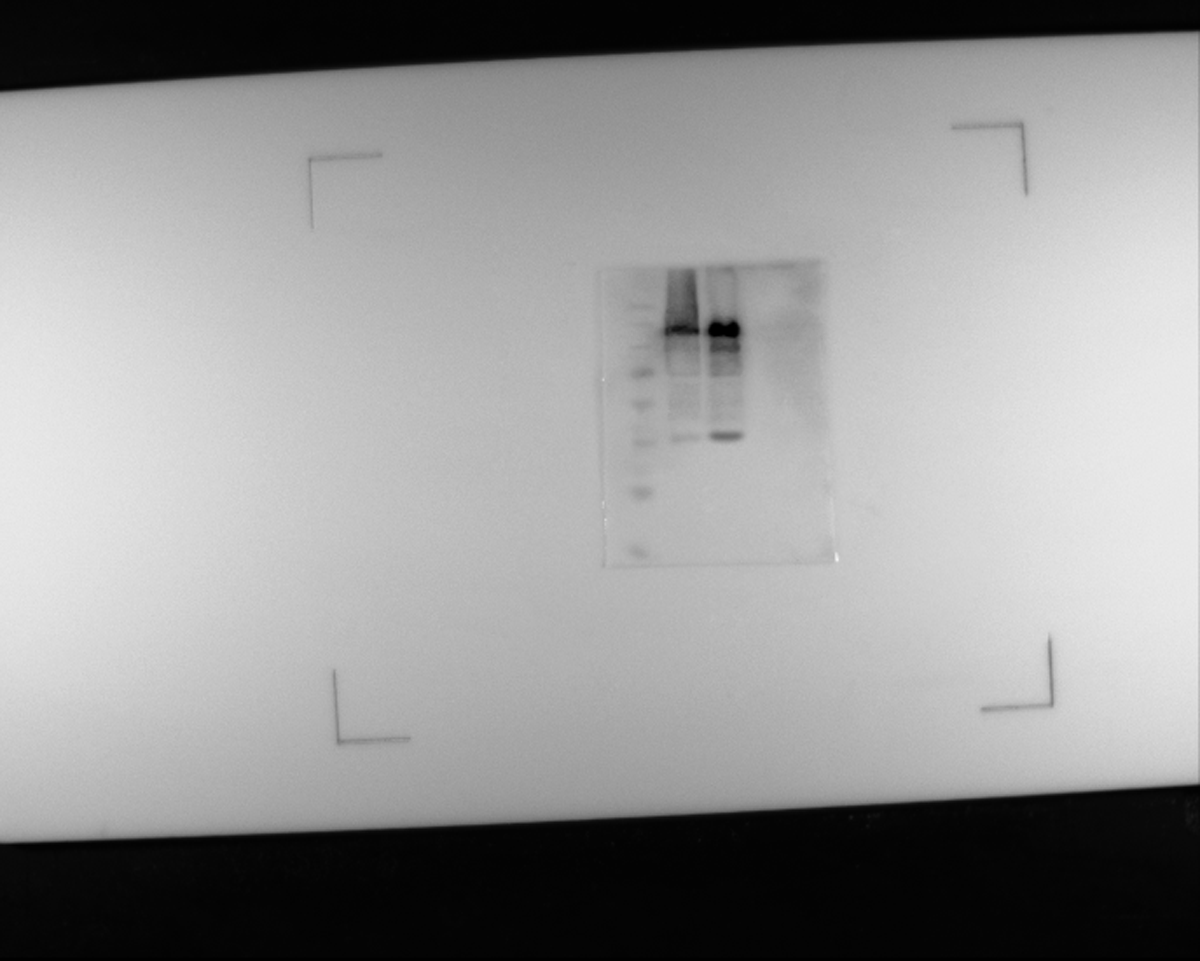

Supplement: Supplementary file 3 — Supplementary Material 3. Full-length blots/gels are presented in Supplementary Material Original Western Blot Images. [file 12885_2026_15958_MOESM3_ESM.zip › Supplementary Material Original Western Blot Images/Fig2 WB/Fig2J.SPINK5.tif]

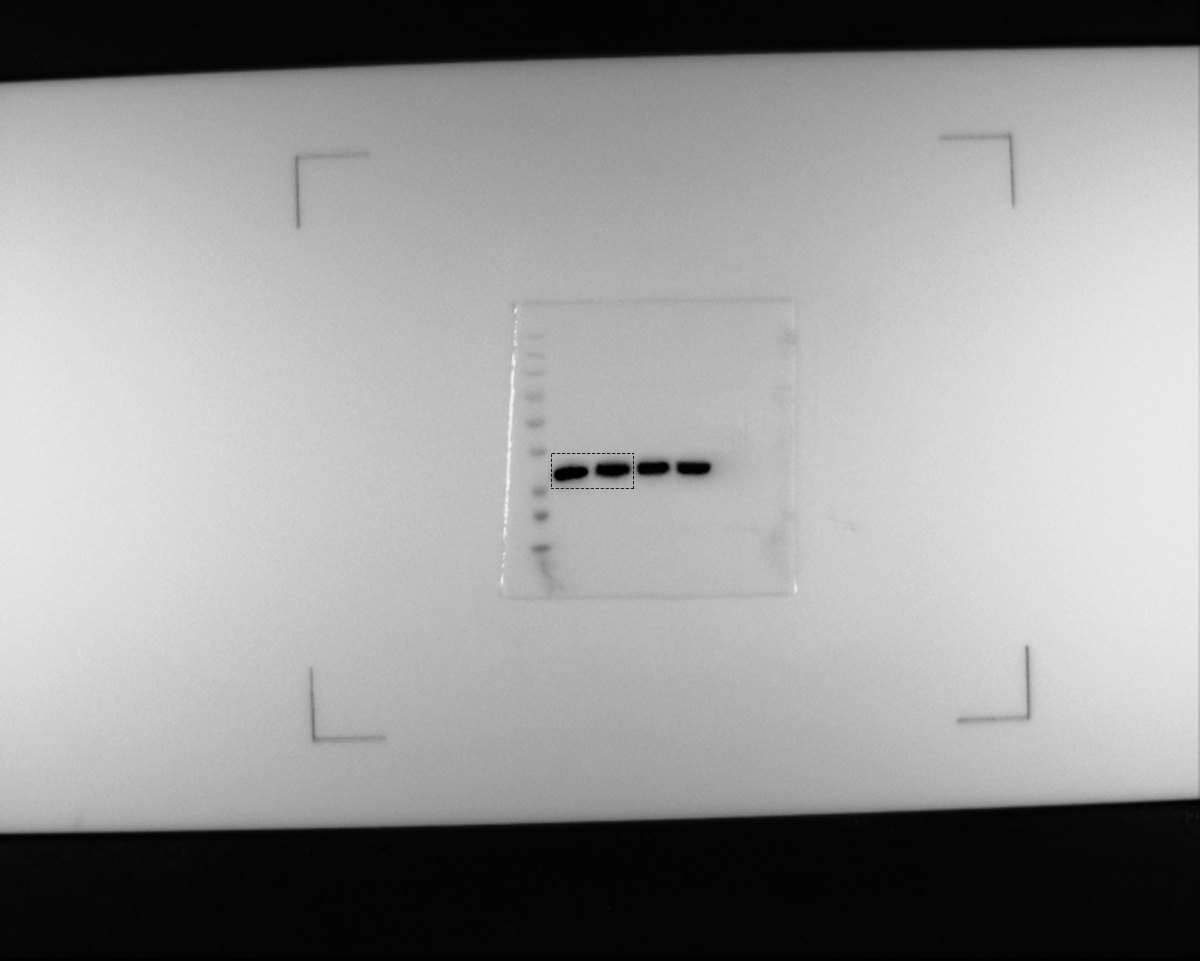

Supplement: Supplementary file 3 — Supplementary Material 3. Full-length blots/gels are presented in Supplementary Material Original Western Blot Images. [file 12885_2026_15958_MOESM3_ESM.zip › Supplementary Material Original Western Blot Images/Fig3 WB/Fig3A.GAPDH.tif]

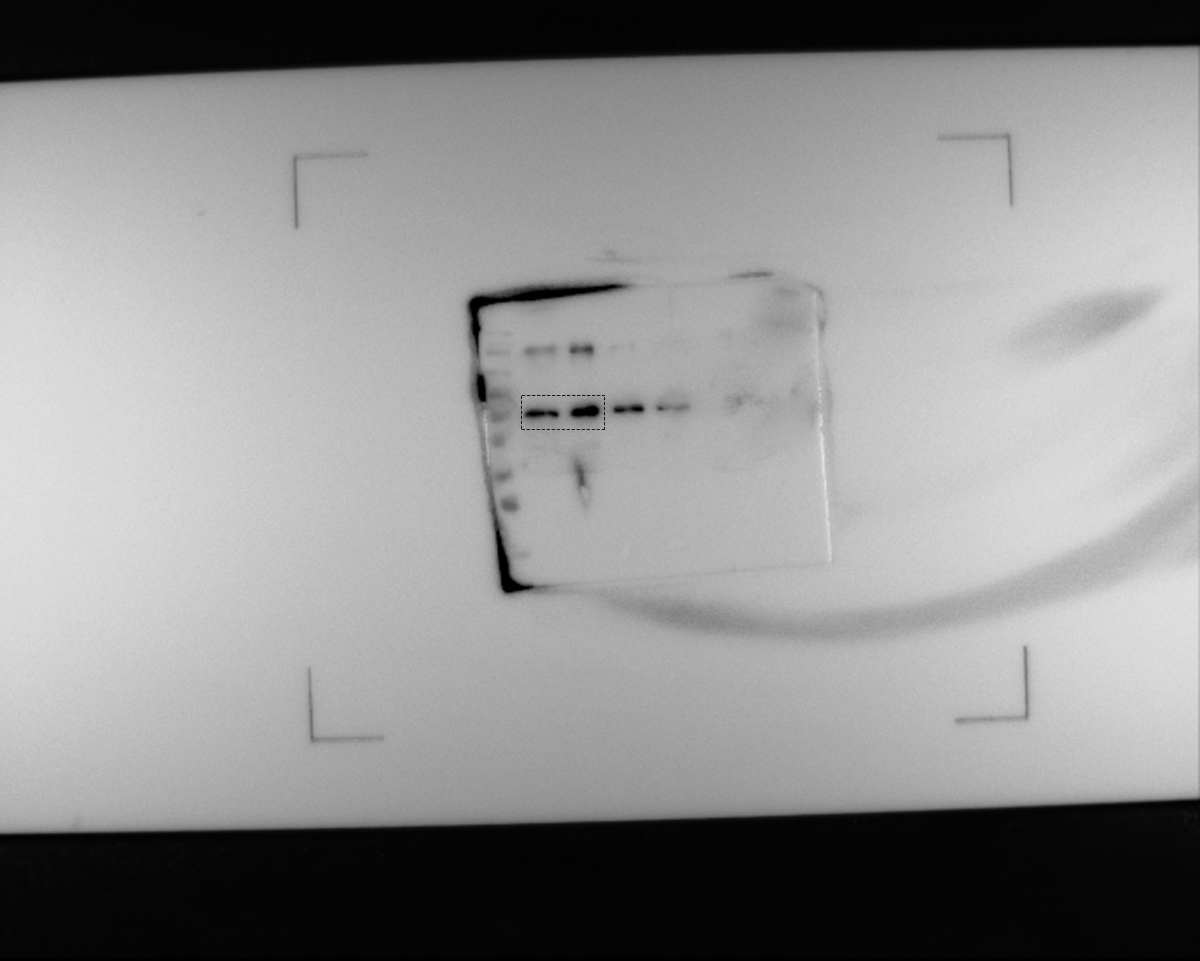

Supplement: Supplementary file 3 — Supplementary Material 3. Full-length blots/gels are presented in Supplementary Material Original Western Blot Images. [file 12885_2026_15958_MOESM3_ESM.zip › Supplementary Material Original Western Blot Images/Fig3 WB/Fig3A.GLUT1.tif]

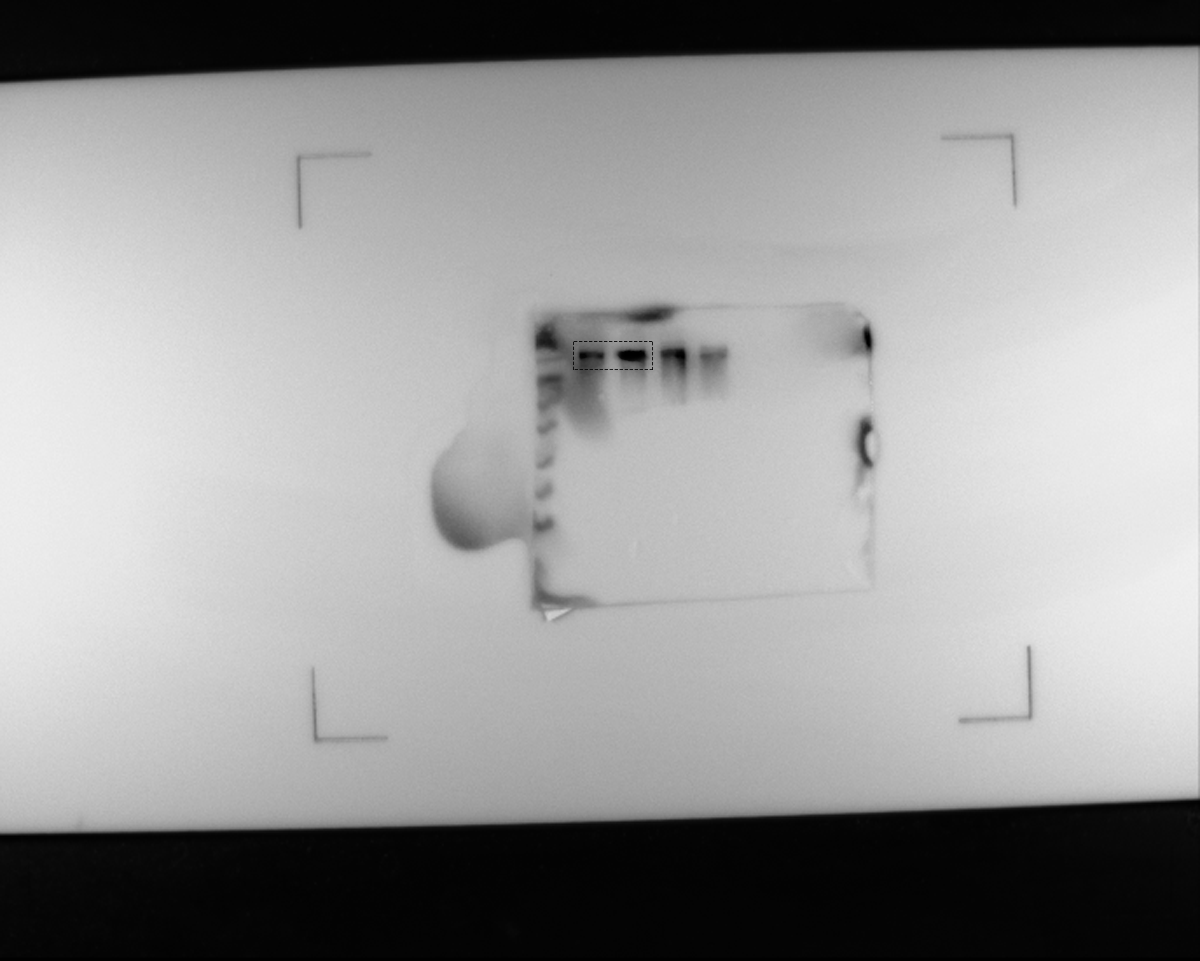

Supplement: Supplementary file 3 — Supplementary Material 3. Full-length blots/gels are presented in Supplementary Material Original Western Blot Images. [file 12885_2026_15958_MOESM3_ESM.zip › Supplementary Material Original Western Blot Images/Fig3 WB/Fig3A.HK-II.tif]

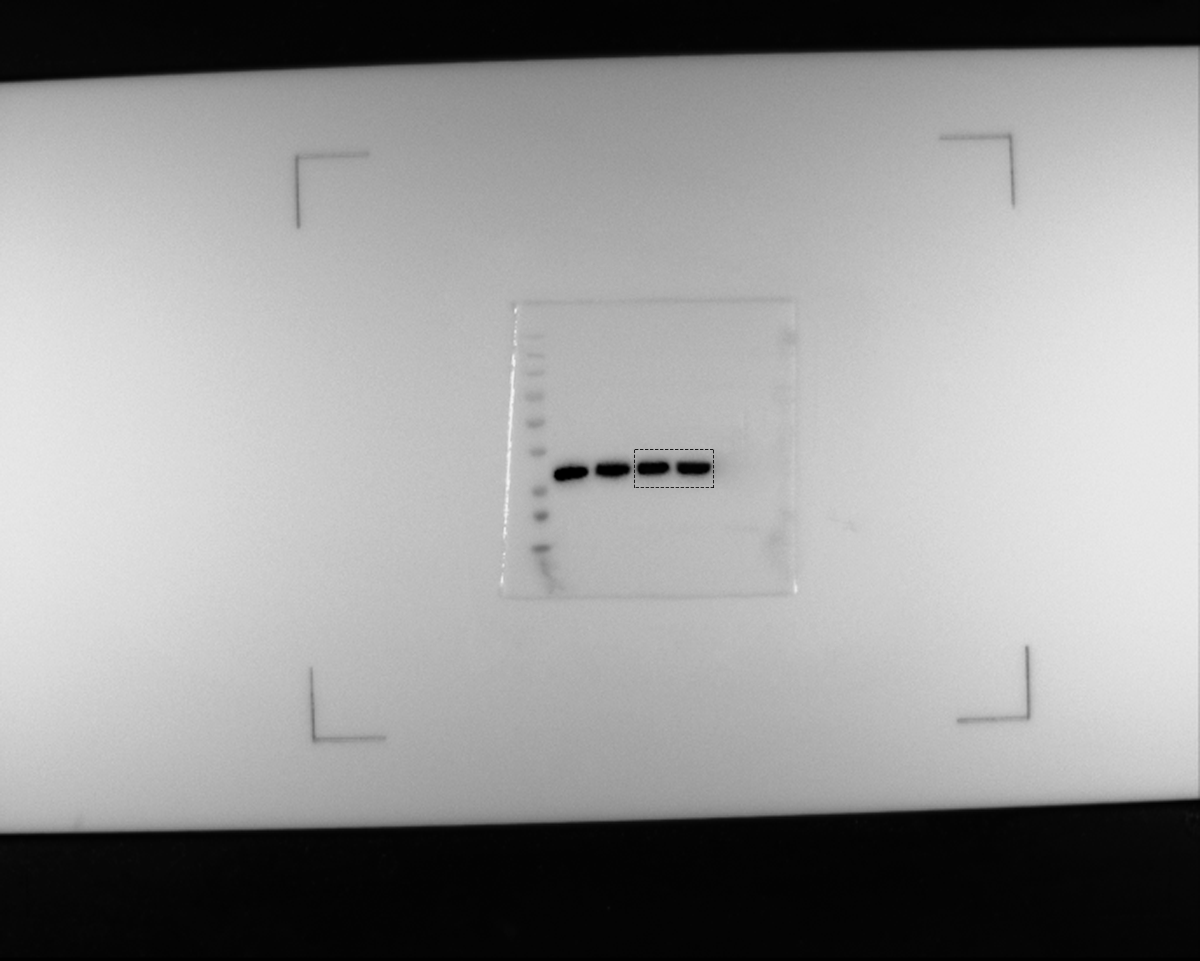

Supplement: Supplementary file 3 — Supplementary Material 3. Full-length blots/gels are presented in Supplementary Material Original Western Blot Images. [file 12885_2026_15958_MOESM3_ESM.zip › Supplementary Material Original Western Blot Images/Fig3 WB/Fig3D.GAPDH.tif]

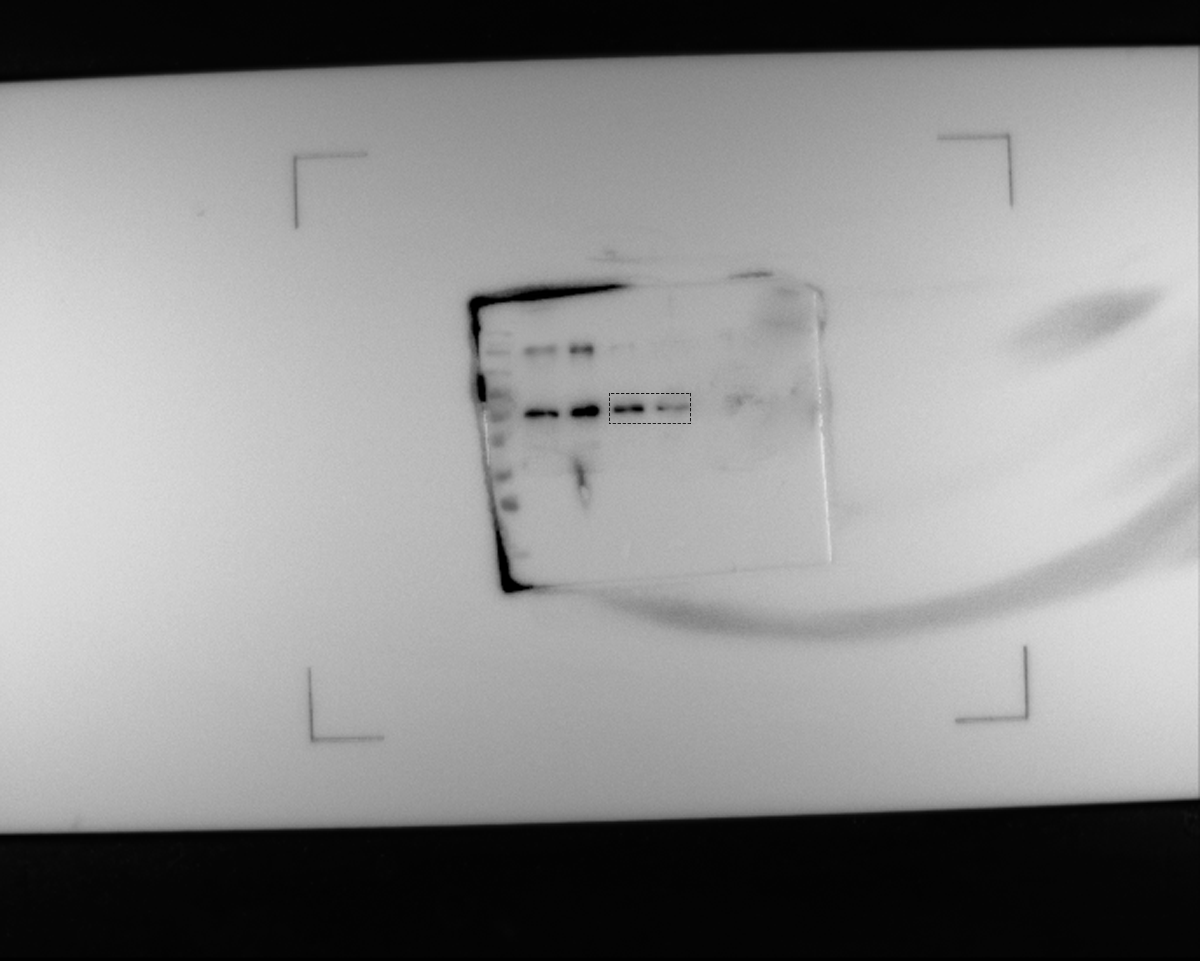

Supplement: Supplementary file 3 — Supplementary Material 3. Full-length blots/gels are presented in Supplementary Material Original Western Blot Images. [file 12885_2026_15958_MOESM3_ESM.zip › Supplementary Material Original Western Blot Images/Fig3 WB/Fig3D.GLUT1.tif]

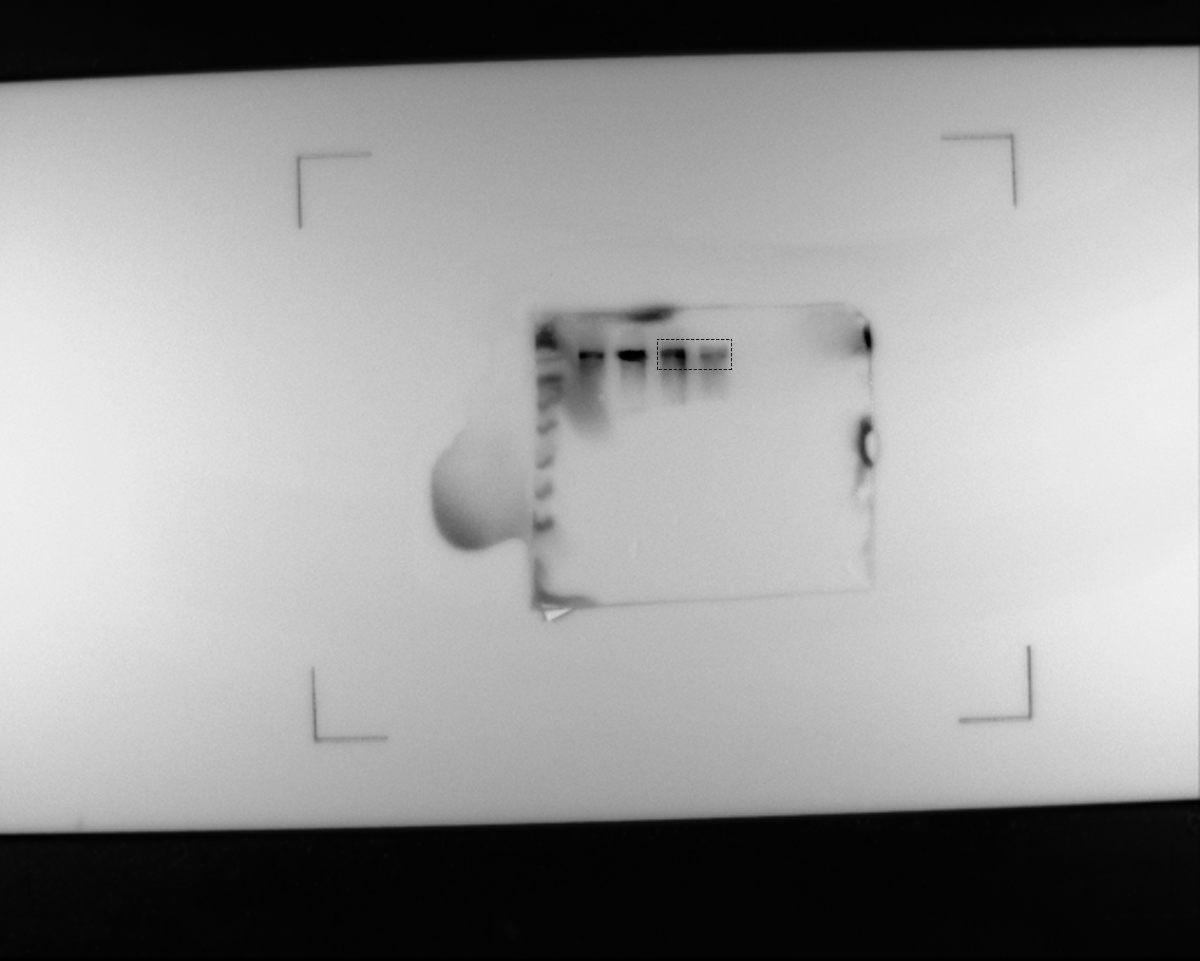

Supplement: Supplementary file 3 — Supplementary Material 3. Full-length blots/gels are presented in Supplementary Material Original Western Blot Images. [file 12885_2026_15958_MOESM3_ESM.zip › Supplementary Material Original Western Blot Images/Fig3 WB/Fig3D.HK-II.tif]

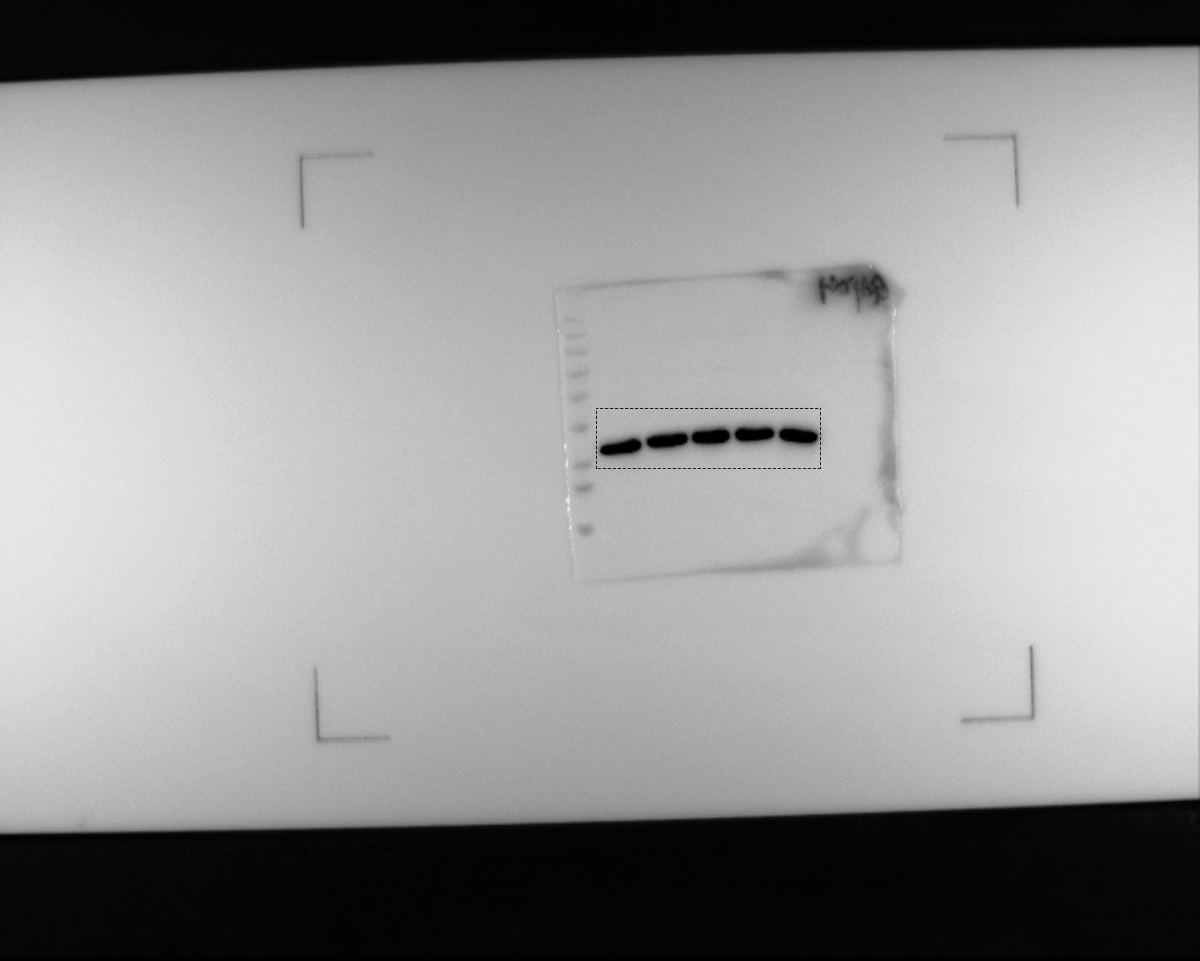

Supplement: Supplementary file 3 — Supplementary Material 3. Full-length blots/gels are presented in Supplementary Material Original Western Blot Images. [file 12885_2026_15958_MOESM3_ESM.zip › Supplementary Material Original Western Blot Images/Fig5 WB/Fig5A.GAPDH.tif]

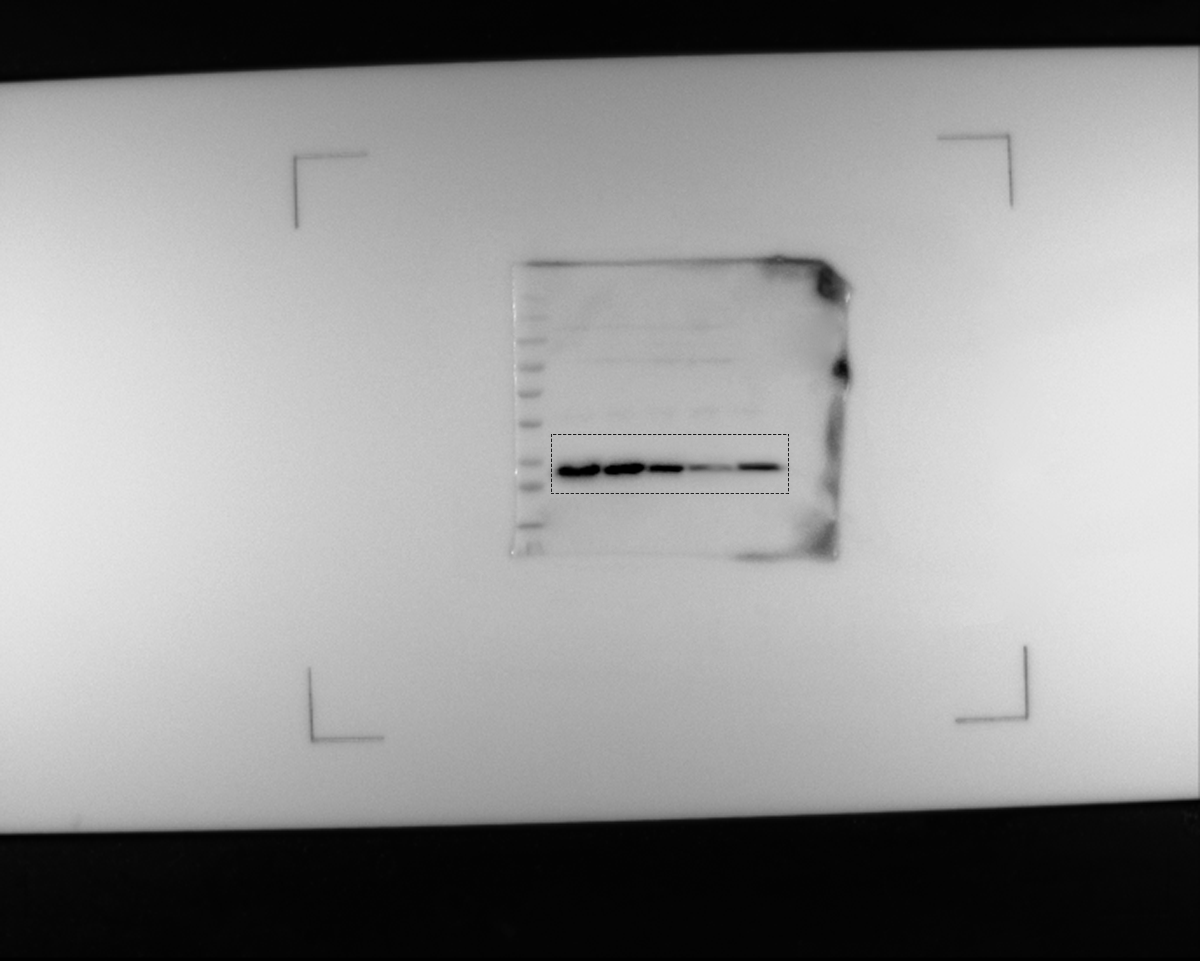

Supplement: Supplementary file 3 — Supplementary Material 3. Full-length blots/gels are presented in Supplementary Material Original Western Blot Images. [file 12885_2026_15958_MOESM3_ESM.zip › Supplementary Material Original Western Blot Images/Fig5 WB/Fig5A.KLK6.tif]

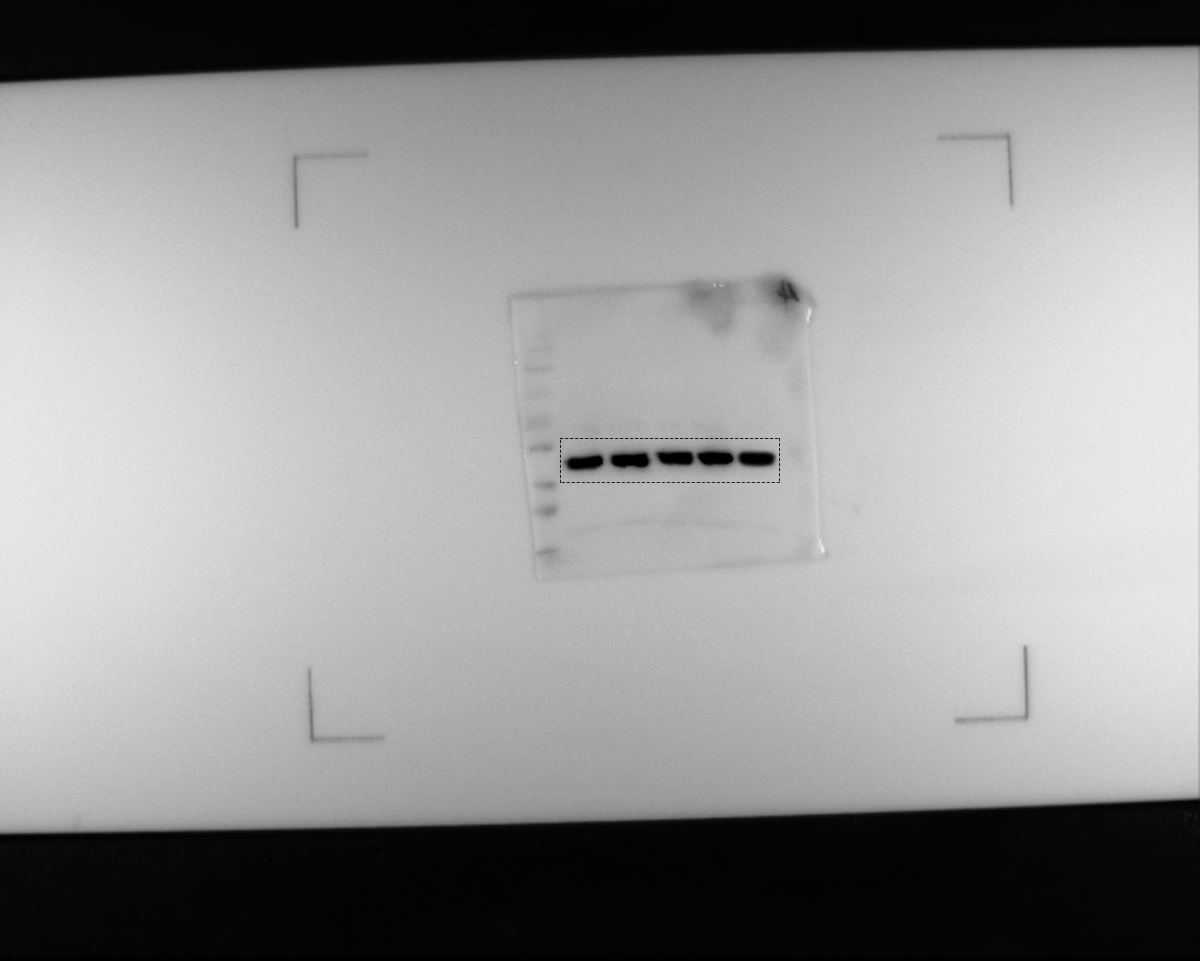

Supplement: Supplementary file 3 — Supplementary Material 3. Full-length blots/gels are presented in Supplementary Material Original Western Blot Images. [file 12885_2026_15958_MOESM3_ESM.zip › Supplementary Material Original Western Blot Images/Fig5 WB/Fig5B.GAPDH.tif]

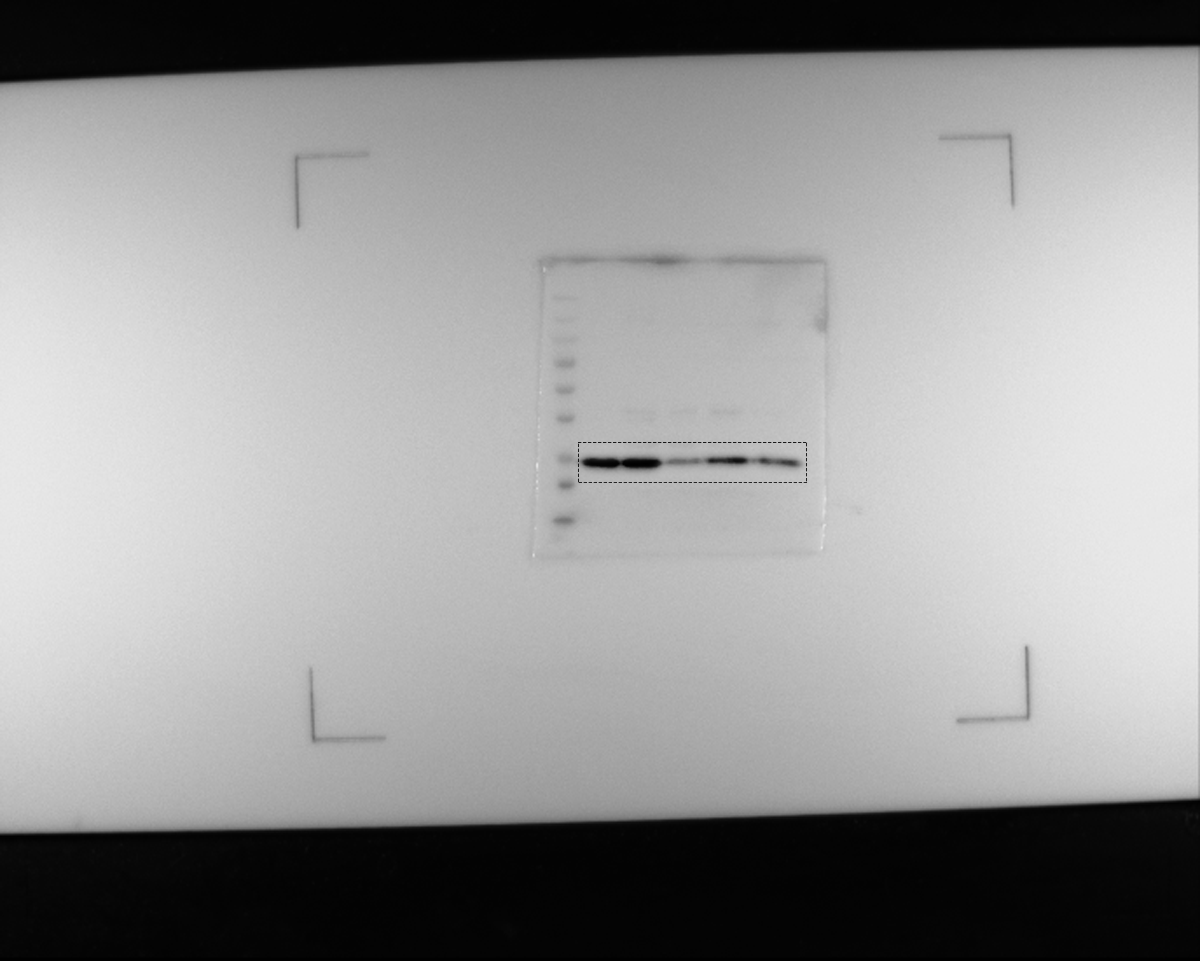

Supplement: Supplementary file 3 — Supplementary Material 3. Full-length blots/gels are presented in Supplementary Material Original Western Blot Images. [file 12885_2026_15958_MOESM3_ESM.zip › Supplementary Material Original Western Blot Images/Fig5 WB/Fig5B.KLK10.tif]

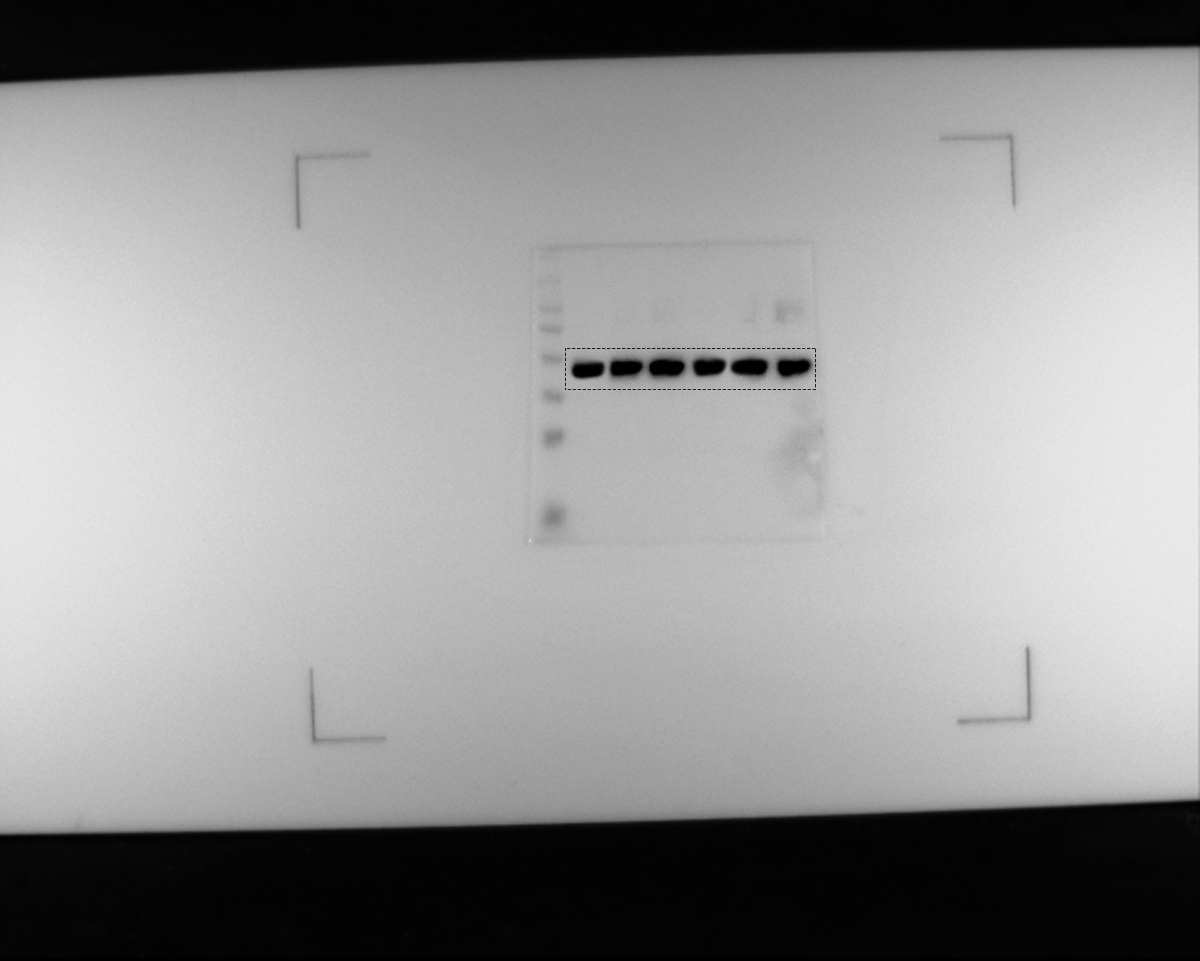

Supplement: Supplementary file 3 — Supplementary Material 3. Full-length blots/gels are presented in Supplementary Material Original Western Blot Images. [file 12885_2026_15958_MOESM3_ESM.zip › Supplementary Material Original Western Blot Images/Fig5 WB/Fig5C.GAPDH.tif]

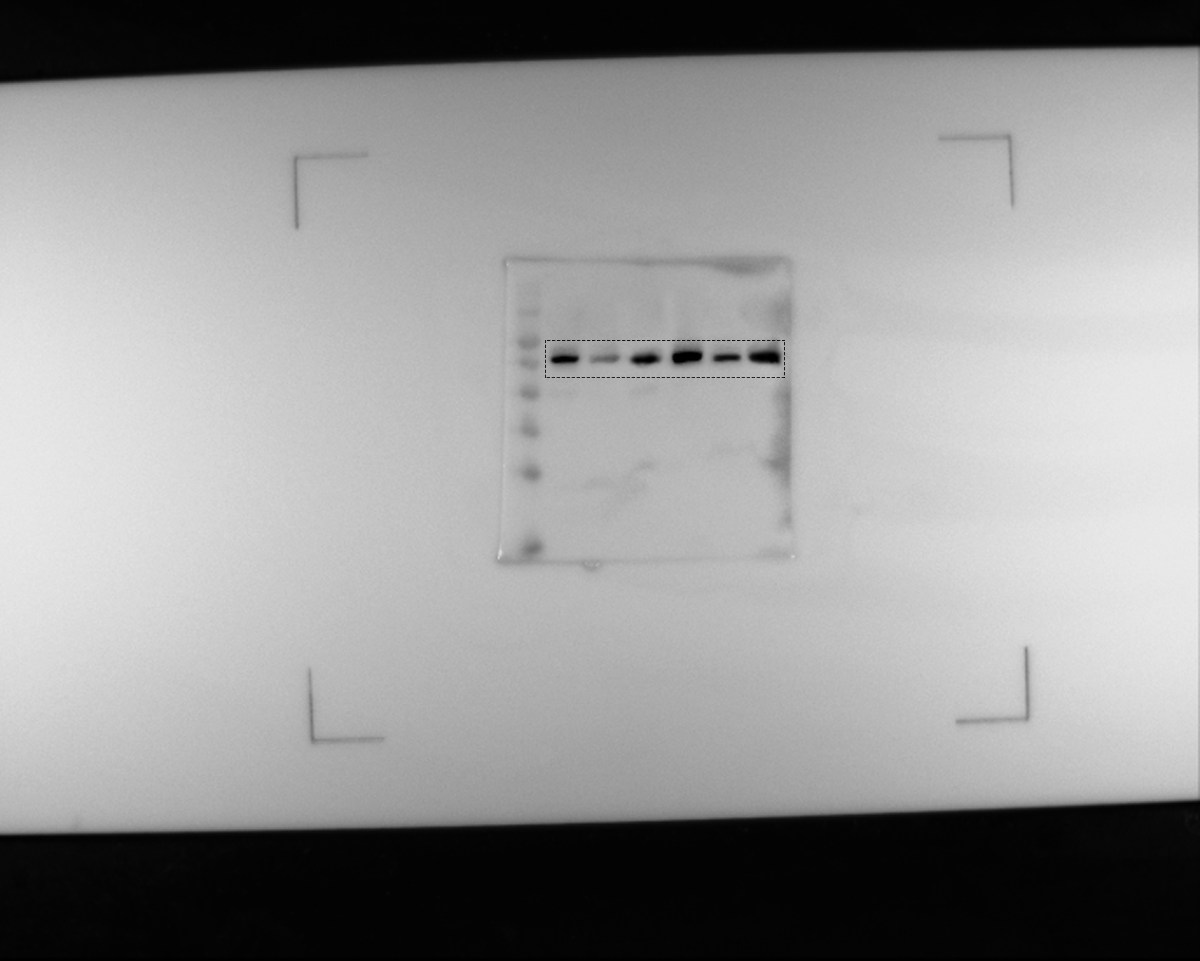

Supplement: Supplementary file 3 — Supplementary Material 3. Full-length blots/gels are presented in Supplementary Material Original Western Blot Images. [file 12885_2026_15958_MOESM3_ESM.zip › Supplementary Material Original Western Blot Images/Fig5 WB/Fig5C.GLUT1.tif]

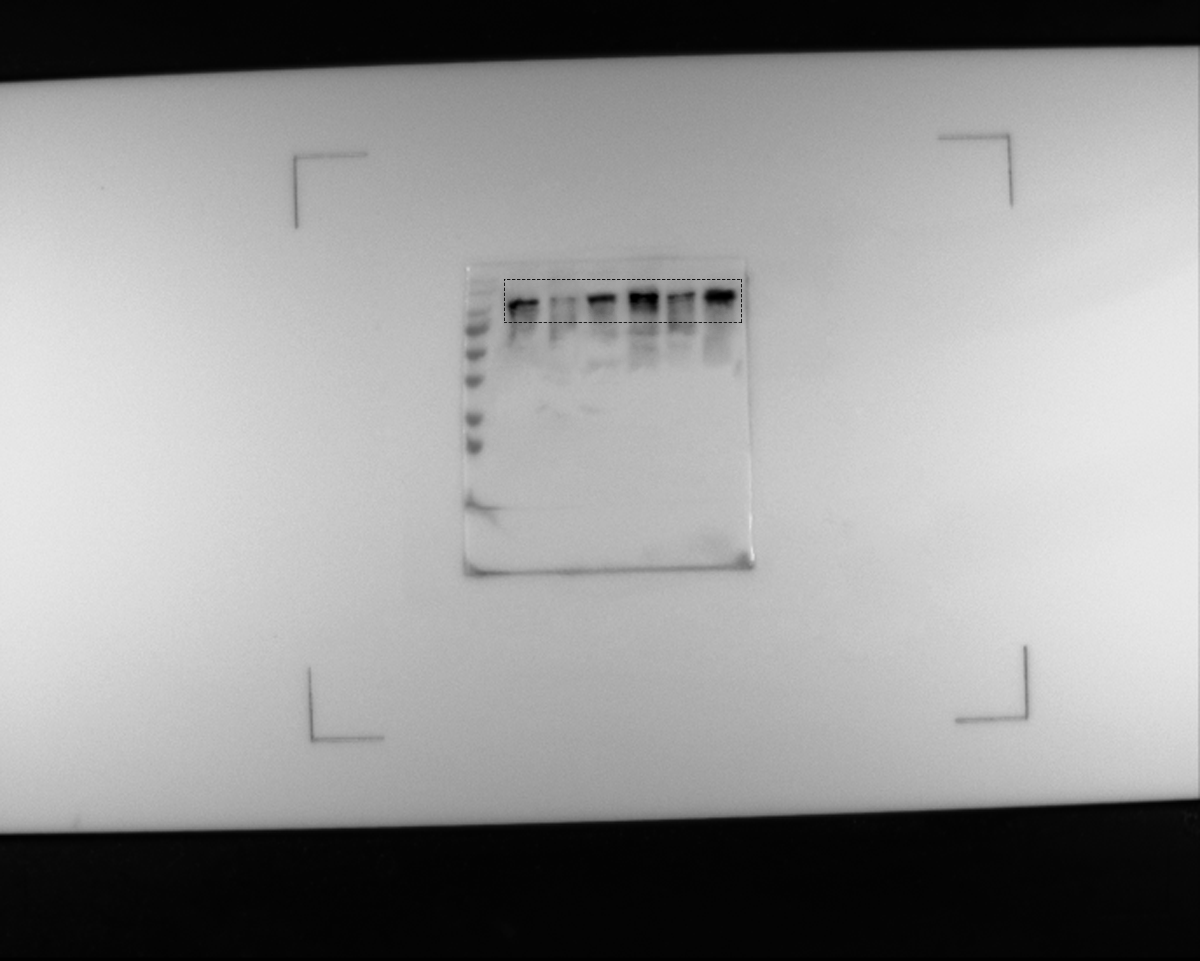

Supplement: Supplementary file 3 — Supplementary Material 3. Full-length blots/gels are presented in Supplementary Material Original Western Blot Images. [file 12885_2026_15958_MOESM3_ESM.zip › Supplementary Material Original Western Blot Images/Fig5 WB/Fig5C.HK-II.tif]

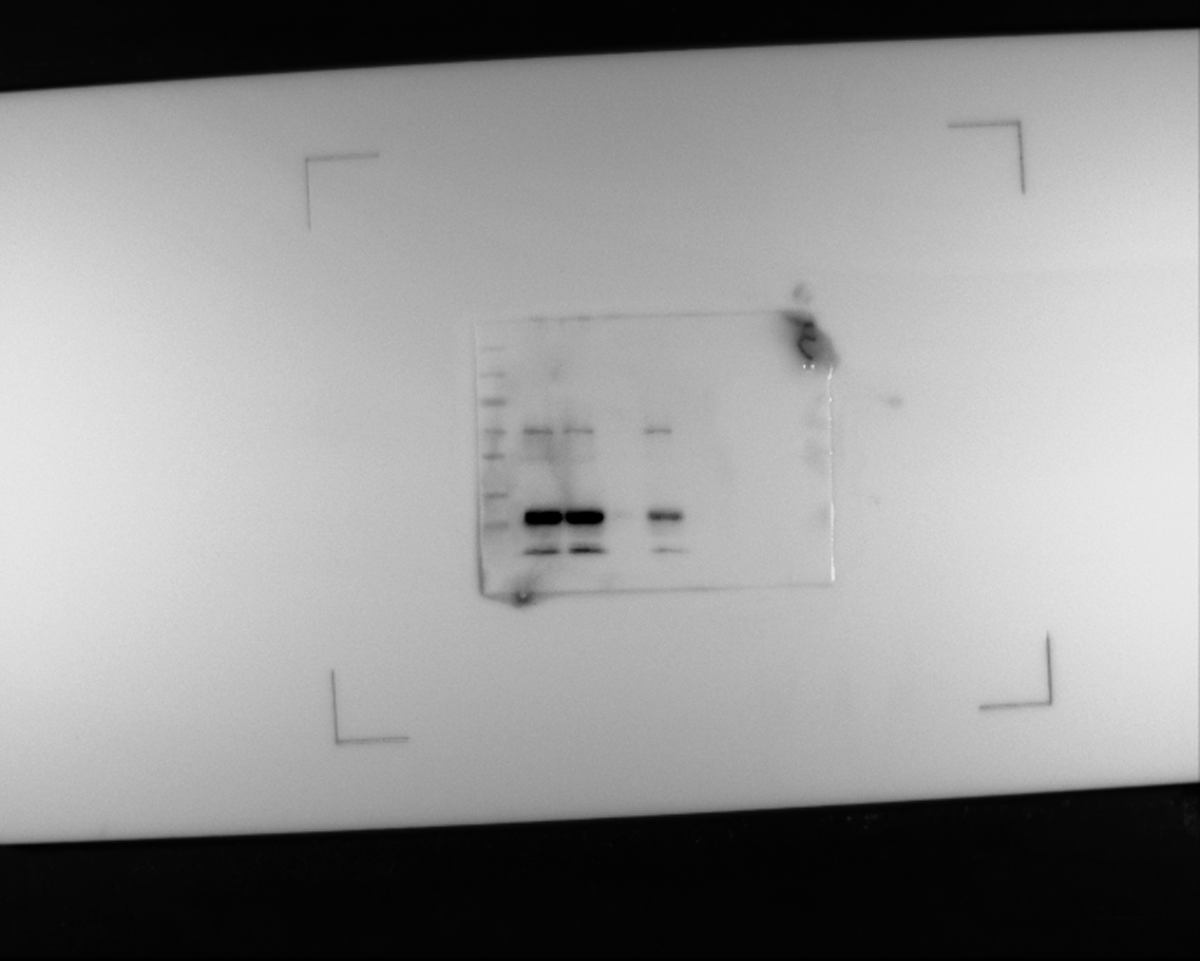

Supplement: Supplementary file 3 — Supplementary Material 3. Full-length blots/gels are presented in Supplementary Material Original Western Blot Images. [file 12885_2026_15958_MOESM3_ESM.zip › Supplementary Material Original Western Blot Images/Fig5 WB/Fig5G.KLK6.tif]

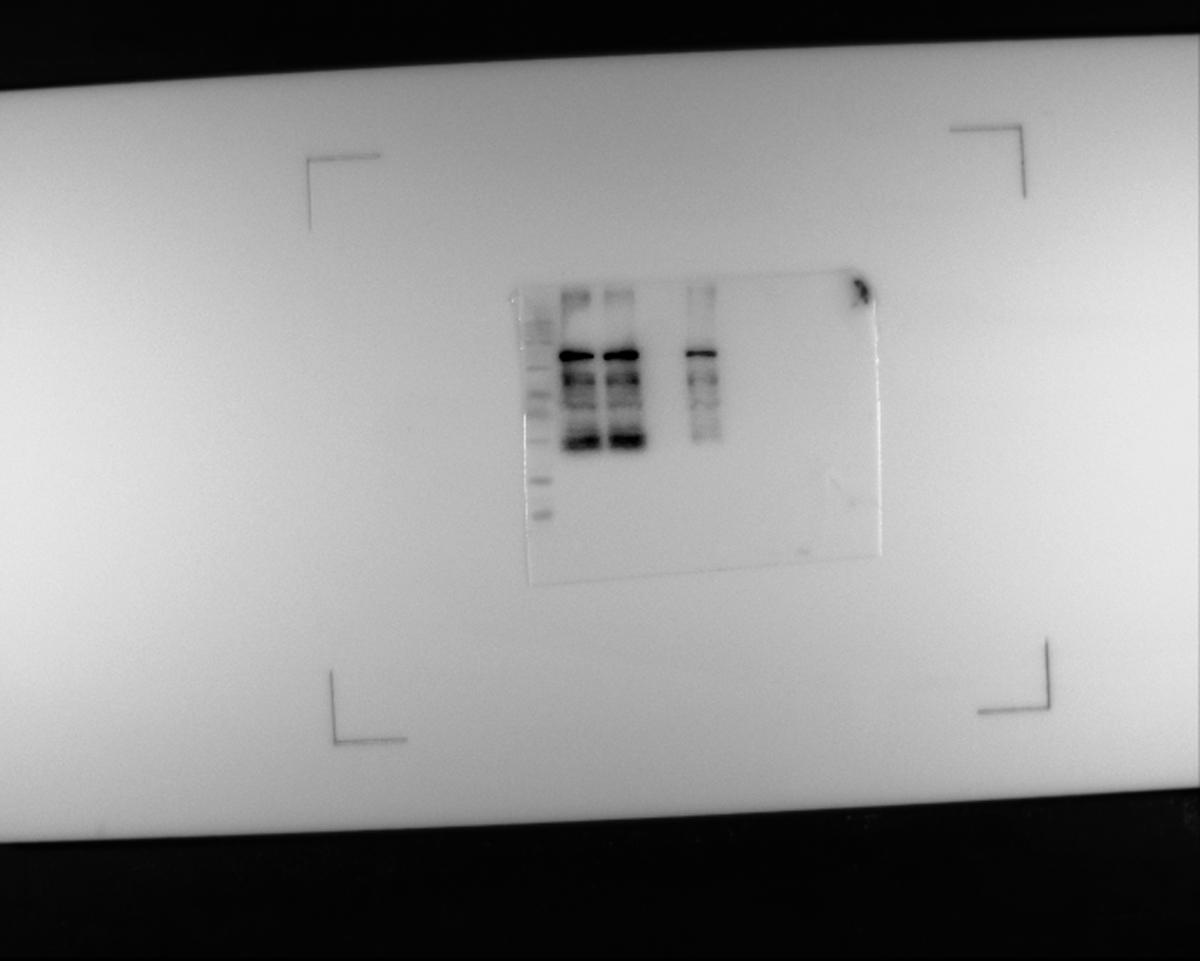

Supplement: Supplementary file 3 — Supplementary Material 3. Full-length blots/gels are presented in Supplementary Material Original Western Blot Images. [file 12885_2026_15958_MOESM3_ESM.zip › Supplementary Material Original Western Blot Images/Fig5 WB/Fig5G.SPINK5.tif]

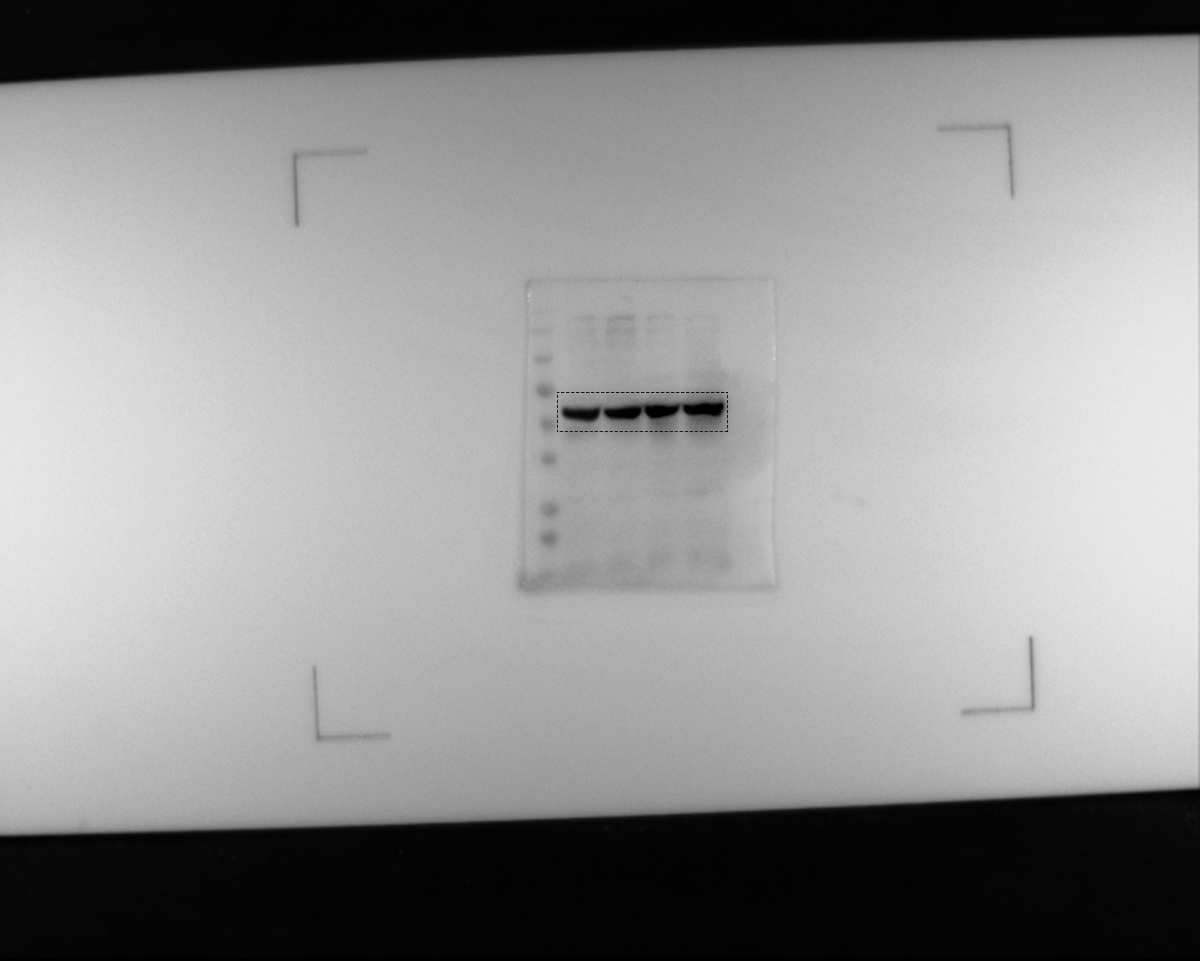

Supplement: Supplementary file 3 — Supplementary Material 3. Full-length blots/gels are presented in Supplementary Material Original Western Blot Images. [file 12885_2026_15958_MOESM3_ESM.zip › Supplementary Material Original Western Blot Images/Fig5 WB/Fig5I.Akt.tif]

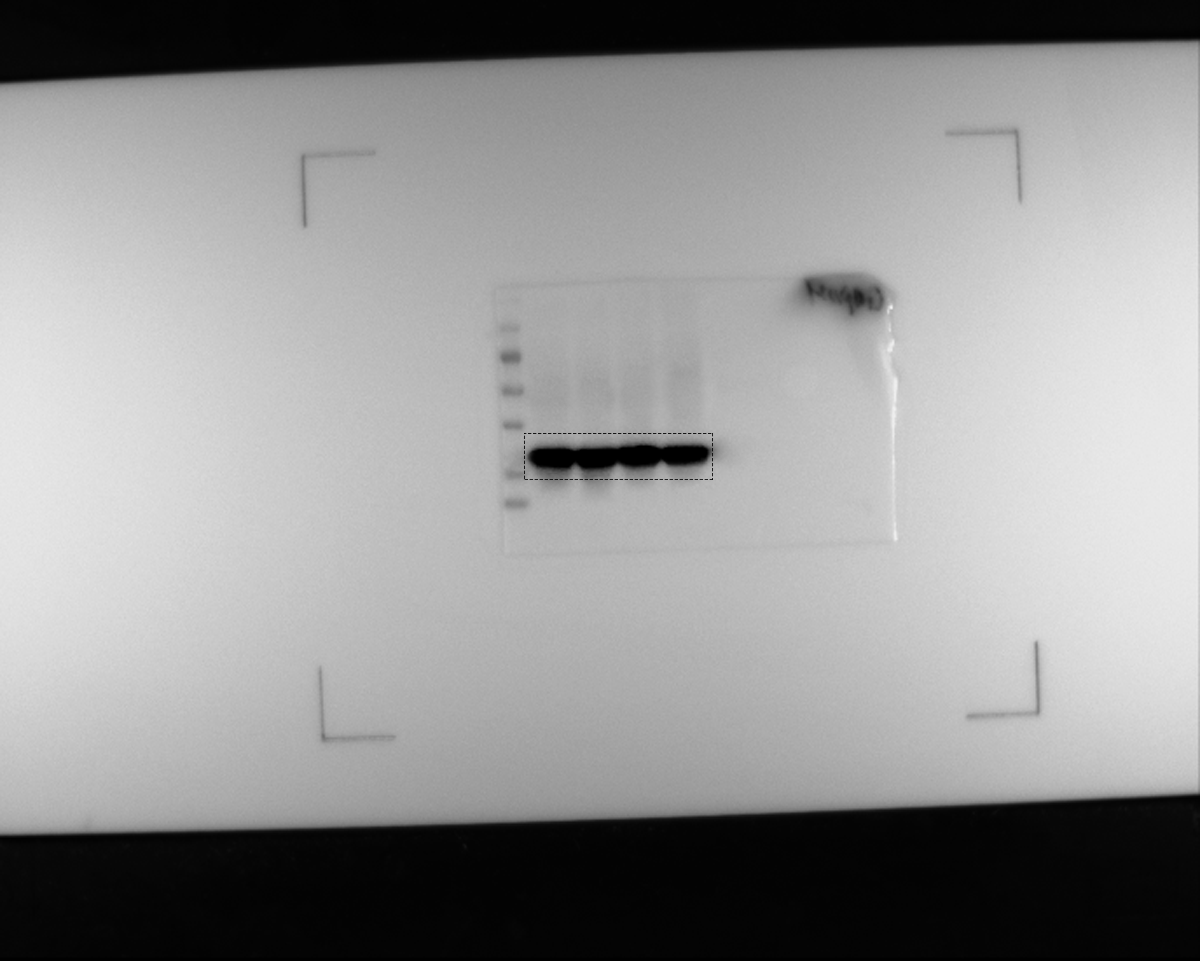

Supplement: Supplementary file 3 — Supplementary Material 3. Full-length blots/gels are presented in Supplementary Material Original Western Blot Images. [file 12885_2026_15958_MOESM3_ESM.zip › Supplementary Material Original Western Blot Images/Fig5 WB/Fig5I.GADPH.tif]

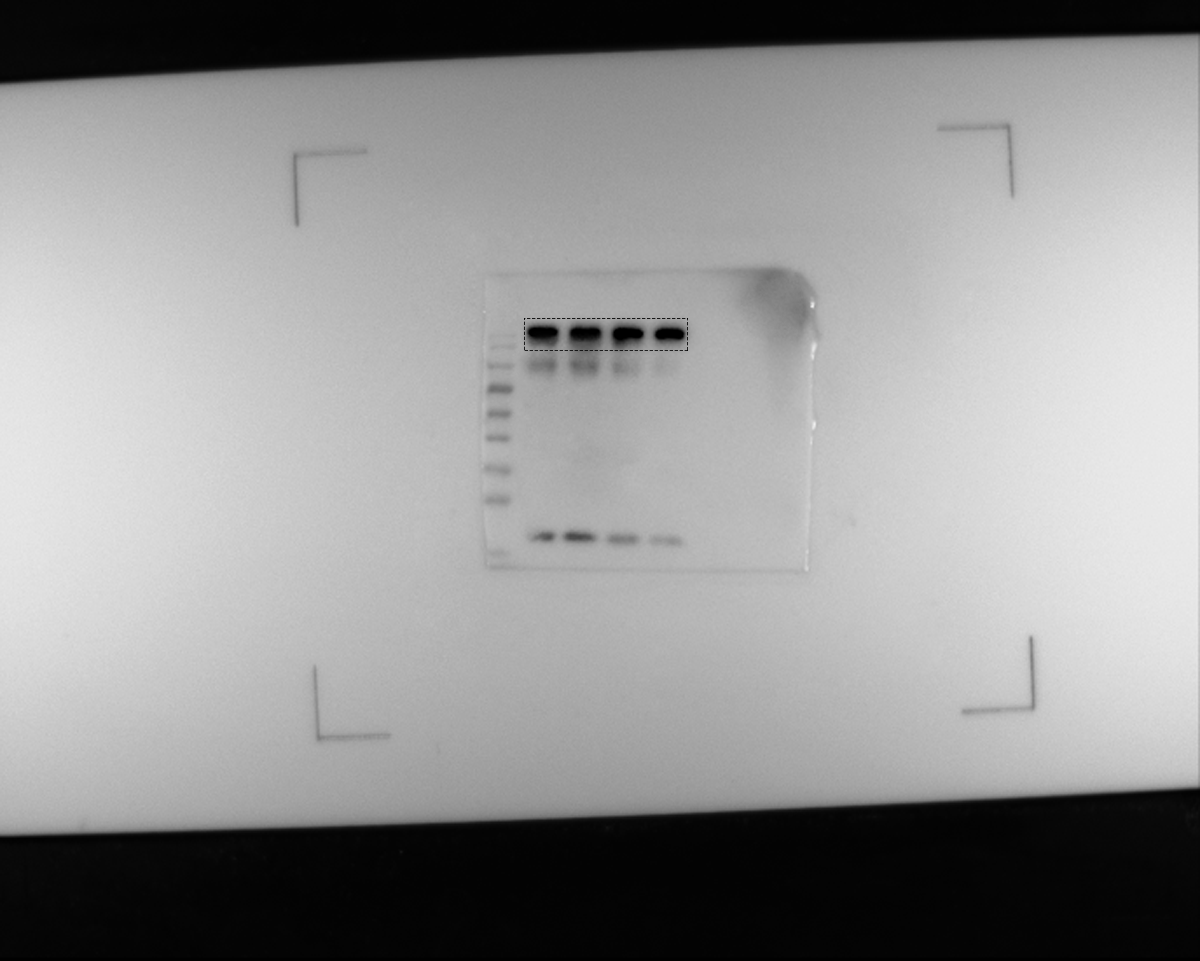

Supplement: Supplementary file 3 — Supplementary Material 3. Full-length blots/gels are presented in Supplementary Material Original Western Blot Images. [file 12885_2026_15958_MOESM3_ESM.zip › Supplementary Material Original Western Blot Images/Fig5 WB/Fig5I.mTOR.tif]

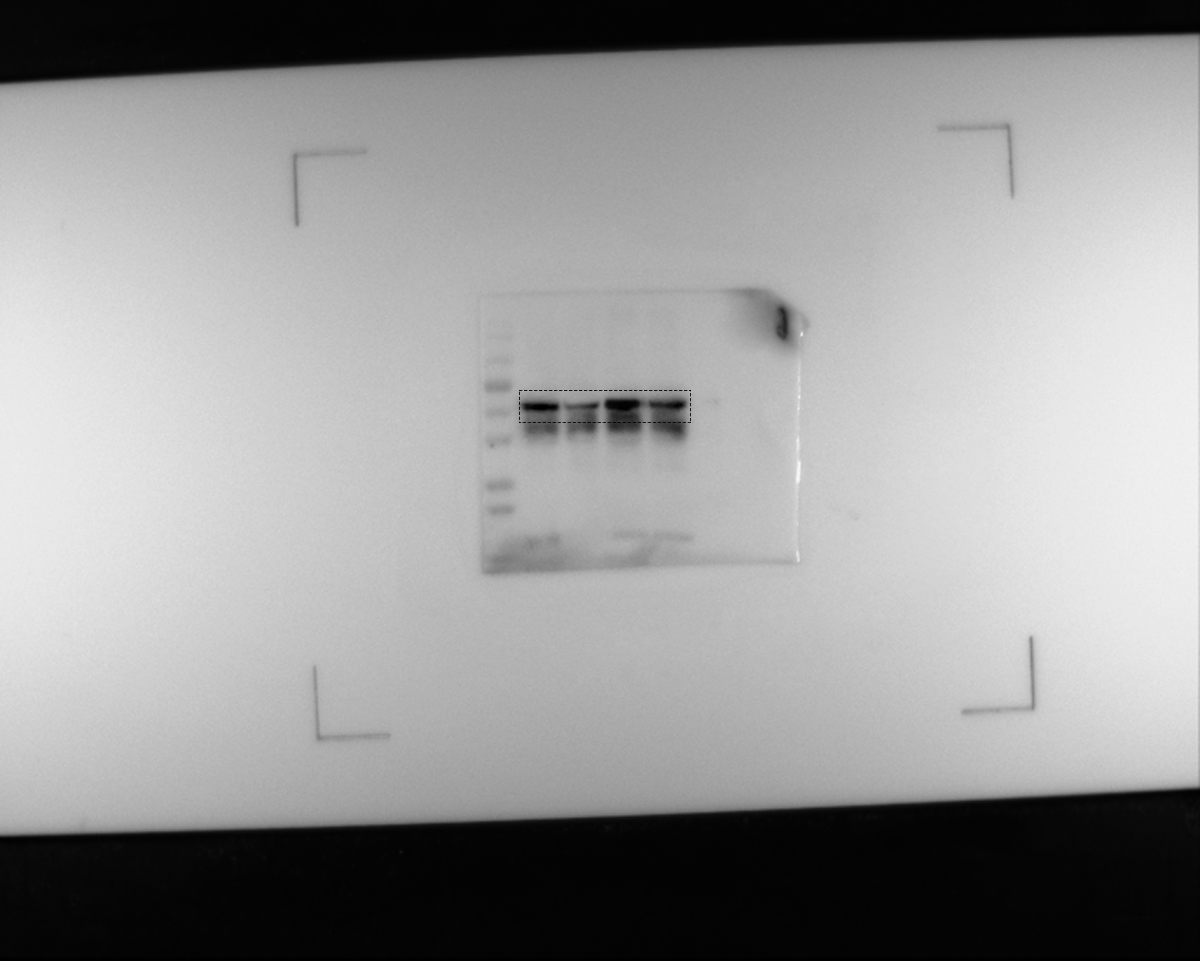

Supplement: Supplementary file 3 — Supplementary Material 3. Full-length blots/gels are presented in Supplementary Material Original Western Blot Images. [file 12885_2026_15958_MOESM3_ESM.zip › Supplementary Material Original Western Blot Images/Fig5 WB/Fig5I.p-Akt.tif]

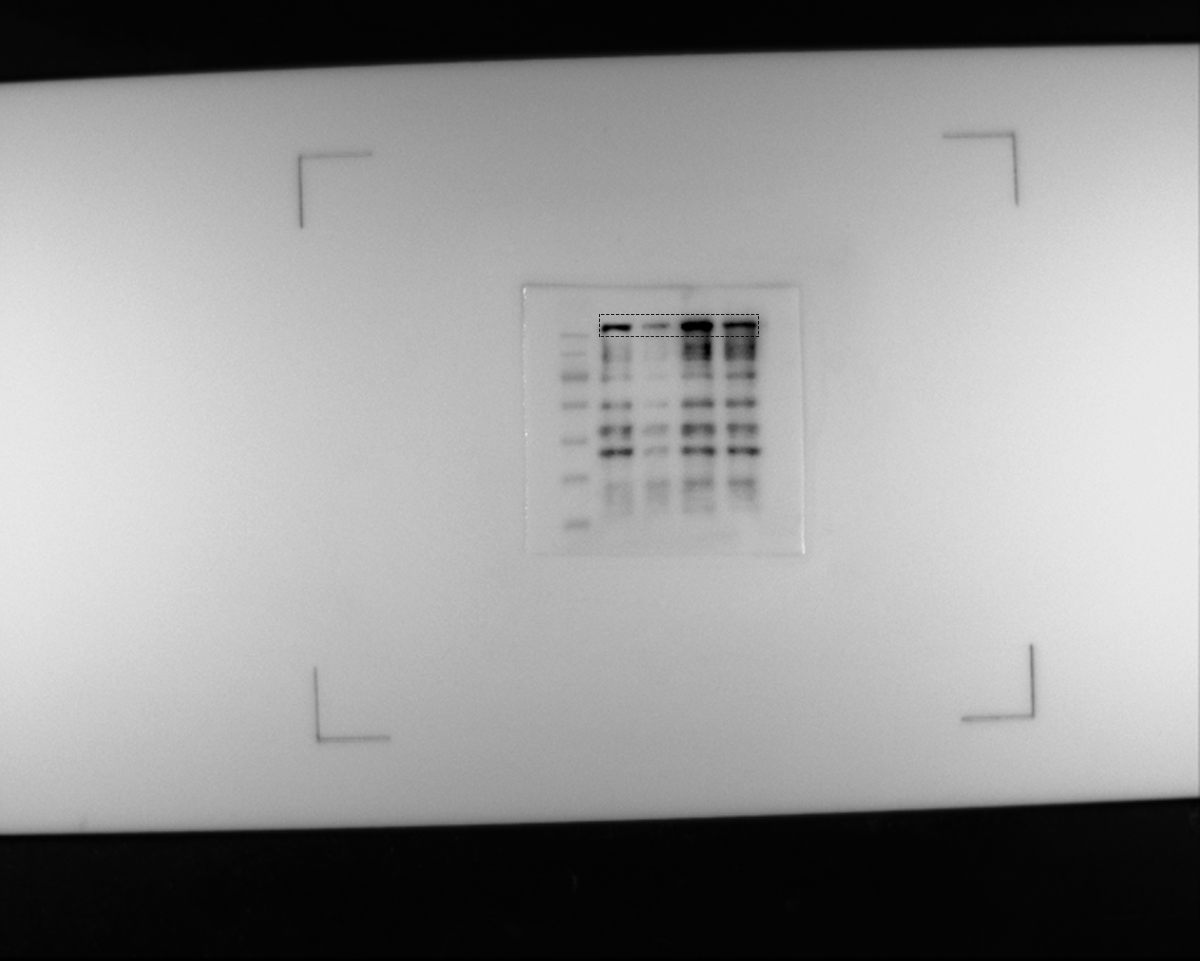

Supplement: Supplementary file 3 — Supplementary Material 3. Full-length blots/gels are presented in Supplementary Material Original Western Blot Images. [file 12885_2026_15958_MOESM3_ESM.zip › Supplementary Material Original Western Blot Images/Fig5 WB/Fig5I.p-mTOR.tif]

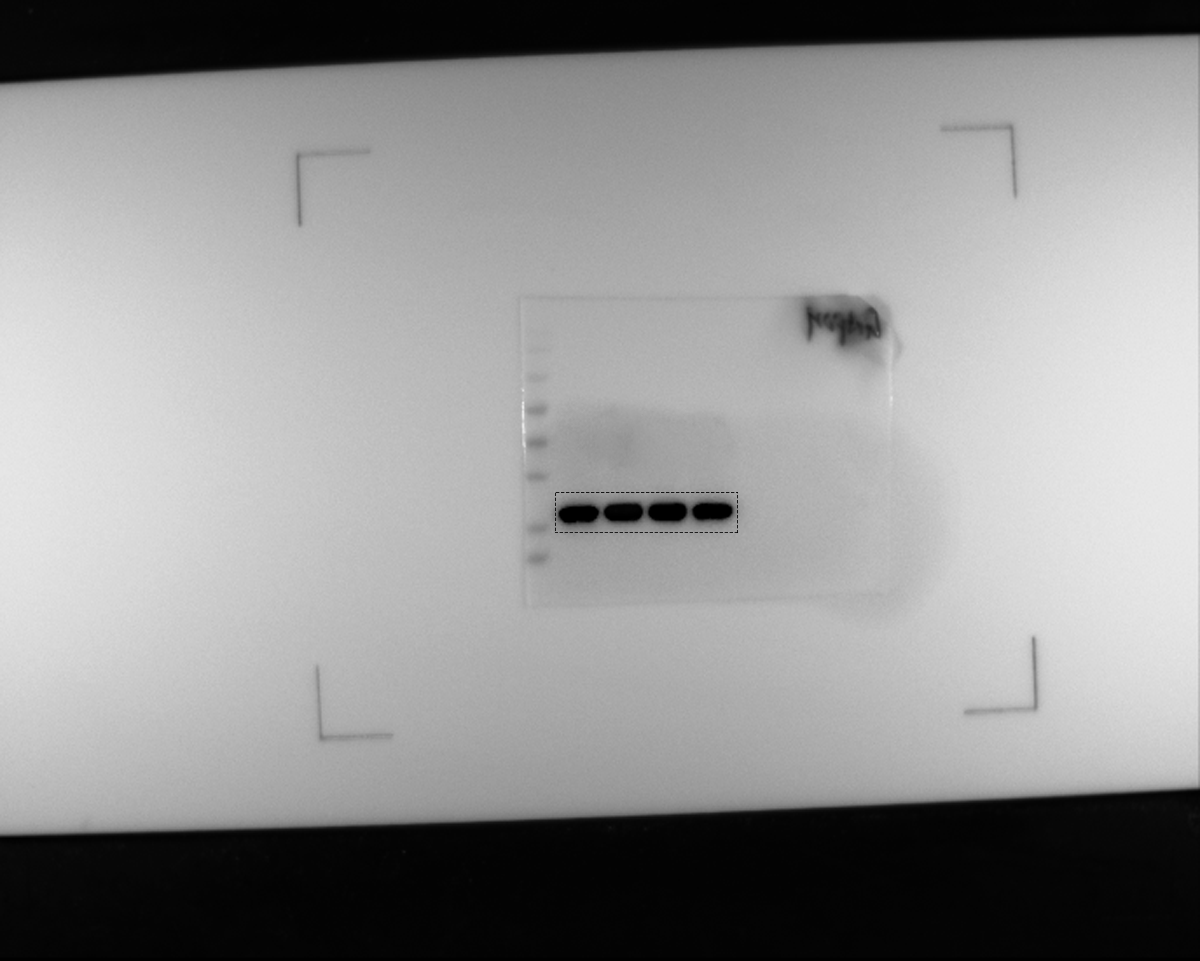

Supplement: Supplementary file 3 — Supplementary Material 3. Full-length blots/gels are presented in Supplementary Material Original Western Blot Images. [file 12885_2026_15958_MOESM3_ESM.zip › Supplementary Material Original Western Blot Images/Fig7 WB/Fig7A.GADPH.tif]

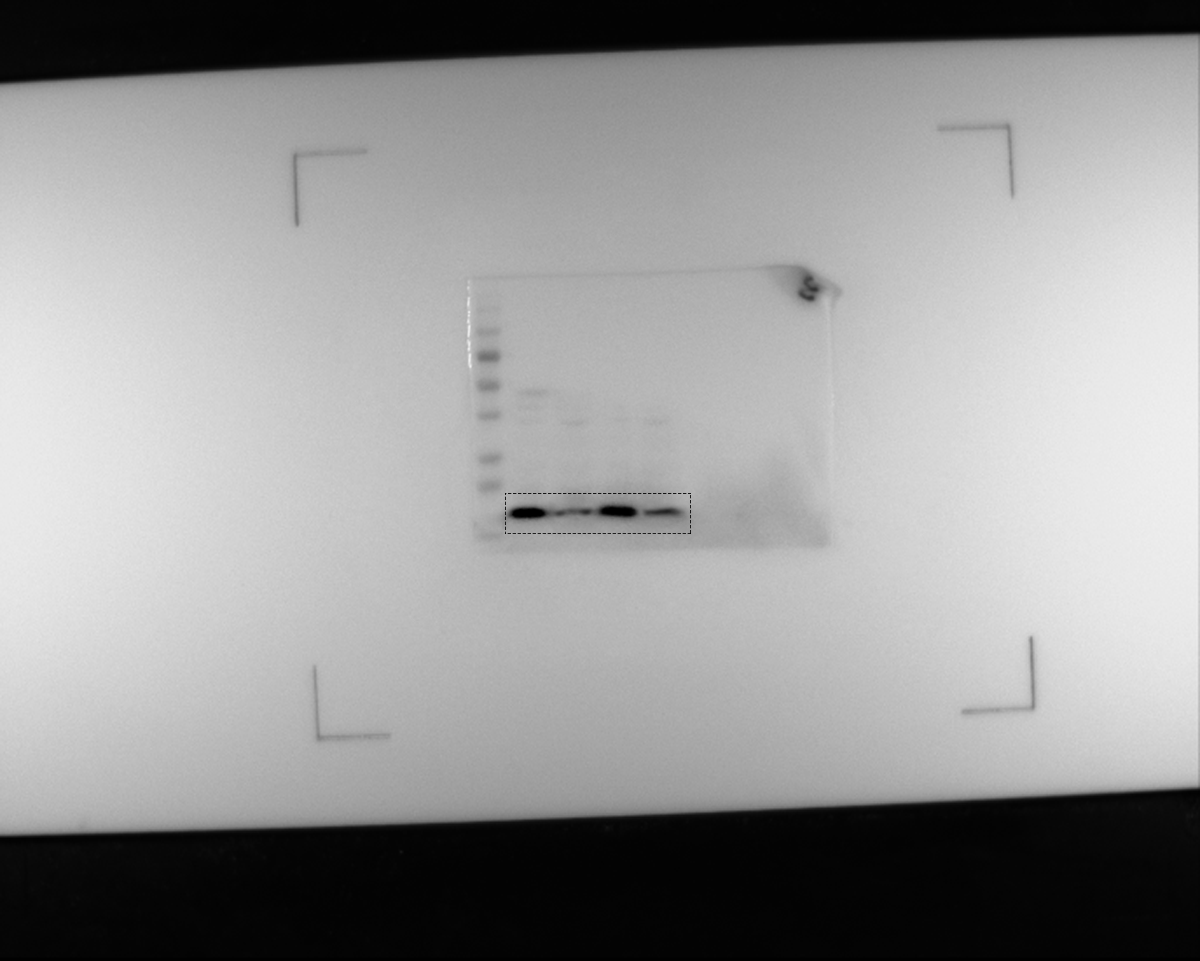

Supplement: Supplementary file 3 — Supplementary Material 3. Full-length blots/gels are presented in Supplementary Material Original Western Blot Images. [file 12885_2026_15958_MOESM3_ESM.zip › Supplementary Material Original Western Blot Images/Fig7 WB/Fig7A.KLK6.tif]

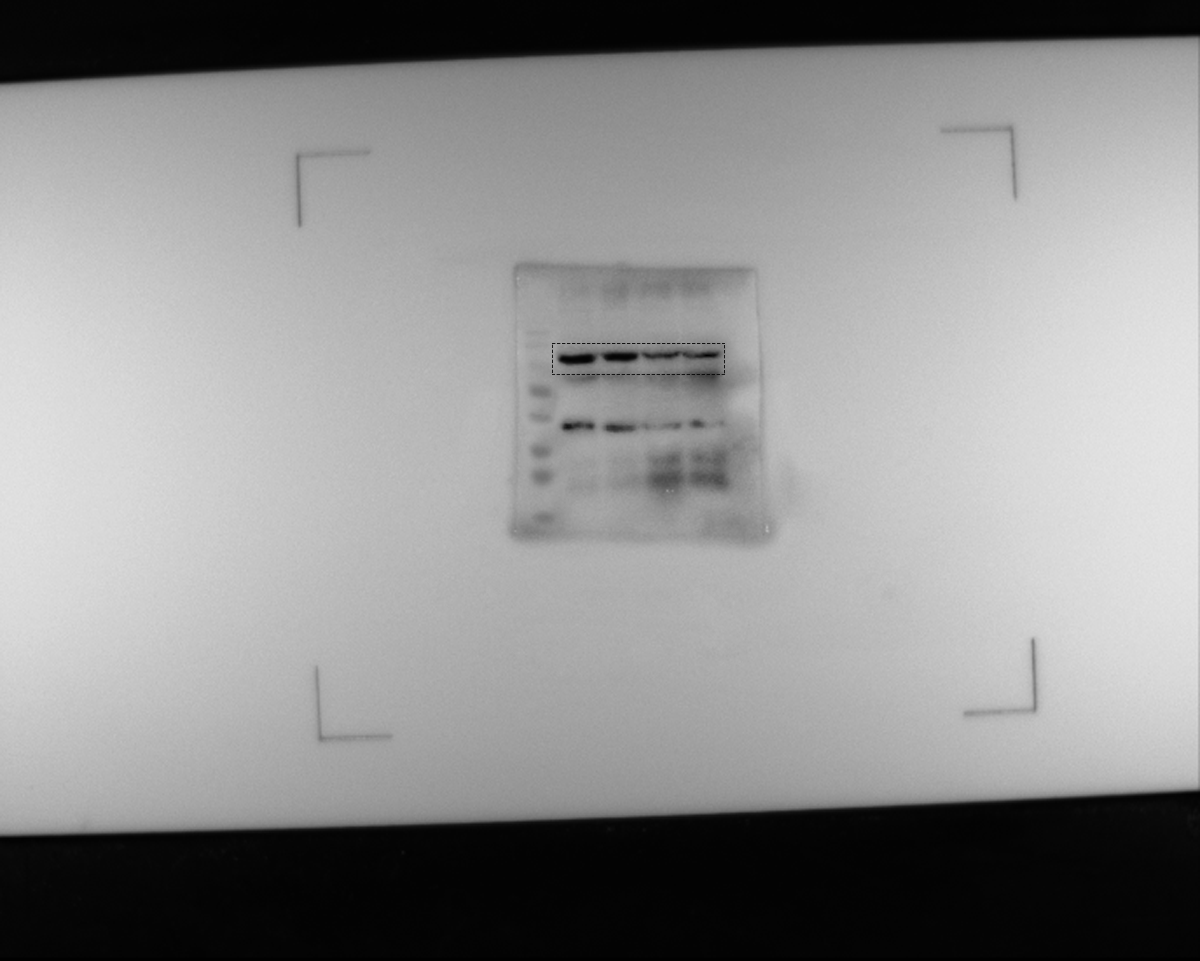

Supplement: Supplementary file 3 — Supplementary Material 3. Full-length blots/gels are presented in Supplementary Material Original Western Blot Images. [file 12885_2026_15958_MOESM3_ESM.zip › Supplementary Material Original Western Blot Images/Fig7 WB/Fig7A.SPINK5.tif]

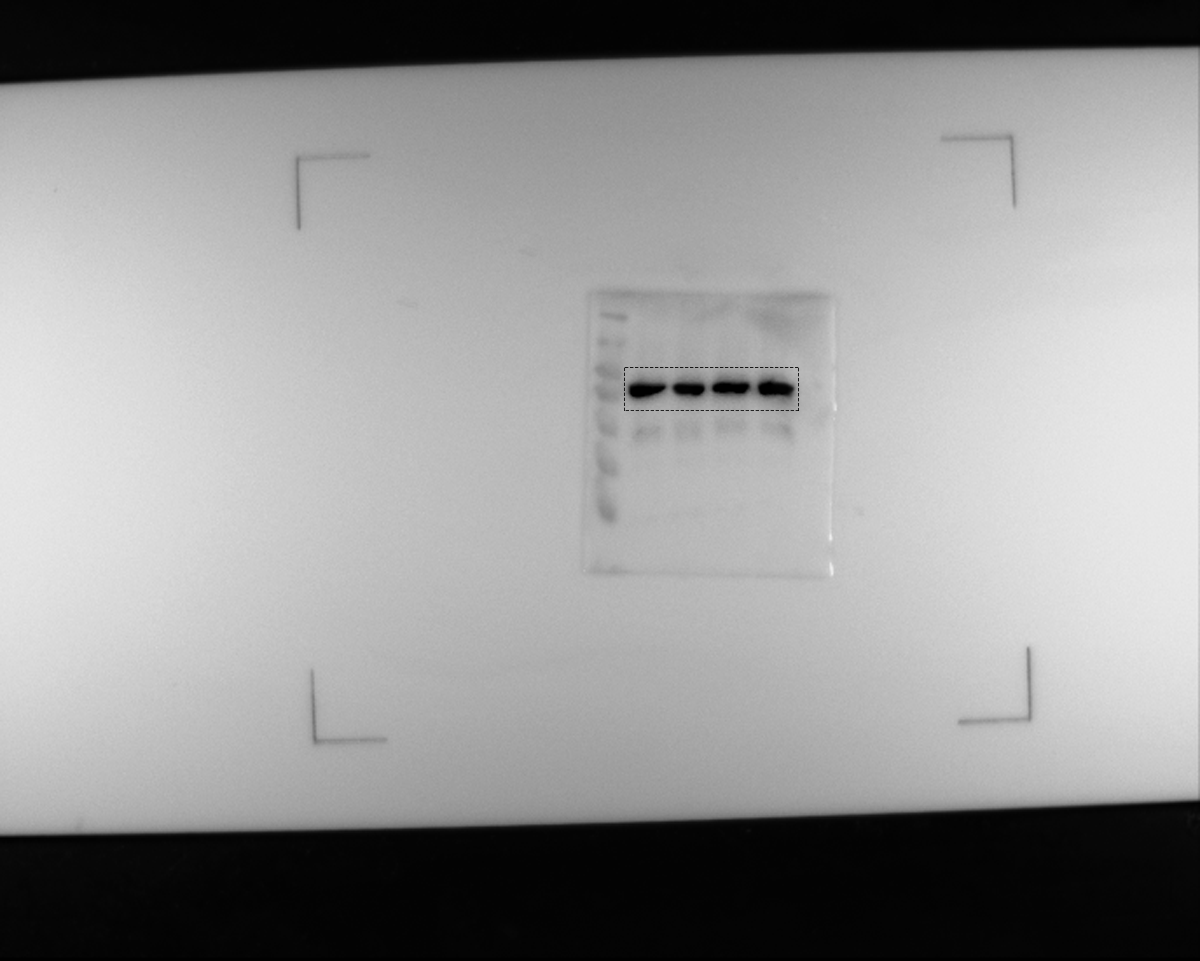

Supplement: Supplementary file 3 — Supplementary Material 3. Full-length blots/gels are presented in Supplementary Material Original Western Blot Images. [file 12885_2026_15958_MOESM3_ESM.zip › Supplementary Material Original Western Blot Images/Fig7 WB/Fig7C.Akt.tif]

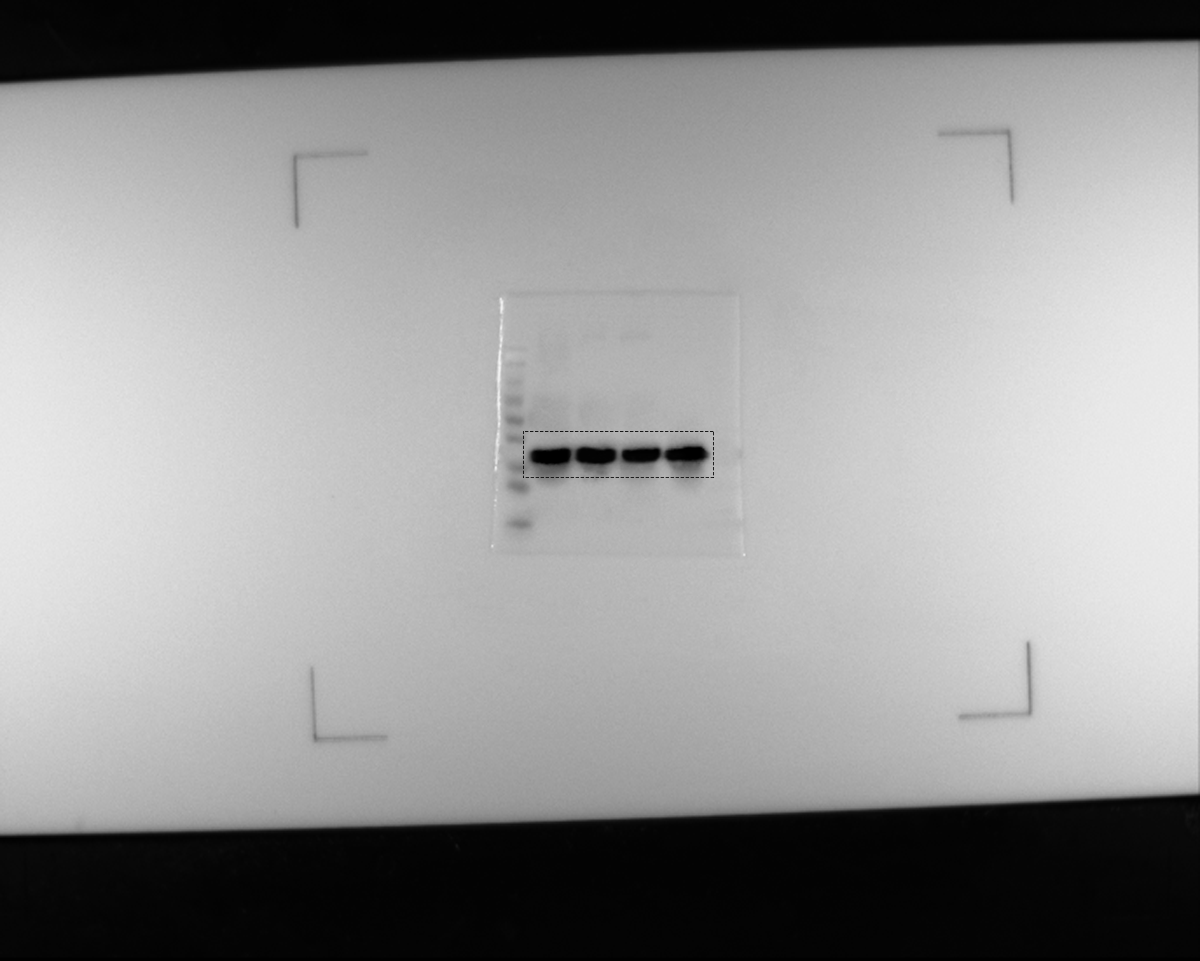

Supplement: Supplementary file 3 — Supplementary Material 3. Full-length blots/gels are presented in Supplementary Material Original Western Blot Images. [file 12885_2026_15958_MOESM3_ESM.zip › Supplementary Material Original Western Blot Images/Fig7 WB/Fig7C.GAPDH.tif]

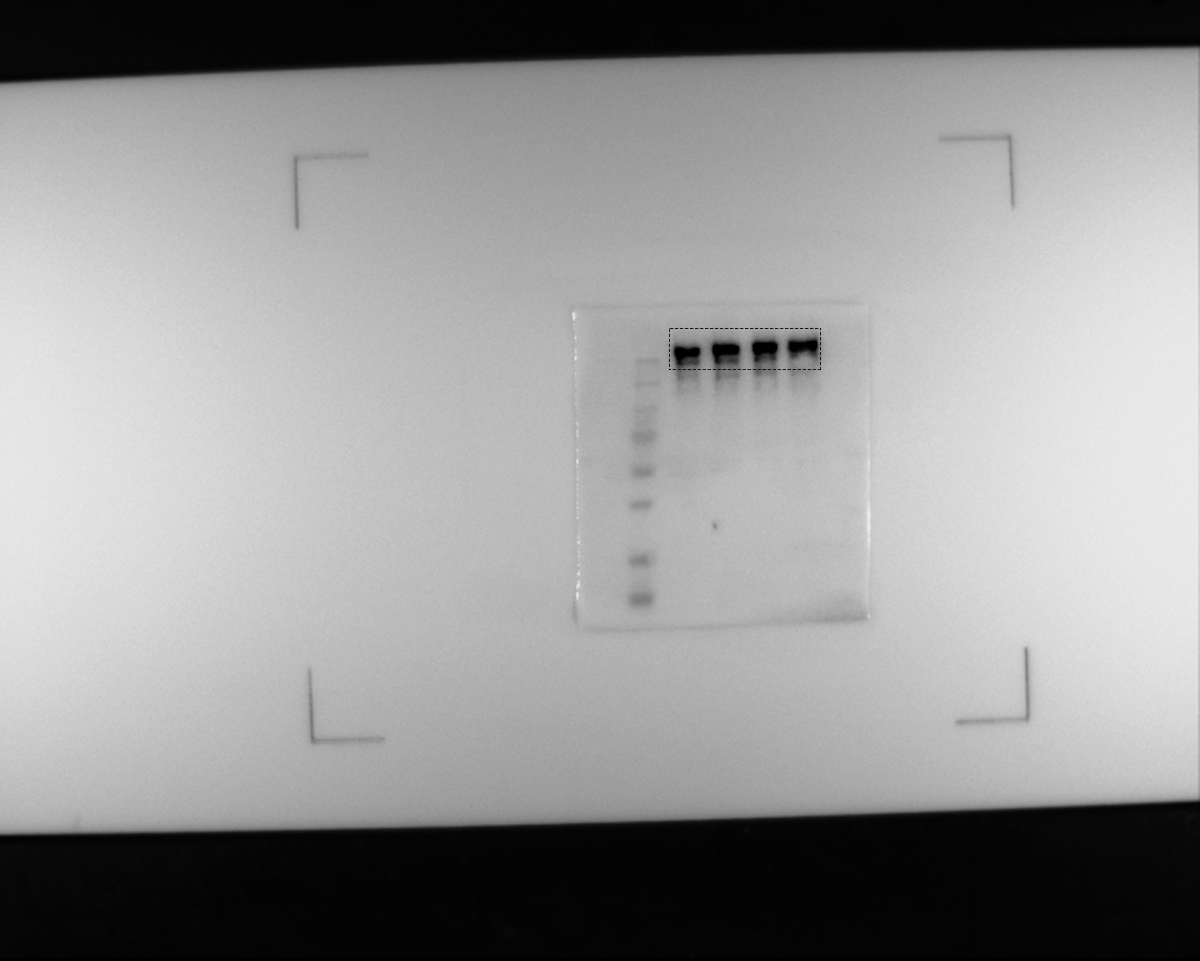

Supplement: Supplementary file 3 — Supplementary Material 3. Full-length blots/gels are presented in Supplementary Material Original Western Blot Images. [file 12885_2026_15958_MOESM3_ESM.zip › Supplementary Material Original Western Blot Images/Fig7 WB/Fig7C.mTOR.tif]

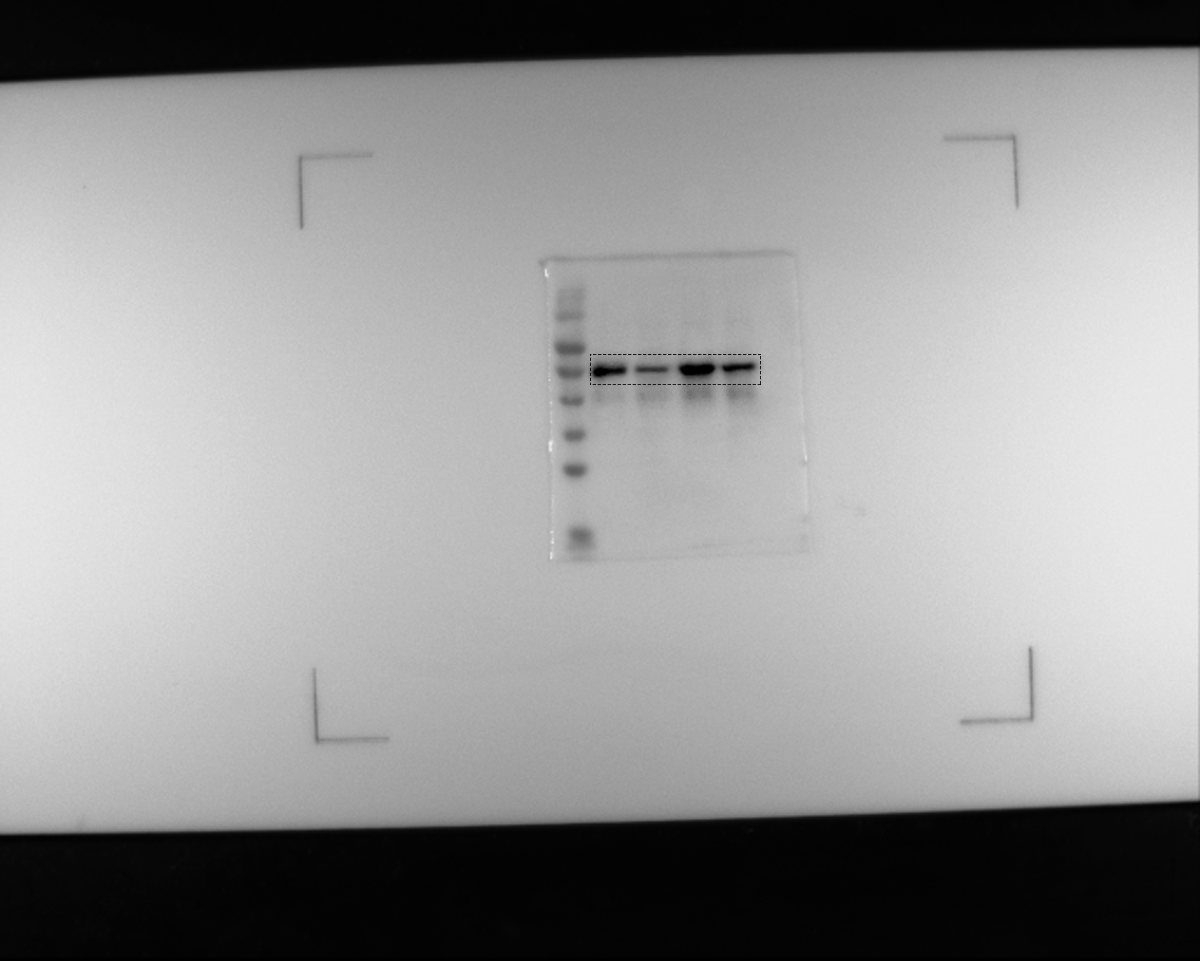

Supplement: Supplementary file 3 — Supplementary Material 3. Full-length blots/gels are presented in Supplementary Material Original Western Blot Images. [file 12885_2026_15958_MOESM3_ESM.zip › Supplementary Material Original Western Blot Images/Fig7 WB/Fig7C.p-Akt.tif]

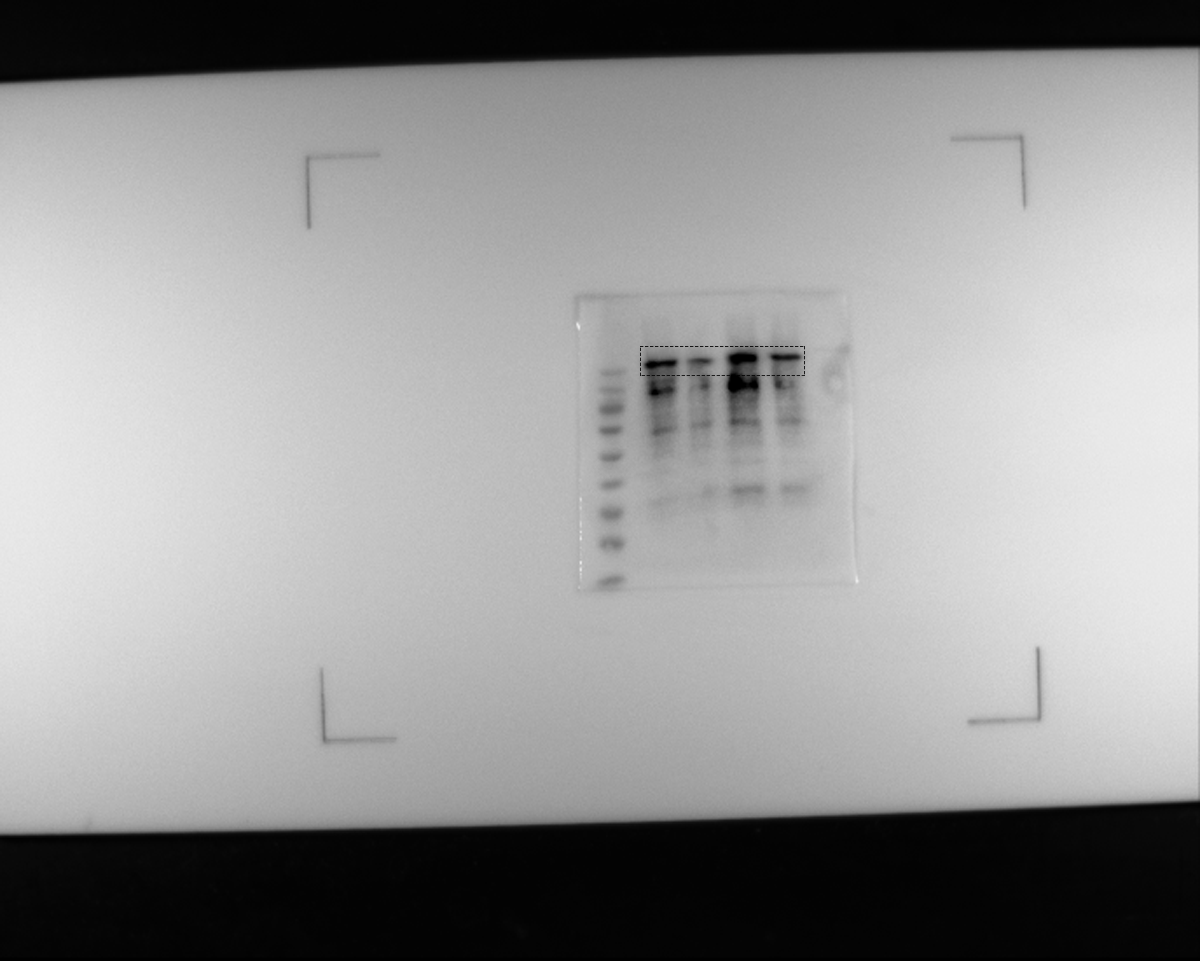

Supplement: Supplementary file 3 — Supplementary Material 3. Full-length blots/gels are presented in Supplementary Material Original Western Blot Images. [file 12885_2026_15958_MOESM3_ESM.zip › Supplementary Material Original Western Blot Images/Fig7 WB/Fig7C.p-mTOR.tif]

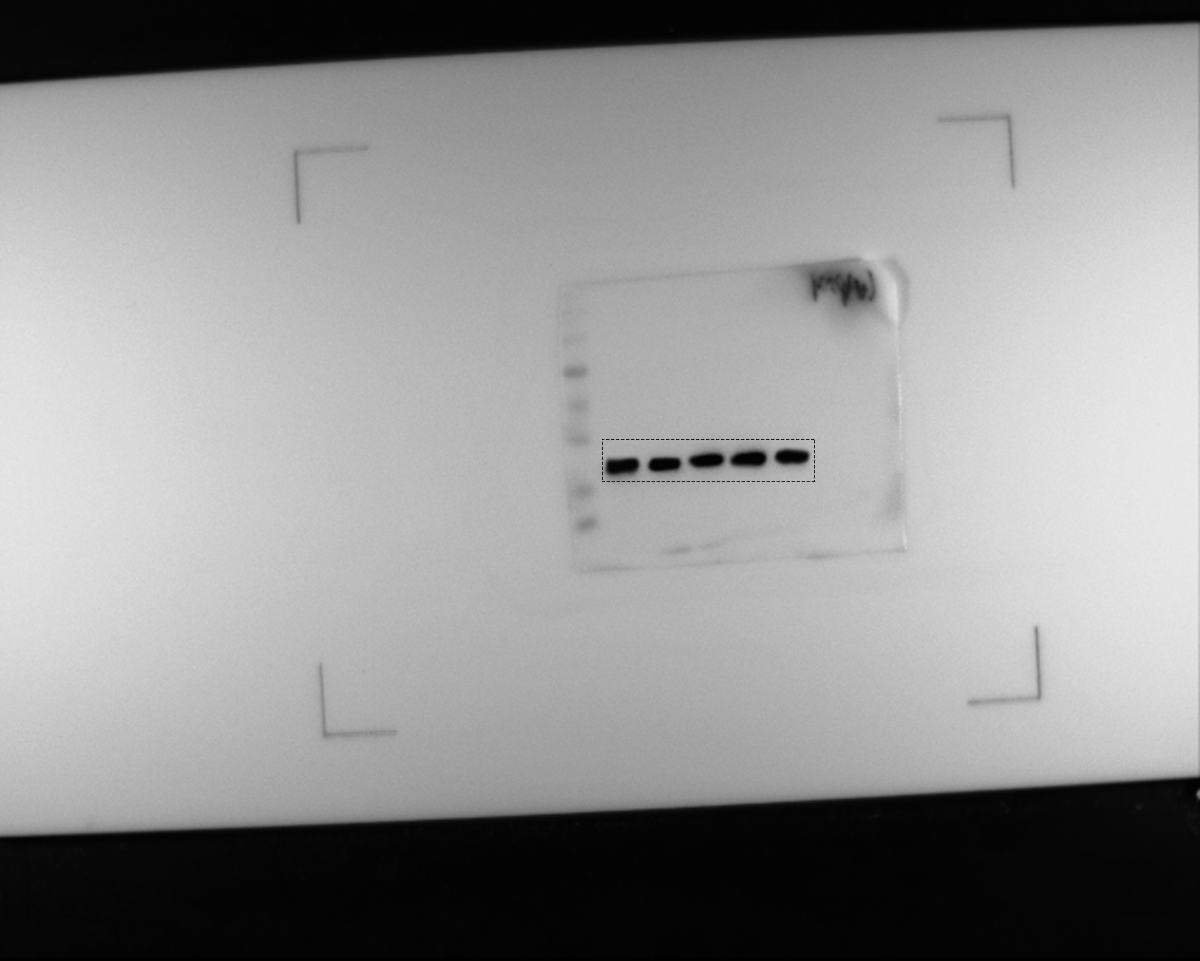

Supplement: Supplementary file 3 — Supplementary Material 3. Full-length blots/gels are presented in Supplementary Material Original Western Blot Images. [file 12885_2026_15958_MOESM3_ESM.zip › Supplementary Material Original Western Blot Images/Supplementary Fig1 WB/Supplementary Fig 1A.GAPDH.tif]

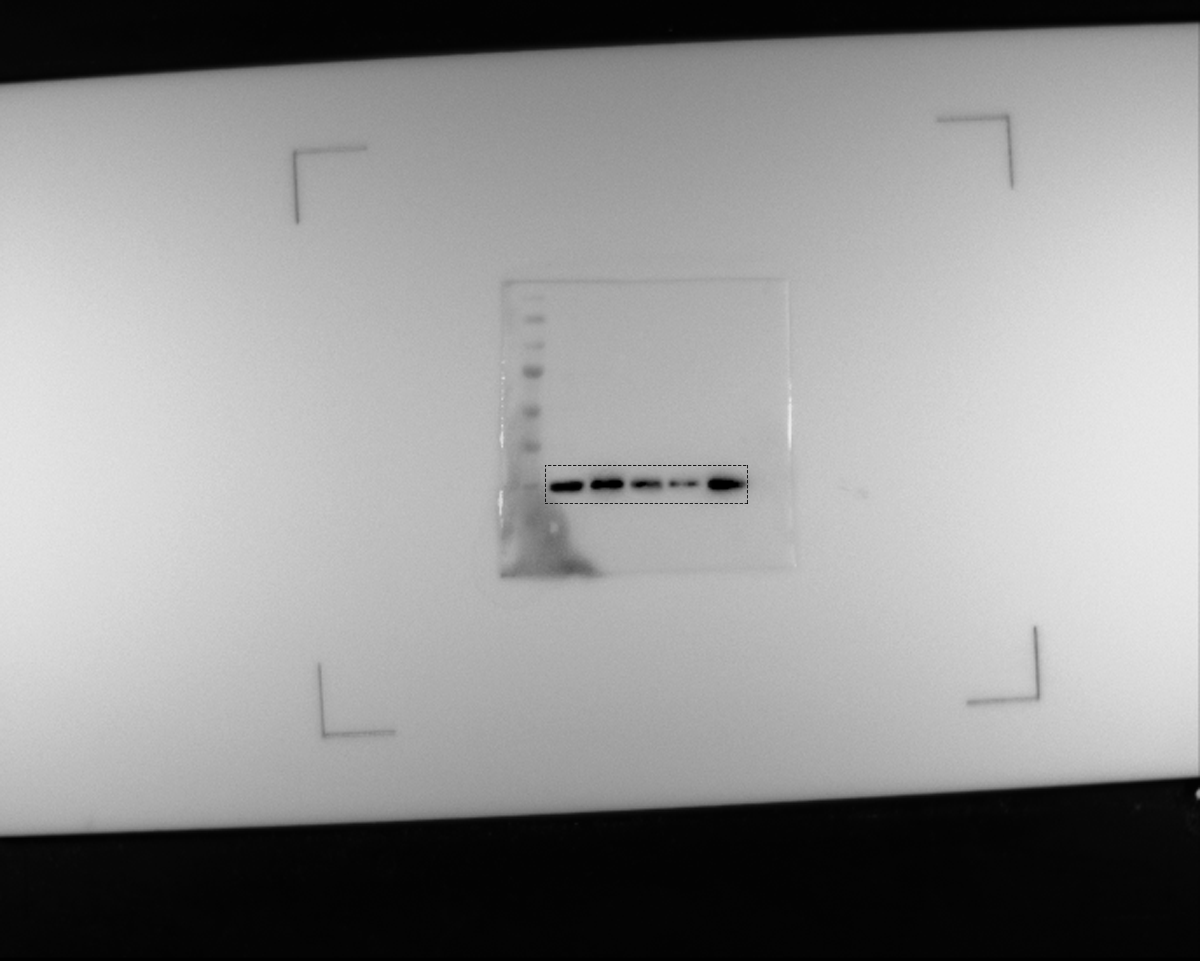

Supplement: Supplementary file 3 — Supplementary Material 3. Full-length blots/gels are presented in Supplementary Material Original Western Blot Images. [file 12885_2026_15958_MOESM3_ESM.zip › Supplementary Material Original Western Blot Images/Supplementary Fig1 WB/Supplementary Fig 1A.SPINK8.tif]

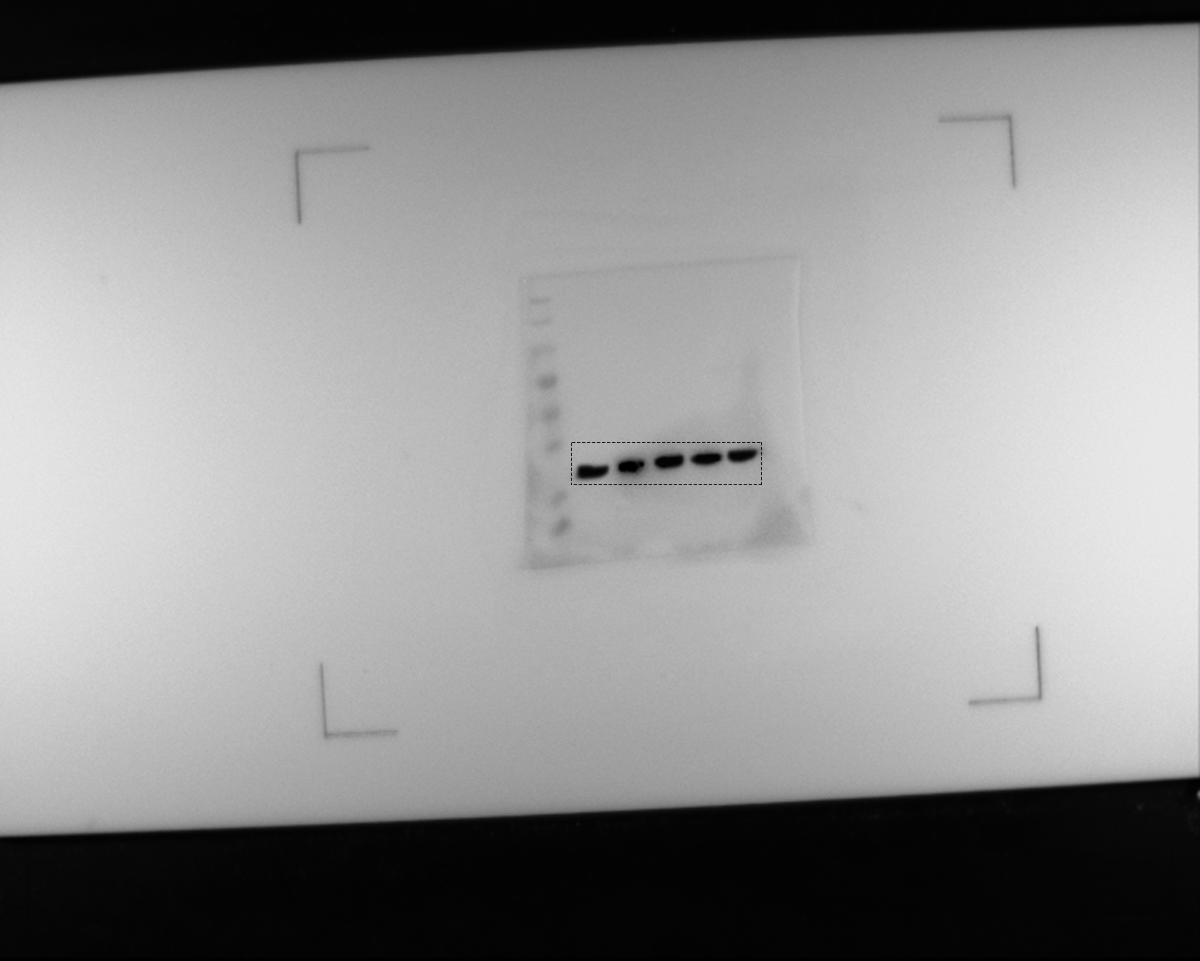

Supplement: Supplementary file 3 — Supplementary Material 3. Full-length blots/gels are presented in Supplementary Material Original Western Blot Images. [file 12885_2026_15958_MOESM3_ESM.zip › Supplementary Material Original Western Blot Images/Supplementary Fig1 WB/Supplementary Fig 1B.GAPDH.tif]

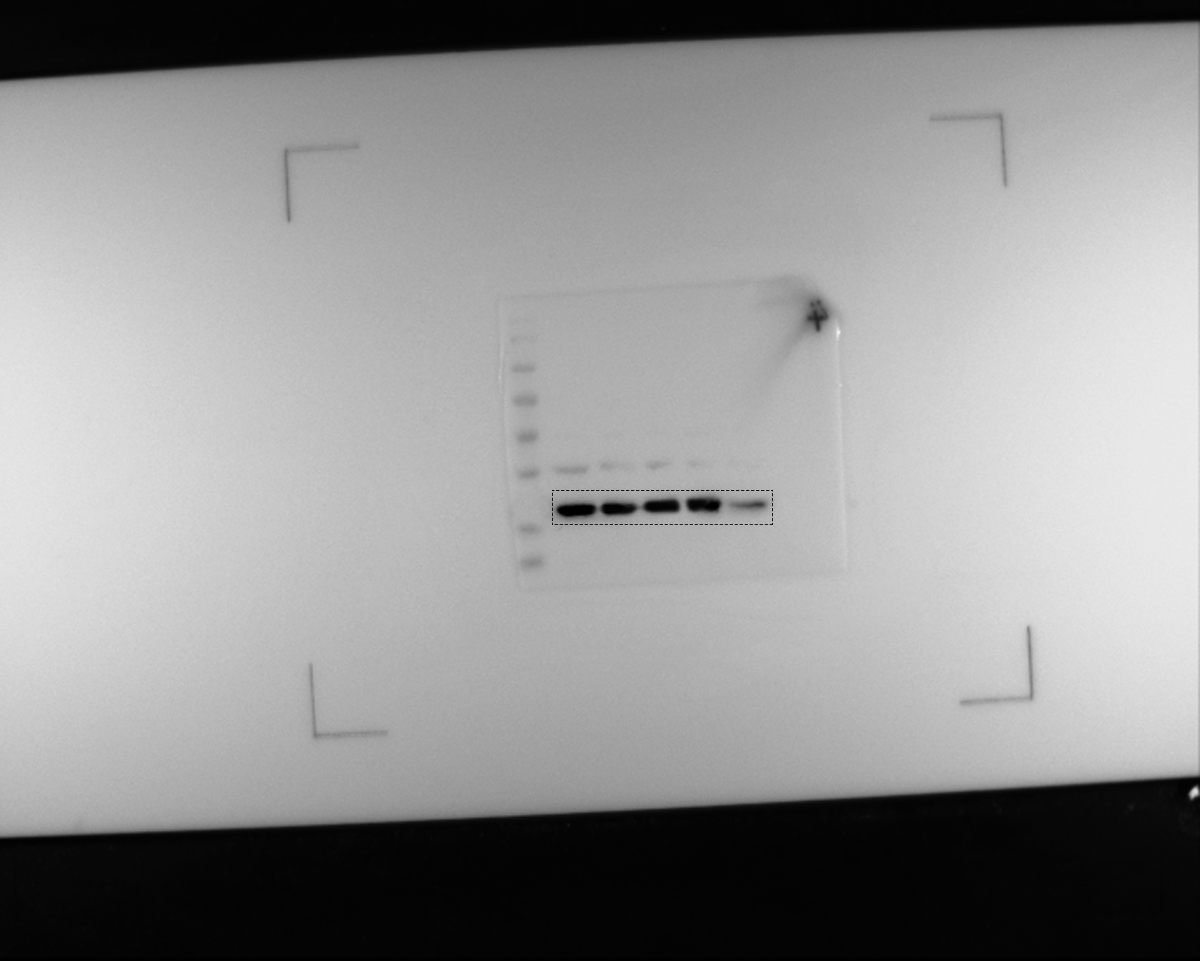

Supplement: Supplementary file 3 — Supplementary Material 3. Full-length blots/gels are presented in Supplementary Material Original Western Blot Images. [file 12885_2026_15958_MOESM3_ESM.zip › Supplementary Material Original Western Blot Images/Supplementary Fig1 WB/Supplementary Fig 1B.SPINK14.tif]
